# Supplementary material for: Identification of Endothelial Cell Protein C Receptor by Urinary Proteomics as Novel Prognostic Marker in Non-Recovery Kidney Injury
Source: Int J Mol Sci. 2024 Feb 28;25(5):2783. doi: 10.3390/ijms25052783 (PMC10931920; doi:10.3390/ijms25052783)
Supplement: Supplementary file 1 [file ijms-25-02783-s001.zip › ijms-2848765-supplementary.pdf]

115/114	116/114	Accession	Description	Σ# Unique Peptides	Σ# Peptides	Ratio: 115/114	Ratio: 116/114
		**Up-Regulated Proteins in two Subgroups (189)**					
▲	▲	Q9ULH7	MKL/myocardin-like protein 2 OS=Homo sapiens GN=MKL2 PE=1 SV=3 - [MKL2_HUMAN]	1.00	1.00	4.53	28.30
▲	▲	O75394	39S ribosomal protein L33, mitochondrial OS=Homo sapiens GN=MRPL33 PE=1 SV=1 - [RM33_HUMAN]	1.00	1.00	23.64	26.69
▲	▲	P16860	Natriuretic peptides B OS=Homo sapiens GN=NPPB PE=1 SV=1 - [ANFB_HUMAN]	1.00	1.00	7.78	19.48
▲	▲	P35611	Alpha-adducin OS=Homo sapiens GN=ADD1 PE=1 SV=2 - [ADDA_HUMAN]	1.00	1.00	2.53	18.36
▲	▲	Q9NS71	Gastrokine-1 OS=Homo sapiens GN=GKN1 PE=2 SV=3 - [GKN1_HUMAN]	2.00	2.00	2.35	13.64
▲	▲	Q9BX66	Sorbin and SH3 domain-containing protein 1 OS=Homo sapiens GN=SORBS1 PE=1 SV=2 - [SRBS1_HUMAN	1.00	1.00	2.71	13.61
▲	▲	P63313	Thymosin beta-10 OS=Homo sapiens GN=TMSB10 PE=1 SV=2 - [TYB10_HUMAN]	1.00	2.00	2.08	13.27
▲	▲	Q15063	Periostin OS=Homo sapiens GN=POSTN PE=1 SV=2 - [POSTN_HUMAN]	3.00	3.00	2.69	12.77
▲	▲	P13747	HLA class I histocompatibility antigen, alpha chain E OS=Homo sapiens GN=HLA-E PE=1 SV=3 - [HLAE_HU	1.00	2.00	2.84	12.62
▲	▲	P61626	Lysozyme C OS=Homo sapiens GN=LYZ PE=1 SV=1 - [LYSC_HUMAN]	6.00	6.00	2.83	12.61
▲	▲	Q0D2I5	Intermediate filament family orphan 1 OS=Homo sapiens GN=IFFO1 PE=2 SV=2 - [IFFO1_HUMAN]	1.00	1.00	5.75	12.49
▲	▲	Q9NPD7	Neuritin OS=Homo sapiens GN=NRN1 PE=1 SV=1 - [NRN1_HUMAN]	1.00	1.00	3.24	11.70
▲	▲	Q9P035	Protein tyrosine phosphatase-like protein PTPLAD1 OS=Homo sapiens GN=PTPLAD1 PE=1 SV=2 - [PTAD1_	1.00	1.00	3.13	11.53
▲	▲	A8MW06	Thymosin beta-4-like protein 3 OS=Homo sapiens GN=TMSL3 PE=2 SV=1 - [TMSL3_HUMAN]	2.00	3.00	4.00	11.53
▲	▲	P51161	Gastrotropin OS=Homo sapiens GN=FABP6 PE=1 SV=2 - [FABP6_HUMAN]	6.00	6.00	3.40	11.18
▲	▲	P01178	Oxytocin-neurophysin 1 OS=Homo sapiens GN=OXT PE=1 SV=1 - [NEU1_HUMAN]	1.00	2.00	4.99	11.18
▲	▲	P02652	Apolipoprotein A-II OS=Homo sapiens GN=APOA2 PE=1 SV=1 - [APOA2_HUMAN]	7.00	7.00	2.84	11.09
▲	▲	P06727	Apolipoprotein A-IV OS=Homo sapiens GN=APOA4 PE=1 SV=3 - [APOA4_HUMAN]	26.00	26.00	3.46	10.85
▲	▲	P02647	Apolipoprotein A-I OS=Homo sapiens GN=APOA1 PE=1 SV=1 - [APOA1_HUMAN]	24.00	24.00	2.05	10.83
▲	▲	Q16627	C-C motif chemokine 14 OS=Homo sapiens GN=CCL14 PE=1 SV=1 - [CCL14_HUMAN]	3.00	3.00	2.18	10.21
▲	▲	P05976	Myosin light chain 1/3, skeletal muscle isoform OS=Homo sapiens GN=MYL1 PE=1 SV=3 - [MYL1_HUMAN	1.00	2.00	2.23	10.16
▲	▲	Q01523	Defensin-5 OS=Homo sapiens GN=DEFA5 PE=1 SV=1 - [DEF5_HUMAN]	1.00	1.00	2.98	9.74
▲	▲	P35542	Serum amyloid A-4 protein OS=Homo sapiens GN=SAA4 PE=1 SV=2 - [SAA4_HUMAN]	3.00	3.00	2.05	9.74
▲	▲	P11226	Mannose-binding protein C OS=Homo sapiens GN=MBL2 PE=1 SV=2 - [MBL2_HUMAN]	2.00	2.00	2.42	9.56
▲	▲	Q96S66	Chloride channel CLIC-like protein 1 OS=Homo sapiens GN=CLCC1 PE=1 SV=1 - [CLCC1_HUMAN]	1.00	1.00	2.22	9.36
▲	▲	P02679	Fibrinogen gamma chain OS=Homo sapiens GN=FGG PE=1 SV=3 - [FIBG_HUMAN]	14.00	14.00	3.27	9.36
▲	▲	P24387	Corticotropin-releasing factor-binding protein OS=Homo sapiens GN=CRHBP PE=1 SV=2 - [CRHBP_HUMAN	3.00	3.00	2.88	9.22
▲	▲	P51858	Hepatoma-derived growth factor OS=Homo sapiens GN=HDGF PE=1 SV=1 - [HDGF_HUMAN]	3.00	3.00	2.50	9.02
▲	▲	Q9P1F3	UPF0727 protein C6orf115 OS=Homo sapiens GN=C6orf115 PE=1 SV=1 - [CF115_HUMAN]	2.00	2.00	2.87	8.41
▲	▲	P05413	Fatty acid-binding protein, heart OS=Homo sapiens GN=FABP3 PE=1 SV=4 - [FABPH_HUMAN]	11.00	11.00	3.34	8.19
▲	▲	P00751	Complement factor B OS=Homo sapiens GN=CFB PE=1 SV=2 - [CFAB_HUMAN]	30.00	30.00	2.09	8.16
▲	▲	P59665	Neutrophil defensin 1 OS=Homo sapiens GN=DEFA1 PE=1 SV=1 - [DEF1_HUMAN]	5.00	5.00	3.29	7.90

▲	▲	P36955	Pigment epithelium-derived factor OS=Homo sapiens GN=SERPINF1 PE=1 SV=3 - [PEDF_HUMAN]	20.00	20.00	2.54	7.84
▲	▲	P01034	Cystatin-C OS=Homo sapiens GN=CST3 PE=1 SV=1 - [CYTC_HUMAN]	9.00	9.00	2.12	7.73
▲	▲	P02748	Complement component C9 OS=Homo sapiens GN=C9 PE=1 SV=2 - [CO9_HUMAN]	17.00	17.00	2.08	7.53
▲	▲	P02144	Myoglobin OS=Homo sapiens GN=MB PE=1 SV=2 - [MYG_HUMAN]	9.00	9.00	2.64	7.51
▲	▲	Q08554	Desmocollin-1 OS=Homo sapiens GN=DSC1 PE=1 SV=2 - [DSC1_HUMAN]	4.00	4.00	2.84	7.38
▲	▲	P23083	Ig heavy chain V-I region V35 OS=Homo sapiens PE=1 SV=1 - [HV103_HUMAN]	2.00	3.00	2.73	7.32
▲	▲	Q8WXD2	Secretogranin-3 OS=Homo sapiens GN=SCG3 PE=1 SV=3 - [SCG3_HUMAN]	1.00	1.00	2.20	7.27
▲	▲	P01344	Insulin-like growth factor II OS=Homo sapiens GN=IGF2 PE=1 SV=1 - [IGF2_HUMAN]	4.00	5.00	4.15	7.12
▲	▲	P36980	Complement factor H-related protein 2 OS=Homo sapiens GN=CFHR2 PE=1 SV=1 - [FHR2_HUMAN]	4.00	8.00	3.86	7.11
▲	▲	Q5JU67	Uncharacterized protein C9orf117 OS=Homo sapiens GN=C9orf117 PE=2 SV=1 - [CI117_HUMAN]	1.00	1.00	5.98	7.10
▲	▲	Q96IY4	Carboxypeptidase B2 OS=Homo sapiens GN=CPB2 PE=1 SV=1 - [CBPB2_HUMAN]	2.00	2.00	2.43	7.08
▲	▲	Q9UNN5	FAS-associated factor 1 OS=Homo sapiens GN=FAF1 PE=1 SV=2 - [FAF1_HUMAN]	1.00	1.00	2.38	7.04
▲	▲	P28300	Protein-lysine 6-oxidase OS=Homo sapiens GN=LOX PE=1 SV=2 - [LYOX_HUMAN]	3.00	3.00	3.15	7.03
▲	▲	Q9UGM5	Fetuin-B OS=Homo sapiens GN=FETUB PE=1 SV=2 - [FETUB_HUMAN]	8.00	8.00	2.93	7.03
▲	▲	P04080	Cystatin-B OS=Homo sapiens GN=CSTB PE=1 SV=2 - [CYTB_HUMAN]	6.00	6.00	3.16	6.93
▲	▲	P02775	Platelet basic protein OS=Homo sapiens GN=PPBP PE=1 SV=3 - [CXCL7_HUMAN]	3.00	3.00	3.00	6.88
▲	▲	P27169	Serum paraoxonase/arylesterase 1 OS=Homo sapiens GN=PON1 PE=1 SV=2 - [PON1_HUMAN]	5.00	5.00	3.00	6.85
▲	▲	Q96KK5	Histone H2A type 1-H OS=Homo sapiens GN=HIST1H2AH PE=1 SV=3 - [H2A1H_HUMAN]	1.00	1.00	3.09	6.69
▲	▲	P14621	Acylphosphatase-2 OS=Homo sapiens GN=ACYP2 PE=1 SV=2 - [ACYP2_HUMAN]	3.00	3.00	2.22	6.64
▲	▲	P05160	Coagulation factor XIII B chain OS=Homo sapiens GN=F13B PE=1 SV=3 - [F13B_HUMAN]	10.00	10.00	2.52	6.55
▲	▲	P27918	Properdin OS=Homo sapiens GN=CFP PE=1 SV=2 - [PROP_HUMAN]	1.00	1.00	3.06	6.51
▲	▲	Q9NQ38	Serine protease inhibitor Kazal-type 5 OS=Homo sapiens GN=SPINK5 PE=1 SV=2 - [ISK5_HUMAN]	25.00	25.00	3.33	6.50
▲	▲	O14556	Glyceraldehyde-3-phosphate dehydrogenase, testis-specific OS=Homo sapiens GN=GAPDHS PE=1 SV=2 - [G3	1.00	1.00	3.35	6.41
▲	▲	P08311	Cathepsin G OS=Homo sapiens GN=CTSG PE=1 SV=2 - [CATG_HUMAN]	2.00	2.00	2.21	6.38
▲	▲	P02675	Fibrinogen beta chain OS=Homo sapiens GN=FGB PE=1 SV=2 - [FIBB_HUMAN]	18.00	18.00	2.91	6.31
▲	▲	Q9ULZ3	Apoptosis-associated speck-like protein containing a CARD OS=Homo sapiens GN=PYCARD PE=1 SV=2 - [A	6.00	6.00	2.37	6.18
▲	▲	P01024	Complement C3 OS=Homo sapiens GN=C3 PE=1 SV=2 - [CO3_HUMAN]	101.00	101.00	2.16	6.17
▲	▲	P48304	Lithostathine-1-beta OS=Homo sapiens GN=REG1B PE=1 SV=1 - [REG1B_HUMAN]	4.00	8.00	8.47	6.14
▲	▲	Q9H2X0	Chordin OS=Homo sapiens GN=CHRD PE=1 SV=2 - [CHRD_HUMAN]	1.00	1.00	2.04	6.08
▲	▲	P19827	Inter-alpha-trypsin inhibitor heavy chain H1 OS=Homo sapiens GN=ITIH1 PE=1 SV=3 - [ITIH1_HUMAN]	13.00	13.00	2.17	6.06
▲	▲	P02746	Complement C1q subcomponent subunit B OS=Homo sapiens GN=C1QB PE=1 SV=2 - [C1QB_HUMAN]	1.00	1.00	2.09	6.05
▲	▲	P02774	Vitamin D-binding protein OS=Homo sapiens GN=GC PE=1 SV=1 - [VTDB_HUMAN]	36.00	36.00	2.12	5.89
▲	▲	P62805	Histone H4 OS=Homo sapiens GN=HIST1H4A PE=1 SV=2 - [H4_HUMAN]	4.00	4.00	2.11	5.87
▲	▲	P15090	Fatty acid-binding protein, adipocyte OS=Homo sapiens GN=FABP4 PE=1 SV=3 - [FABP4_HUMAN]	8.00	8.00	3.74	5.80
▲	▲	P58166	Inhibin beta E chain OS=Homo sapiens GN=INHBE PE=1 SV=1 - [INHBE_HUMAN]	1.00	1.00	2.41	5.73
▲	▲	O60575	Serine protease inhibitor Kazal-type 4 OS=Homo sapiens GN=SPINK4 PE=2 SV=1 - [ISK4_HUMAN]	1.00	1.00	5.09	5.71

▲	▲	Q06033	Inter-alpha-trypsin inhibitor heavy chain H3 OS=Homo sapiens GN=ITIH3 PE=1 SV=2 - [ITIH3_HUMAN]	8.00	8.00	2.90	5.70
▲	▲	P00740	Coagulation factor IX OS=Homo sapiens GN=F9 PE=1 SV=2 - [FA9_HUMAN]	6.00	6.00	2.18	5.68
▲	▲	P50120	Retinol-binding protein 2 OS=Homo sapiens GN=RBP2 PE=1 SV=3 - [RET2_HUMAN]	3.00	3.00	4.67	5.65
▲	▲	Q6NT04	Tigger transposable element-derived protein 7 OS=Homo sapiens GN=TIGD7 PE=2 SV=1 - [TIGD7_HUMAN]	1.00	1.00	5.37	5.65
▲	▲	P00739	Haptoglobin-related protein OS=Homo sapiens GN=HPR PE=1 SV=2 - [HPTR_HUMAN]	2.00	13.00	3.96	5.62
▲	▲	P20774	Mimecan OS=Homo sapiens GN=OGN PE=1 SV=1 - [MIME_HUMAN]	7.00	7.00	2.52	5.55
▲	▲	Q86V81	THO complex subunit 4 OS=Homo sapiens GN=THOC4 PE=1 SV=3 - [THOC4_HUMAN]	1.00	1.00	2.23	5.53
▲	▲	P04196	Histidine-rich glycoprotein OS=Homo sapiens GN=HRG PE=1 SV=1 - [HRG_HUMAN]	14.00	14.00	2.73	5.45
▲	▲	Q9BZ29	Dedicator of cytokinesis protein 9 OS=Homo sapiens GN=DOCK9 PE=1 SV=2 - [DOCK9_HUMAN]	1.00	1.00	4.24	5.45
▲	▲	Q14118	Dystroglycan OS=Homo sapiens GN=DAG1 PE=1 SV=2 - [DAG1_HUMAN]	9.00	9.00	2.21	5.43
▲	▲	P30042	ES1 protein homolog, mitochondrial OS=Homo sapiens GN=C21orf33 PE=1 SV=3 - [ES1_HUMAN]	3.00	3.00	2.02	5.40
▲	▲	P02753	Retinol-binding protein 4 OS=Homo sapiens GN=RBP4 PE=1 SV=3 - [RET4_HUMAN]	15.00	15.00	2.86	5.30
▲	▲	P08833	Insulin-like growth factor-binding protein 1 OS=Homo sapiens GN=IGFBP1 PE=1 SV=1 - [IBP1_HUMAN]	3.00	3.00	2.27	5.26
▲	▲	P24158	Myeloblastin OS=Homo sapiens GN=PRTN3 PE=1 SV=3 - [PRTN3_HUMAN]	4.00	4.00	2.54	5.21
▲	▲	P61769	Beta-2-microglobulin OS=Homo sapiens GN=B2M PE=1 SV=1 - [B2MG_HUMAN]	8.00	8.00	3.37	5.02
▲	▲	P08246	Neutrophil elastase OS=Homo sapiens GN=ELANE PE=1 SV=1 - [ELNE_HUMAN]	5.00	5.00	3.04	5.01
▲	▲	Q16610	Extracellular matrix protein 1 OS=Homo sapiens GN=ECM1 PE=1 SV=2 - [ECM1_HUMAN]	9.00	9.00	2.21	4.97
▲	▲	P80188	Neutrophil gelatinase-associated lipocalin OS=Homo sapiens GN=LCN2 PE=1 SV=2 - [NGAL_HUMAN]	9.00	9.00	3.31	4.96
▲	▲	Q8WW59	SPRY domain-containing protein 4 OS=Homo sapiens GN=SPRYD4 PE=1 SV=2 - [SPRY4_HUMAN]	1.00	1.00	2.97	4.94
▲	▲	P24592	Insulin-like growth factor-binding protein 6 OS=Homo sapiens GN=IGFBP6 PE=1 SV=1 - [IBP6_HUMAN]	7.00	7.00	2.15	4.93
▲	▲	P55103	Inhibin beta C chain OS=Homo sapiens GN=INHBC PE=1 SV=1 - [INHBC_HUMAN]	2.00	2.00	2.46	4.92
▲	▲	P06310	Ig kappa chain V-II region RPMI 6410 OS=Homo sapiens PE=4 SV=1 - [KV206_HUMAN]	1.00	3.00	15.95	4.85
▲	▲	P05164	Myeloperoxidase OS=Homo sapiens GN=MPO PE=1 SV=1 - [PERM_HUMAN]	20.00	20.00	2.43	4.84
▲	▲	Q5FWF4	Zinc finger Ran-binding domain-containing protein 3 OS=Homo sapiens GN=ZRANB3 PE=2 SV=2 - [ZRAB3_	1.00	1.00	3.46	4.81
▲	▲	P07988	Pulmonary surfactant-associated protein B OS=Homo sapiens GN=SFTPB PE=1 SV=3 - [PSPB_HUMAN]	2.00	2.00	2.09	4.80
▲	▲	P05546	Heparin cofactor 2 OS=Homo sapiens GN=SERPIND1 PE=1 SV=3 - [HEP2_HUMAN]	12.00	12.00	2.02	4.72
▲	▲	Q8N3J3	Uncharacterized protein C17orf53 OS=Homo sapiens GN=C17orf53 PE=2 SV=1 - [CQ053_HUMAN]	1.00	1.00	3.45	4.68
▲	▲	P07737	Profilin-1 OS=Homo sapiens GN=PFN1 PE=1 SV=2 - [PROF1_HUMAN]	8.00	8.00	2.29	4.67
▲	▲	Q01082	Spectrin beta chain, brain 1 OS=Homo sapiens GN=SPTBN1 PE=1 SV=2 - [SPTB2_HUMAN]	1.00	1.00	5.31	4.66
▲	▲	Q9BU40	Chordin-like protein 1 OS=Homo sapiens GN=CHRDL1 PE=2 SV=1 - [CRDL1_HUMAN]	2.00	2.00	2.06	4.66
▲	▲	P07478	Trypsin-2 OS=Homo sapiens GN=PRSS2 PE=1 SV=1 - [TRY2_HUMAN]	2.00	4.00	3.24	4.64
▲	▲	P02768	Serum albumin OS=Homo sapiens GN=ALB PE=1 SV=2 - [ALBU_HUMAN]	92.00	92.00	3.38	4.61
▲	▲	P01768	Ig heavy chain V-III region CAM OS=Homo sapiens PE=1 SV=1 - [HV307_HUMAN]	2.00	2.00	2.51	4.59
▲	▲	P08670	Vimentin OS=Homo sapiens GN=VIM PE=1 SV=4 - [VIME_HUMAN]	8.00	10.00	2.34	4.36
▲	▲	P06702	Protein S100-A9 OS=Homo sapiens GN=S100A9 PE=1 SV=1 - [S10A9_HUMAN]	7.00	7.00	2.05	4.35
▲	▲	Q13519	Nociceptin OS=Homo sapiens GN=PNOC PE=1 SV=1 - [PNOC_HUMAN]	1.00	1.00	2.85	4.30

▲	▲	P46939	Utrophin OS=Homo sapiens GN=UTRN PE=1 SV=2 - [UTRO_HUMAN]	1.00	1.00	2.17	4.13
▲	▲	P02790	Hemopexin OS=Homo sapiens GN=HPX PE=1 SV=2 - [HEMO_HUMAN]	25.00	25.00	2.30	4.12
▲	▲	A6NLU5	V-set and transmembrane domain-containing protein 2B OS=Homo sapiens GN=VSTM2B PE=2 SV=2 - [VTM	2.00	2.00	2.17	4.06
▲	▲	P01610	Ig kappa chain V-I region WEA OS=Homo sapiens PE=1 SV=1 - [KV118_HUMAN]	1.00	2.00	2.32	4.03
▲	▲	O43432	Eukaryotic translation initiation factor 4 gamma 3 OS=Homo sapiens GN=EIF4G3 PE=1 SV=2 - [IF4G3_HUM	1.00	1.00	2.92	4.01
▲	▲	Q6ZQW0	Indoleamine 2,3-dioxygenase 2 OS=Homo sapiens GN=IDO2 PE=1 SV=3 - [I23O2_HUMAN]	1.00	1.00	3.80	3.92
▲	▲	P09681	Gastric inhibitory polypeptide OS=Homo sapiens GN=GIP PE=1 SV=1 - [GIP_HUMAN]	4.00	4.00	2.25	3.91
▲	▲	P01023	Alpha-2-macroglobulin OS=Homo sapiens GN=A2M PE=1 SV=2 - [A2MG_HUMAN]	55.00	55.00	3.35	3.82
▲	▲	P00995	Pancreatic secretory trypsin inhibitor OS=Homo sapiens GN=SPINK1 PE=1 SV=2 - [ISK1_HUMAN]	3.00	3.00	2.37	3.81
▲	▲	Q9BWQ8	Fas apoptotic inhibitory molecule 2 OS=Homo sapiens GN=FAIM2 PE=1 SV=1 - [FAIM2_HUMAN]	1.00	1.00	2.16	3.74
▲	▲	Q9NZD4	Alpha-hemoglobin-stabilizing protein OS=Homo sapiens GN=AHSP PE=1 SV=1 - [AHSP_HUMAN]	3.00	3.00	2.43	3.74
▲	▲	Q9BZG9	Ly-6/neurotoxin-like protein 1 OS=Homo sapiens GN=LYNX1 PE=1 SV=2 - [LYNX1_HUMAN]	1.00	1.00	2.21	3.73
▲	▲	P01769	Ig heavy chain V-III region GA OS=Homo sapiens PE=1 SV=1 - [HV308_HUMAN]	2.00	2.00	2.42	3.73
▲	▲	O14793	Growth/differentiation factor 8 OS=Homo sapiens GN=MSTN PE=1 SV=1 - [GDF8_HUMAN]	2.00	2.00	2.02	3.64
▲	▲	P22894	Neutrophil collagenase OS=Homo sapiens GN=MMP8 PE=1 SV=1 - [MMP8_HUMAN]	5.00	5.00	3.45	3.58
▲	▲	P01773	Ig heavy chain V-III region BUR OS=Homo sapiens PE=1 SV=1 - [HV312_HUMAN]	1.00	1.00	2.01	3.55
▲	▲	O75368	SH3 domain-binding glutamic acid-rich-like protein OS=Homo sapiens GN=SH3BGRL PE=1 SV=1 - [SH3L1_	9.00	9.00	2.32	3.51
▲	▲	Q6JBY9	CapZ-interacting protein OS=Homo sapiens GN=RCSD1 PE=1 SV=1 - [CPZIP_HUMAN]	3.00	3.00	2.54	3.50
▲	▲	Q8TC20	Cancer-associated gene 1 protein OS=Homo sapiens GN=CAGE1 PE=2 SV=2 - [CAGE1_HUMAN]	1.00	1.00	2.52	3.50
▲	▲	P02787	Serotransferrin OS=Homo sapiens GN=TF PE=1 SV=2 - [TRFE_HUMAN]	58.00	58.00	2.64	3.49
▲	▲	O60353	Frizzled-6 OS=Homo sapiens GN=FZD6 PE=1 SV=2 - [FZD6_HUMAN]	1.00	1.00	3.02	3.49
▲	▲	Q7Z2H8	Proton-coupled amino acid transporter 1 OS=Homo sapiens GN=SLC36A1 PE=1 SV=1 - [S36A1_HUMAN]	1.00	1.00	2.13	3.45
▲	▲	P61956	Small ubiquitin-related modifier 2 OS=Homo sapiens GN=SUMO2 PE=1 SV=1 - [SUMO2_HUMAN]	1.00	1.00	2.00	3.41
▲	▲	Q92520	Protein FAM3C OS=Homo sapiens GN=FAM3C PE=1 SV=1 - [FAM3C_HUMAN]	11.00	11.00	2.23	3.35
▲	▲	O00264	Membrane-associated progesterone receptor component 1 OS=Homo sapiens GN=PGRMC1 PE=1 SV=3 - [PGR	1.00	2.00	2.17	3.30
▲	▲	Q86YW5	Trem-like transcript 1 protein OS=Homo sapiens GN=TREML1 PE=1 SV=2 - [TRML1_HUMAN]	2.00	2.00	3.37	3.29
▲	▲	P01011	Alpha-1-antichymotrypsin OS=Homo sapiens GN=SERPINA3 PE=1 SV=2 - [AACT_HUMAN]	24.00	24.00	2.27	3.27
▲	▲	Q15828	Cystatin-M OS=Homo sapiens GN=CST6 PE=1 SV=1 - [CYTM_HUMAN]	7.00	7.00	2.64	3.27
▲	▲	P43652	Afamin OS=Homo sapiens GN=AFM PE=1 SV=1 - [AFAM_HUMAN]	33.00	33.00	2.41	3.26
▲	▲	P29401	Transketolase OS=Homo sapiens GN=TKT PE=1 SV=3 - [TKT_HUMAN]	10.00	10.00	2.01	3.23
▲	▲	P02042	Hemoglobin subunit delta OS=Homo sapiens GN=HBD PE=1 SV=2 - [HBD_HUMAN]	5.00	10.00	2.78	3.18
▲	▲	P21817	Ryanodine receptor 1 OS=Homo sapiens GN=RYR1 PE=1 SV=3 - [RYR1_HUMAN]	1.00	1.00	2.77	3.17
▲	▲	Q9UHI8	A disintegrin and metalloproteinase with thrombospondin motifs 1 OS=Homo sapiens GN=ADAMTS1 PE=1 SV	3.00	3.00	2.50	3.17
▲	▲	P20160	Azurocidin OS=Homo sapiens GN=AZU1 PE=1 SV=3 - [CAP7_HUMAN]	3.00	3.00	2.07	3.17
▲	▲	P04114	Apolipoprotein B-100 OS=Homo sapiens GN=APOB PE=1 SV=1 - [APOB_HUMAN]	36.00	36.00	3.60	3.17
▲	▲	Q8NHQ9	ATP-dependent RNA helicase DDX55 OS=Homo sapiens GN=DDX55 PE=1 SV=3 - [DDX55_HUMAN]	1.00	1.00	2.55	3.16

▲	▲	Q99996	A-kinase anchor protein 9 OS=Homo sapiens GN=AKAP9 PE=1 SV=3 - [AKAP9_HUMAN]	1.00	1.00	4.16	3.15
▲	▲	Q6UWW0	Lipocalin-15 OS=Homo sapiens GN=LCN15 PE=2 SV=1 - [LCN15_HUMAN]	1.00	1.00	3.29	3.09
▲	▲	Q13231	Chitotriosidase-1 OS=Homo sapiens GN=CHIT1 PE=1 SV=1 - [CHIT1_HUMAN]	11.00	11.00	2.14	3.08
▲	▲	Q99674	Cell growth regulator with EF hand domain protein 1 OS=Homo sapiens GN=CGREF1 PE=2 SV=2 - [CGRE1_	5.00	5.00	2.03	3.07
▲	▲	P10645	Chromogranin-A OS=Homo sapiens GN=CHGA PE=1 SV=7 - [CMGA_HUMAN]	14.00	14.00	2.47	3.04
▲	▲	Q15363	Transmembrane emp24 domain-containing protein 2 OS=Homo sapiens GN=TMED2 PE=1 SV=1 - [TMED2_H	1.00	1.00	2.20	3.03
▲	▲	P51693	Amyloid-like protein 1 OS=Homo sapiens GN=APLP1 PE=1 SV=3 - [APLP1_HUMAN]	3.00	3.00	2.65	3.03
▲	▲	Q9UHB6	LIM domain and actin-binding protein 1 OS=Homo sapiens GN=LIMA1 PE=1 SV=1 - [LIMA1_HUMAN]	1.00	1.00	2.14	3.02
▲	▲	P08254	Stromelysin-1 OS=Homo sapiens GN=MMP3 PE=1 SV=2 - [MMP3_HUMAN]	1.00	1.00	2.40	2.96
▲	▲	Q9UF56	F-box/LRR-repeat protein 17 OS=Homo sapiens GN=FBXL17 PE=2 SV=3 - [FXL17_HUMAN]	1.00	1.00	2.54	2.96
▲	▲	P52799	Ephrin-B2 OS=Homo sapiens GN=EFNB2 PE=1 SV=1 - [EFNB2_HUMAN]	7.00	7.00	2.07	2.84
▲	▲	P12104	Fatty acid-binding protein, intestinal OS=Homo sapiens GN=FABP2 PE=1 SV=2 - [FABPI_HUMAN]	3.00	3.00	3.07	2.79
▲	▲	P02788	Lactotransferrin OS=Homo sapiens GN=LTF PE=1 SV=6 - [TRFL_HUMAN]	38.00	38.00	3.09	2.78
▲	▲	P15085	Carboxypeptidase A1 OS=Homo sapiens GN=CPA1 PE=1 SV=2 - [CBPA1_HUMAN]	7.00	7.00	2.31	2.73
▲	▲	P68871	Hemoglobin subunit beta OS=Homo sapiens GN=HBB PE=1 SV=2 - [HBB_HUMAN]	8.00	13.00	3.15	2.72
▲	▲	P06317	Ig lambda chain V-VI region SUT OS=Homo sapiens PE=1 SV=1 - [LV603_HUMAN]	1.00	1.00	2.44	2.72
▲	▲	Q14165	Malectin OS=Homo sapiens GN=MLEC PE=1 SV=1 - [MLEC_HUMAN]	4.00	4.00	2.02	2.70
▲	▲	P01714	Ig lambda chain V-III region SH OS=Homo sapiens PE=1 SV=1 - [LV301_HUMAN]	1.00	1.00	2.33	2.67
▲	▲	P01622	Ig kappa chain V-III region Ti OS=Homo sapiens PE=1 SV=1 - [KV304_HUMAN]	2.00	7.00	2.14	2.64
▲	▲	Q7Z4P5	Growth/differentiation factor 7 OS=Homo sapiens GN=GDF7 PE=2 SV=2 - [GDF7_HUMAN]	1.00	1.00	2.21	2.62
▲	▲	P69905	Hemoglobin subunit alpha OS=Homo sapiens GN=HBA1 PE=1 SV=2 - [HBA_HUMAN]	7.00	7.00	2.53	2.61
▲	▲	Q9H8J5	MANSC domain-containing protein 1 OS=Homo sapiens GN=MANSC1 PE=2 SV=1 - [MANS1_HUMAN]	4.00	4.00	2.32	2.58
▲	▲	Q86UP2	Kinectin OS=Homo sapiens GN=KTN1 PE=1 SV=1 - [KTN1_HUMAN]	1.00	1.00	2.14	2.57
▲	▲	P01602	Ig kappa chain V-I region HK102 (Fragment) OS=Homo sapiens GN=IGKV1-5 PE=4 SV=1 - [KV110_HUMA	2.00	2.00	2.65	2.53
▲	▲	Q6UY14	ADAMTS-like protein 4 OS=Homo sapiens GN=ADAMTSL4 PE=1 SV=2 - [ATL4_HUMAN]	7.00	7.00	2.33	2.53
▲	▲	Q15485	Ficolin-2 OS=Homo sapiens GN=FCN2 PE=1 SV=2 - [FCN2_HUMAN]	3.00	3.00	3.71	2.53
▲	▲	Q16661	Guanylate cyclase activator 2B OS=Homo sapiens GN=GUCA2B PE=1 SV=1 - [GUC2B_HUMAN]	4.00	4.00	2.77	2.50
▲	▲	Q8NBS9	Thioredoxin domain-containing protein 5 OS=Homo sapiens GN=TXNDC5 PE=1 SV=2 - [TXND5_HUMAN]	11.00	11.00	2.54	2.49
▲	▲	P00450	Ceruloplasmin OS=Homo sapiens GN=CP PE=1 SV=1 - [CERU_HUMAN]	43.00	43.00	2.14	2.47
▲	▲	P04217	Alpha-1B-glycoprotein OS=Homo sapiens GN=A1BG PE=1 SV=3 - [A1BG_HUMAN]	14.00	14.00	2.31	2.41
▲	▲	P57087	Junctional adhesion molecule B OS=Homo sapiens GN=JAM2 PE=1 SV=1 - [JAM2_HUMAN]	1.00	1.00	2.20	2.37
▲	▲	Q6UWJ1	Transmembrane and coiled-coil domain-containing protein 3 OS=Homo sapiens GN=TMCO3 PE=2 SV=1 - [TM	1.00	1.00	2.14	2.35
▲	▲	P01781	Ig heavy chain V-III region GAL OS=Homo sapiens PE=1 SV=1 - [HV320_HUMAN]	3.00	3.00	2.09	2.33
▲	▲	P14780	Matrix metalloproteinase-9 OS=Homo sapiens GN=MMP9 PE=1 SV=3 - [MMP9_HUMAN]	16.00	16.00	2.05	2.32
▲	▲	Q02747	Guanylin OS=Homo sapiens GN=GUCA2A PE=1 SV=2 - [GUC2A_HUMAN]	4.00	4.00	3.13	2.32
▲	▲	P52848	Bifunctional heparan sulfate N-deacetylase/N-sulfotransferase 1 OS=Homo sapiens GN=NDST1 PE=1 SV=1 - [	1.00	1.00	2.60	2.31

▲	▲	Q86YD5	Low-density lipoprotein receptor class A domain-containing protein 3 OS=Homo sapiens GN=LDLRAD3 PE=2	1.00	1.00	2.44	2.31
▲	▲	P01767	Ig heavy chain V-III region BUT OS=Homo sapiens PE=1 SV=1 - [HV306_HUMAN]	2.00	3.00	2.25	2.30
▲	▲	O95711	Lymphocyte antigen 86 OS=Homo sapiens GN=LY86 PE=1 SV=1 - [LY86_HUMAN]	1.00	1.00	2.39	2.24
▲	▲	Q6P3W7	SCY1-like protein 2 OS=Homo sapiens GN=SCYL2 PE=1 SV=1 - [SCYL2_HUMAN]	1.00	1.00	2.25	2.22
▲	▲	Q2NL68	Uncharacterized protein C19orf55 OS=Homo sapiens GN=C19orf55 PE=2 SV=1 - [CS055_HUMAN]	1.00	1.00	3.01	2.18
▲	▲	Q9NQ55	Suppressor of SWI4 1 homolog OS=Homo sapiens GN=PPAN PE=1 SV=1 - [SSF1_HUMAN]	1.00	1.00	2.15	2.13
▲	▲	Q9H6B4	Adipocyte adhesion molecule OS=Homo sapiens GN=ACAM PE=1 SV=1 - [ACAM_HUMAN]	3.00	3.00	2.57	2.13
▲	▲	O43405	Cochlin OS=Homo sapiens GN=COCH PE=1 SV=1 - [COCH_HUMAN]	2.00	2.00	2.51	2.08
▲	▲	Q9NWV4	UPF0587 protein C1orf123 OS=Homo sapiens GN=C1orf123 PE=1 SV=1 - [CA123_HUMAN]	3.00	3.00	2.01	2.05
▲	▲	P01766	Ig heavy chain V-III region BRO OS=Homo sapiens PE=1 SV=1 - [HV305_HUMAN]	1.00	2.00	2.11	2.03
▲	▲	P01765	Ig heavy chain V-III region TIL OS=Homo sapiens PE=1 SV=1 - [HV304_HUMAN]	1.00	2.00	2.27	2.01
▲	▲	O95502	Neuronal pentraxin receptor OS=Homo sapiens GN=NPTXR PE=2 SV=2 - [NPTXR_HUMAN]	1.00	1.00	2.17	2.00
▲	▲	P01876	Ig alpha-1 chain C region OS=Homo sapiens GN=IGHA1 PE=1 SV=2 - [IGHA1_HUMAN]	7.00	16.00	2.09	2.00
		**Up-Regulated Proteins in any one Subgroups (400)**					
▲		Q6N063	2-oxoglutarate and iron-dependent oxygenase domain-containing protein 2 OS=Homo sapiens GN=OGFOD2 PE	1.00	1.00	3.03	1.98
▲		P00915	Carbonic anhydrase 1 OS=Homo sapiens GN=CA1 PE=1 SV=2 - [CAH1_HUMAN]	14.00	14.00	2.20	1.97
▲		P01275	Glucagon OS=Homo sapiens GN=GCG PE=1 SV=3 - [GLUC_HUMAN]	1.00	1.00	4.15	1.94
▲		P04216	Thy-1 membrane glycoprotein OS=Homo sapiens GN=THY1 PE=1 SV=2 - [THY1_HUMAN]	4.00	4.00	2.25	1.93
▲		Q9UBR2	Cathepsin Z OS=Homo sapiens GN=CTSZ PE=1 SV=1 - [CATZ_HUMAN]	8.00	8.00	2.12	1.91
▲		P00736	Complement C1r subcomponent OS=Homo sapiens GN=C1R PE=1 SV=2 - [C1R_HUMAN]	3.00	4.00	2.94	1.83
▲		P69891	Hemoglobin subunit gamma-1 OS=Homo sapiens GN=HBG1 PE=1 SV=2 - [HBG1_HUMAN]	1.00	2.00	2.20	1.83
▲		P05451	Lithostathine-1-alpha OS=Homo sapiens GN=REG1A PE=1 SV=3 - [REG1A_HUMAN]	7.00	11.00	2.64	1.71
▲		P01611	Ig kappa chain V-I region Wes OS=Homo sapiens PE=1 SV=1 - [KV119_HUMAN]	1.00	1.00	2.01	1.70
▲		P01601	Ig kappa chain V-I region HK101 (Fragment) OS=Homo sapiens PE=4 SV=1 - [KV109_HUMAN]	1.00	1.00	2.33	1.68
▲		P12110	Collagen alpha-2(VI) chain OS=Homo sapiens GN=COL6A2 PE=1 SV=4 - [CO6A2_HUMAN]	5.00	5.00	2.00	1.63
▲		P02763	Alpha-1-acid glycoprotein 1 OS=Homo sapiens GN=ORM1 PE=1 SV=1 - [A1AG1_HUMAN]	12.00	20.00	2.27	1.57
▲		P15018	Leukemia inhibitory factor OS=Homo sapiens GN=LIF PE=1 SV=1 - [LIF_HUMAN]	1.00	1.00	3.10	1.53
▲		P06314	Ig kappa chain V-IV region B17 OS=Homo sapiens PE=2 SV=1 - [KV404_HUMAN]	1.00	6.00	2.82	1.50
▲		A6NI79	Coiled-coil domain-containing protein 69 OS=Homo sapiens GN=CCDC69 PE=2 SV=1 - [CCD69_HUMAN]	1.00	1.00	2.66	1.47
▲		Q8NI35	InaD-like protein OS=Homo sapiens GN=INADL PE=1 SV=3 - [INADL_HUMAN]	1.00	1.00	2.10	1.44
▲		Q8TDL5	Long palate, lung and nasal epithelium carcinoma-associated protein 1 OS=Homo sapiens GN=LPLUNC1 PE=2	2.00	2.00	2.11	1.44
▲		P04211	Ig lambda chain V region 4A OS=Homo sapiens PE=4 SV=1 - [LV001_HUMAN]	1.00	1.00	2.12	1.44
▲		Q9Y2I9	TBC1 domain family member 30 OS=Homo sapiens GN=TBC1D30 PE=2 SV=2 - [TBC30_HUMAN]	1.00	1.00	3.01	1.41
▲		Q15699	ALX homeobox protein 1 OS=Homo sapiens GN=ALX1 PE=1 SV=2 - [ALX1_HUMAN]	1.00	1.00	2.31	1.39
▲		O60635	Tetraspanin-1 OS=Homo sapiens GN=TSPAN1 PE=1 SV=2 - [TSN1_HUMAN]	2.00	2.00	4.71	1.35
▲		P40926	Malate dehydrogenase, mitochondrial OS=Homo sapiens GN=MDH2 PE=1 SV=3 - [MDHM_HUMAN]	4.00	4.00	2.18	1.32

▲		P03973	Antileukoproteinase OS=Homo sapiens GN=SLPI PE=1 SV=2 - [SLPI_HUMAN]	5.00	5.00	2.09	1.31
▲		P01709	Ig lambda chain V-II region MGC OS=Homo sapiens PE=1 SV=1 - [LV206_HUMAN]	1.00	2.00	2.15	1.28
▲		P42765	3-ketoacyl-CoA thiolase, mitochondrial OS=Homo sapiens GN=ACAA2 PE=1 SV=2 - [THIM_HUMAN]	2.00	2.00	2.25	1.28
▲		Q02383	Semenogelin-2 OS=Homo sapiens GN=SEMG2 PE=1 SV=1 - [SEMG2_HUMAN]	8.00	9.00	10.60	1.25
▲		Q8WVN6	Secreted and transmembrane protein 1 OS=Homo sapiens GN=SECTM1 PE=1 SV=2 - [SCTM1_HUMAN]	4.00	4.00	2.18	1.22
▲		P30084	Enoyl-CoA hydratase, mitochondrial OS=Homo sapiens GN=ECHS1 PE=1 SV=4 - [ECHM_HUMAN]	2.00	2.00	2.21	1.11
▲		Q9Y3B3	Transmembrane emp24 domain-containing protein 7 OS=Homo sapiens GN=TMED7 PE=1 SV=2 - [TMED7_H	1.00	1.00	2.37	1.07
▲		Q86V85	Integral membrane protein GPR180 OS=Homo sapiens GN=GPR180 PE=2 SV=1 - [GP180_HUMAN]	2.00	2.00	2.65	1.04
▲		Q5UCC4	UPF0510 protein INM02 OS=Homo sapiens GN=C19orf63 PE=1 SV=1 - [INM02_HUMAN]	1.00	1.00	2.06	1.01
▲		Q9ULV1	Frizzled-4 OS=Homo sapiens GN=FZD4 PE=1 SV=2 - [FZD4_HUMAN]	1.00	1.00	2.25	0.99
▲		P04279	Semenogelin-1 OS=Homo sapiens GN=SEMG1 PE=1 SV=2 - [SEMG1_HUMAN]	5.00	6.00	20.55	0.92
▲		Q96M29	Tektin-5 OS=Homo sapiens GN=TEKT5 PE=2 SV=1 - [TEKT5_HUMAN]	1.00	1.00	2.54	0.82
▲		P49788	Retinoic acid receptor responder protein 1 OS=Homo sapiens GN=RARRES1 PE=2 SV=2 - [TIG1_HUMAN]	1.00	1.00	2.60	0.81
▲		P06576	ATP synthase subunit beta, mitochondrial OS=Homo sapiens GN=ATP5B PE=1 SV=3 - [ATPB_HUMAN]	1.00	1.00	2.01	0.74
▲		Q02252	Methylmalonate-semialdehyde dehydrogenase [acylating], mitochondrial OS=Homo sapiens GN=ALDH6A1 PE	1.00	1.00	3.46	0.71
▲		Q9H227	Cytosolic beta-glucosidase OS=Homo sapiens GN=GBA3 PE=1 SV=2 - [GBA3_HUMAN]	1.00	1.00	7.48	0.60
▲		P55083	Microfibril-associated glycoprotein 4 OS=Homo sapiens GN=MFAP4 PE=1 SV=2 - [MFAP4_HUMAN]	1.00	1.00	2.52	0.52
	▲	P62857	40S ribosomal protein S28 OS=Homo sapiens GN=RPS28 PE=1 SV=1 - [RS28_HUMAN]	2.00	2.00	1.80	29.83
	▲	P02656	Apolipoprotein C-III OS=Homo sapiens GN=APOC3 PE=1 SV=1 - [APOC3_HUMAN]	4.00	4.00	1.67	15.13
	▲	P30049	ATP synthase subunit delta, mitochondrial OS=Homo sapiens GN=ATP5D PE=1 SV=2 - [ATPD_HUMAN]	1.00	1.00	1.23	13.21
	▲	P08493	Matrix Gla protein OS=Homo sapiens GN=MGP PE=1 SV=2 - [MGP_HUMAN]	1.00	1.00	1.44	13.18
	▲	P01160	Atrial natriuretic factor OS=Homo sapiens GN=NPPA PE=1 SV=1 - [ANF_HUMAN]	1.00	1.00	1.79	13.15
	▲	P07951	Tropomyosin beta chain OS=Homo sapiens GN=TPM2 PE=1 SV=1 - [TPM2_HUMAN]	2.00	7.00	1.89	13.03
	▲	O75208	Ubiquinone biosynthesis protein COQ9, mitochondrial OS=Homo sapiens GN=COQ9 PE=1 SV=1 - [COQ9_HU	1.00	1.00		11.88
	▲	Q01995	Transgelin OS=Homo sapiens GN=TAGLN PE=1 SV=4 - [TAGL_HUMAN]	9.00	9.00	1.72	11.76
	▲	P36578	60S ribosomal protein L4 OS=Homo sapiens GN=RPL4 PE=1 SV=5 - [RL4_HUMAN]	1.00	1.00	1.48	11.75
	▲	P20962	Parathymosin OS=Homo sapiens GN=PTMS PE=1 SV=2 - [PTMS_HUMAN]	3.00	3.00	1.16	11.70
	▲	P31327	Carbamoyl-phosphate synthase [ammonia], mitochondrial OS=Homo sapiens GN=CPS1 PE=1 SV=2 - [CPSM_	1.00	1.00	1.03	11.07
	▲	O75381	Peroxisomal membrane protein PEX14 OS=Homo sapiens GN=PEX14 PE=1 SV=1 - [PEX14_HUMAN]	1.00	1.00	1.66	10.63
	▲	Q8WUJ0	Serine/threonine/tyrosine-interacting protein OS=Homo sapiens GN=STYX PE=1 SV=1 - [STYX_HUMAN]	1.00	1.00	1.91	9.84
	▲	P51460	Insulin-like 3 OS=Homo sapiens GN=INSL3 PE=1 SV=1 - [INSL3_HUMAN]	1.00	1.00	1.41	9.09
	▲	P07108	Acyl-CoA-binding protein OS=Homo sapiens GN=DBI PE=1 SV=2 - [ACBP_HUMAN]	5.00	5.00	1.42	9.01
	▲	P07148	Fatty acid-binding protein, liver OS=Homo sapiens GN=FABP1 PE=1 SV=1 - [FABPL_HUMAN]	9.00	9.00	1.59	8.14
	▲	P18428	Lipopolysaccharide-binding protein OS=Homo sapiens GN=LBP PE=1 SV=3 - [LBP_HUMAN]	8.00	8.00	1.73	8.14
	▲	Q8NBP7	Proprotein convertase subtilisin/kexin type 9 OS=Homo sapiens GN=PCSK9 PE=1 SV=2 - [PCSK9_HUMAN]	1.00	1.00	1.68	7.86
	▲	Q66K74	Microtubule-associated protein 1S OS=Homo sapiens GN=MAP1S PE=1 SV=2 - [MAP1S_HUMAN]	1.00	1.00	1.70	7.75

	▲	Q16595	Frataxin, mitochondrial OS=Homo sapiens GN=FXN PE=1 SV=2 - [FRDA_HUMAN]	2.00	2.00	1.29	7.74
	▲	P01185	Vasopressin-neurophysin 2-copeptin OS=Homo sapiens GN=AVP PE=1 SV=2 - [NEU2_HUMAN]	1.00	2.00	1.46	7.70
	▲	P22692	Insulin-like growth factor-binding protein 4 OS=Homo sapiens GN=IGFBP4 PE=1 SV=2 - [IBP4_HUMAN]	8.00	8.00	1.92	7.66
	▲	Q05315	Eosinophil lysophospholipase OS=Homo sapiens GN=CLC PE=1 SV=2 - [LPPL_HUMAN]	1.00	1.00	1.78	7.65
	▲	P39019	40S ribosomal protein S19 OS=Homo sapiens GN=RPS19 PE=1 SV=2 - [RS19_HUMAN]	1.00	1.00	1.01	7.62
	▲	P23434	Glycine cleavage system H protein, mitochondrial OS=Homo sapiens GN=GCSH PE=1 SV=1 - [GCSH_HUMA	2.00	2.00	1.27	7.43
	▲	O95456	Proteasome assembly chaperone 1 OS=Homo sapiens GN=PSMG1 PE=1 SV=1 - [PSMG1_HUMAN]	1.00	1.00	1.21	7.18
	▲	Q14520	Hyaluronan-binding protein 2 OS=Homo sapiens GN=HABP2 PE=1 SV=1 - [HABP2_HUMAN]	4.00	4.00	1.94	7.13
	▲	O00515	Ladinin-1 OS=Homo sapiens GN=LAD1 PE=1 SV=2 - [LAD1_HUMAN]	2.00	2.00	1.52	6.99
	▲	Q99541	Perilipin-2 OS=Homo sapiens GN=PLIN2 PE=1 SV=2 - [PLIN2_HUMAN]	1.00	1.00	1.37	6.93
	▲	P10644	cAMP-dependent protein kinase type I-alpha regulatory subunit OS=Homo sapiens GN=PRKAR1A PE=1 SV=1	2.00	2.00	1.80	6.85
	▲	P01880	Ig delta chain C region OS=Homo sapiens GN=IGHD PE=1 SV=2 - [IGHD_HUMAN]	5.00	5.00	1.77	6.79
	▲	Q16663	C-C motif chemokine 15 OS=Homo sapiens GN=CCL15 PE=1 SV=2 - [CCL15_HUMAN]	1.00	1.00	1.44	6.77
	▲	Q93091	Ribonuclease K6 OS=Homo sapiens GN=RNASE6 PE=1 SV=2 - [RNAS6_HUMAN]	1.00	1.00	1.81	6.73
	▲	P61960	Ubiquitin-fold modifier 1 OS=Homo sapiens GN=UFM1 PE=1 SV=1 - [UFM1_HUMAN]	3.00	3.00	1.79	6.72
	▲	P26885	Peptidyl-prolyl cis-trans isomerase FKBP2 OS=Homo sapiens GN=FKBP2 PE=1 SV=2 - [FKBP2_HUMAN]	2.00	2.00	1.99	6.71
	▲	P34096	Ribonuclease 4 OS=Homo sapiens GN=RNASE4 PE=1 SV=3 - [RNAS4_HUMAN]	4.00	4.00	1.42	6.53
	▲	P41159	Leptin OS=Homo sapiens GN=LEP PE=1 SV=1 - [LEP_HUMAN]	3.00	3.00	1.75	6.47
	▲	Q16762	Thiosulfate sulfurtransferase OS=Homo sapiens GN=TST PE=1 SV=4 - [THTR_HUMAN]	3.00	3.00	1.69	6.36
	▲	Q96NZ9	Proline-rich acidic protein 1 OS=Homo sapiens GN=PRAP1 PE=2 SV=1 - [PRAP1_HUMAN]	5.00	5.00	1.68	6.25
	▲	P46952	3-hydroxyanthranilate 3,4-dioxygenase OS=Homo sapiens GN=HAAO PE=1 SV=1 - [3HAO_HUMAN]	9.00	9.00	1.39	6.07
	▲	P06681	Complement C2 OS=Homo sapiens GN=C2 PE=1 SV=2 - [CO2_HUMAN]	15.00	15.00	1.90	5.97
	▲	Q13790	Apolipoprotein F OS=Homo sapiens GN=APOF PE=1 SV=1 - [APOF_HUMAN]	4.00	4.00	1.92	5.92
	▲	P00441	Superoxide dismutase [Cu-Zn] OS=Homo sapiens GN=SOD1 PE=1 SV=2 - [SODC_HUMAN]	12.00	12.00	1.77	5.91
	▲	P07360	Complement component C8 gamma chain OS=Homo sapiens GN=C8G PE=1 SV=3 - [CO8G_HUMAN]	9.00	9.00	1.98	5.90
	▲	P02735	Serum amyloid A protein OS=Homo sapiens GN=SAA1 PE=1 SV=2 - [SAA_HUMAN]	6.00	6.00	1.60	5.89
	▲	P04732	Metallothionein-1E OS=Homo sapiens GN=MT1E PE=1 SV=1 - [MT1E_HUMAN]	1.00	2.00	1.49	5.88
	▲	P62750	60S ribosomal protein L23a OS=Homo sapiens GN=RPL23A PE=1 SV=1 - [RL23A_HUMAN]	1.00	1.00	0.76	5.87
	▲	Q9NQ76	Matrix extracellular phosphoglycoprotein OS=Homo sapiens GN=MEPE PE=1 SV=1 - [MEPE_HUMAN]	3.00	3.00	1.67	5.87
	▲	P02655	Apolipoprotein C-II OS=Homo sapiens GN=APOC2 PE=1 SV=1 - [APOC2_HUMAN]	4.00	4.00	1.93	5.86
	▲	Q49AM3	Tetratricopeptide repeat protein 31 OS=Homo sapiens GN=TTC31 PE=2 SV=3 - [TTC31_HUMAN]	1.00	1.00	1.84	5.77
	▲	P80297	Metallothionein-1X OS=Homo sapiens GN=MT1X PE=1 SV=1 - [MT1X_HUMAN]	2.00	3.00	1.26	5.77
	▲	P07357	Complement component C8 alpha chain OS=Homo sapiens GN=C8A PE=1 SV=2 - [CO8A_HUMAN]	10.00	10.00	1.86	5.71
	▲	P30046	D-dopachrome decarboxylase OS=Homo sapiens GN=DDT PE=1 SV=3 - [DOPD_HUMAN]	4.00	4.00	1.13	5.69
	▲	Q99757	Thioredoxin, mitochondrial OS=Homo sapiens GN=TXN2 PE=1 SV=2 - [THIOM_HUMAN]	3.00	3.00	1.70	5.66
	▲	P20810	Calpastatin OS=Homo sapiens GN=CAST PE=1 SV=4 - [ICAL_HUMAN]	3.00	3.00	1.82	5.64

	▲	P36222	Chitinase-3-like protein 1 OS=Homo sapiens GN=CHI3L1 PE=1 SV=2 - [CH3L1_HUMAN]	10.00	10.00	1.54	5.61
	▲	P02654	Apolipoprotein C-I OS=Homo sapiens GN=APOC1 PE=1 SV=1 - [APOC1_HUMAN]	4.00	4.00	1.80	5.53
	▲	Q9NYJ1	Coiled-coil-helix-coiled-coil-helix domain-containing protein 8 OS=Homo sapiens GN=CHCHD8 PE=1 SV=2 -	1.00	1.00	1.39	5.51
	▲	Q15493	Regucalcin OS=Homo sapiens GN=RGN PE=1 SV=1 - [RGN_HUMAN]	7.00	7.00	0.73	5.48
	▲	Q02878	60S ribosomal protein L6 OS=Homo sapiens GN=RPL6 PE=1 SV=3 - [RL6_HUMAN]	1.00	1.00	1.43	5.38
	▲	P20848	Putative alpha-1-antitrypsin-related protein OS=Homo sapiens GN=SERPINA2 PE=5 SV=1 - [A1ATR_HUMA	1.00	2.00	1.56	5.35
	▲	P15502	Elastin OS=Homo sapiens GN=ELN PE=1 SV=2 - [ELN_HUMAN]	2.00	2.00	1.63	5.30
	▲	Q03591	Complement factor H-related protein 1 OS=Homo sapiens GN=CFHR1 PE=1 SV=2 - [FHR1_HUMAN]	2.00	8.00	1.92	5.29
	▲	Q06141	Regenerating islet-derived protein 3-alpha OS=Homo sapiens GN=REG3A PE=1 SV=1 - [REG3A_HUMAN]	3.00	3.00	1.90	5.15
	▲	Q9HCB6	Spondin-1 OS=Homo sapiens GN=SPON1 PE=1 SV=2 - [SPON1_HUMAN]	2.00	2.00	1.37	5.05
	▲	P10109	Adrenodoxin, mitochondrial OS=Homo sapiens GN=FDX1 PE=1 SV=1 - [ADX_HUMAN]	4.00	4.00	0.75	5.05
	▲	P00746	Complement factor D OS=Homo sapiens GN=CFD PE=1 SV=5 - [CFAD_HUMAN]	12.00	12.00	1.92	5.04
	▲	P01009	Alpha-1-antitrypsin OS=Homo sapiens GN=SERPINA1 PE=1 SV=3 - [A1AT_HUMAN]	34.00	35.00	1.66	5.03
	▲	P61923	Coatomer subunit zeta-1 OS=Homo sapiens GN=COPZ1 PE=1 SV=1 - [COPZ1_HUMAN]	1.00	1.00	1.22	5.02
	▲	O95810	Serum deprivation-response protein OS=Homo sapiens GN=SDPR PE=1 SV=3 - [SDPR_HUMAN]	2.00	2.00	1.79	5.02
	▲	O15204	ADAM DEC1 OS=Homo sapiens GN=ADAMDEC1 PE=1 SV=2 - [ADEC1_HUMAN]	2.00	2.00	1.48	4.99
	▲	Q12874	Splicing factor 3A subunit 3 OS=Homo sapiens GN=SF3A3 PE=1 SV=1 - [SF3A3_HUMAN]	1.00	1.00	1.33	4.96
	▲	P01860	Ig gamma-3 chain C region OS=Homo sapiens GN=IGHG3 PE=1 SV=2 - [IGHG3_HUMAN]	7.00	17.00	1.90	4.92
	▲	P07358	Complement component C8 beta chain OS=Homo sapiens GN=C8B PE=1 SV=3 - [CO8B_HUMAN]	6.00	6.00	1.49	4.91
	▲	P11686	Pulmonary surfactant-associated protein C OS=Homo sapiens GN=SFTPC PE=1 SV=2 - [PSPC_HUMAN]	1.00	1.00	1.04	4.91
	▲	Q9UNH7	Sorting nexin-6 OS=Homo sapiens GN=SNX6 PE=1 SV=1 - [SNX6_HUMAN]	1.00	1.00	1.28	4.86
	▲	P09382	Galectin-1 OS=Homo sapiens GN=LGALS1 PE=1 SV=2 - [LEG1_HUMAN]	3.00	3.00	1.65	4.85
	▲	P04278	Sex hormone-binding globulin OS=Homo sapiens GN=SHBG PE=1 SV=2 - [SHBG_HUMAN]	8.00	8.00	1.17	4.84
	▲	P56277	Protein p8 MTCP-1 OS=Homo sapiens GN=MTCP1 PE=1 SV=1 - [MTCPA_HUMAN]	1.00	1.00	1.25	4.83
	▲	P12882	Myosin-1 OS=Homo sapiens GN=MYH1 PE=1 SV=3 - [MYH1_HUMAN]	2.00	9.00	1.89	4.78
	▲	P08697	Alpha-2-antiplasmin OS=Homo sapiens GN=SERPINF2 PE=1 SV=3 - [A2AP_HUMAN]	17.00	17.00	1.98	4.73
	▲	P62942	Peptidyl-prolyl cis-trans isomerase FKBP1A OS=Homo sapiens GN=FKBP1A PE=1 SV=2 - [FKB1A_HUMAN	3.00	3.00	1.38	4.67
	▲	Q15084	Protein disulfide-isomerase A6 OS=Homo sapiens GN=PDIA6 PE=1 SV=1 - [PDIA6_HUMAN]	2.00	2.00	1.42	4.66
	▲	P02741	C-reactive protein OS=Homo sapiens GN=CRP PE=1 SV=1 - [CRP_HUMAN]	7.00	7.00	1.52	4.64
	▲	P08603	Complement factor H OS=Homo sapiens GN=CFH PE=1 SV=4 - [CFAH_HUMAN]	21.00	23.00	2.00	4.63
	▲	P19823	Inter-alpha-trypsin inhibitor heavy chain H2 OS=Homo sapiens GN=ITIH2 PE=1 SV=2 - [ITIH2_HUMAN]	19.00	19.00	1.91	4.62
	▲	P17661	Desmin OS=Homo sapiens GN=DES PE=1 SV=3 - [DESM_HUMAN]	2.00	4.00	1.38	4.61
	▲	P41271	Neuroblastoma suppressor of tumorigenicity 1 OS=Homo sapiens GN=NBL1 PE=1 SV=1 - [NBL1_HUMAN]	2.00	2.00	1.55	4.58
	▲	Q13907	Isopentenyl-diphosphate Delta-isomerase 1 OS=Homo sapiens GN=IDI1 PE=1 SV=2 - [IDI1_HUMAN]	1.00	1.00	1.52	4.58
	▲	Q9Y2B0	Protein canopy homolog 2 OS=Homo sapiens GN=CNPY2 PE=1 SV=1 - [CNPY2_HUMAN]	1.00	1.00	1.69	4.55
	▲	Q9H6Z4	Ran-binding protein 3 OS=Homo sapiens GN=RANBP3 PE=1 SV=1 - [RANB3_HUMAN]	1.00	1.00	1.94	4.55

	▲	Q9UJC5	SH3 domain-binding glutamic acid-rich-like protein 2 OS=Homo sapiens GN=SH3BGRL2 PE=1 SV=2 - [SH3L	1.00	1.00	1.46	4.55
	▲	P03952	Plasma kallikrein OS=Homo sapiens GN=KLKB1 PE=1 SV=1 - [KLKB1_HUMAN]	10.00	10.00	1.96	4.55
	▲	Q969H8	UPF0556 protein C19orf10 OS=Homo sapiens GN=C19orf10 PE=1 SV=1 - [CS010_HUMAN]	6.00	6.00	1.90	4.53
	▲	P35318	ADM OS=Homo sapiens GN=ADM PE=1 SV=1 - [ADML_HUMAN]	2.00	2.00	1.38	4.45
	▲	P82979	SAP domain-containing ribonucleoprotein OS=Homo sapiens GN=SARNP PE=1 SV=3 - [SARNP_HUMAN]	1.00	1.00	1.70	4.43
	▲	Q5SSJ5	Heterochromatin protein 1-binding protein 3 OS=Homo sapiens GN=HP1BP3 PE=1 SV=1 - [HP1B3_HUMAN]	1.00	1.00	0.70	4.41
	▲	P27797	Calreticulin OS=Homo sapiens GN=CALR PE=1 SV=1 - [CALR_HUMAN]	15.00	15.00	1.54	4.37
	▲	Q07065	Cytoskeleton-associated protein 4 OS=Homo sapiens GN=CKAP4 PE=1 SV=2 - [CKAP4_HUMAN]	1.00	1.00	0.96	4.34
	▲	Q17RC7	SEC6-like protein C14orf73 OS=Homo sapiens GN=C14orf73 PE=2 SV=2 - [CN073_HUMAN]	1.00	1.00	1.10	4.31
	▲	Q9H3N1	Thioredoxin-related transmembrane protein 1 OS=Homo sapiens GN=TMX1 PE=1 SV=1 - [TMX1_HUMAN]	1.00	1.00	1.07	4.31
	▲	Q9BUD6	Spondin-2 OS=Homo sapiens GN=SPON2 PE=1 SV=2 - [SPON2_HUMAN]	4.00	4.00	1.52	4.30
	▲	Q13444	Disintegrin and metalloproteinase domain-containing protein 15 OS=Homo sapiens GN=ADAM15 PE=1 SV=3	2.00	2.00	1.01	4.28
	▲	P80511	Protein S100-A12 OS=Homo sapiens GN=S100A12 PE=1 SV=2 - [S10AC_HUMAN]	3.00	3.00	1.86	4.27
	▲	Q96A32	Myosin regulatory light chain 2, skeletal muscle isoform OS=Homo sapiens GN=MYLPF PE=2 SV=1 - [MLRS_	1.00	1.00	1.38	4.26
	▲	Q9UMX5	Neudesin OS=Homo sapiens GN=NENF PE=1 SV=1 - [NENF_HUMAN]	7.00	7.00	1.70	4.17
	▲	Q9Y5Z4	Heme-binding protein 2 OS=Homo sapiens GN=HEBP2 PE=1 SV=1 - [HEBP2_HUMAN]	6.00	6.00	1.48	4.17
	▲	P00747	Plasminogen OS=Homo sapiens GN=PLG PE=1 SV=2 - [PLMN_HUMAN]	35.00	35.00	1.94	4.14
	▲	P01814	Ig heavy chain V-II region OU OS=Homo sapiens PE=1 SV=1 - [HV201_HUMAN]	1.00	1.00	1.90	4.14
	▲	Q96Q06	Perilipin-4 OS=Homo sapiens GN=PLIN4 PE=2 SV=2 - [PLIN4_HUMAN]	2.00	2.00	1.78	4.12
	▲	Q12983	BCL2/adenovirus E1B 19 kDa protein-interacting protein 3 OS=Homo sapiens GN=BNIP3 PE=1 SV=2 - [BNIP	1.00	1.00	1.32	4.10
	▲	Q9BRA2	Thioredoxin domain-containing protein 17 OS=Homo sapiens GN=TXNDC17 PE=1 SV=1 - [TXD17_HUMAN	2.00	2.00	1.38	4.05
	▲	Q05682	Caldesmon OS=Homo sapiens GN=CALD1 PE=1 SV=2 - [CALD1_HUMAN]	5.00	5.00	1.32	4.03
	▲	O60240	Perilipin-1 OS=Homo sapiens GN=PLIN1 PE=2 SV=2 - [PLIN1_HUMAN]	2.00	2.00	0.91	3.98
	▲	P23588	Eukaryotic translation initiation factor 4B OS=Homo sapiens GN=EIF4B PE=1 SV=2 - [IF4B_HUMAN]	2.00	2.00	1.24	3.95
	▲	P05109	Protein S100-A8 OS=Homo sapiens GN=S100A8 PE=1 SV=1 - [S10A8_HUMAN]	9.00	9.00	1.93	3.92
	▲	P06732	Creatine kinase M-type OS=Homo sapiens GN=CKM PE=1 SV=2 - [KCRM_HUMAN]	2.00	2.00	1.23	3.90
	▲	P13497	Bone morphogenetic protein 1 OS=Homo sapiens GN=BMP1 PE=1 SV=2 - [BMP1_HUMAN]	2.00	2.00	1.44	3.90
	▲	Q9BXX0	EMILIN-2 OS=Homo sapiens GN=EMILIN2 PE=1 SV=3 - [EMIL2_HUMAN]	5.00	5.00	1.89	3.89
	▲	Q8WWX9	Selenoprotein M OS=Homo sapiens GN=SELM PE=1 SV=3 - [SELM_HUMAN]	2.00	2.00	1.42	3.88
	▲	Q9UBE0	SUMO-activating enzyme subunit 1 OS=Homo sapiens GN=SAE1 PE=1 SV=1 - [SAE1_HUMAN]	1.00	1.00	1.29	3.87
	▲	Q8NHH1	Tubulin polyglutamylase TTLL11 OS=Homo sapiens GN=TTLL11 PE=2 SV=1 - [TTL11_HUMAN]	1.00	1.00	1.05	3.86
	▲	O14561	Acyl carrier protein, mitochondrial OS=Homo sapiens GN=NDUFAB1 PE=1 SV=3 - [ACPM_HUMAN]	2.00	2.00	1.28	3.86
	▲	Q8NI22	Multiple coagulation factor deficiency protein 2 OS=Homo sapiens GN=MCFD2 PE=1 SV=1 - [MCFD2_HUM	1.00	1.00	1.67	3.84
	▲	Q96FJ2	Dynein light chain 2, cytoplasmic OS=Homo sapiens GN=DYNLL2 PE=1 SV=1 - [DYL2_HUMAN]	2.00	3.00	0.95	3.80
	▲	P01857	Ig gamma-1 chain C region OS=Homo sapiens GN=IGHG1 PE=1 SV=1 - [IGHG1_HUMAN]	10.00	21.00	1.69	3.78
	▲	Q14061	Cytochrome c oxidase copper chaperone OS=Homo sapiens GN=COX17 PE=1 SV=2 - [COX17_HUMAN]	2.00	2.00	1.74	3.77

	▲	Q86SX6	Glutaredoxin-related protein 5, mitochondrial OS=Homo sapiens GN=GLRX5 PE=1 SV=2 - [GLRX5_HUMAN	1.00	1.00	1.80	3.77
	▲	P84243	Histone H3.3 OS=Homo sapiens GN=H3F3A PE=1 SV=2 - [H33_HUMAN]	1.00	1.00	1.26	3.74
	▲	O00244	Copper transport protein ATOX1 OS=Homo sapiens GN=ATOX1 PE=1 SV=1 - [ATOX1_HUMAN]	3.00	3.00	1.24	3.74
	▲	P01861	Ig gamma-4 chain C region OS=Homo sapiens GN=IGHG4 PE=1 SV=1 - [IGHG4_HUMAN]	7.00	14.00	1.16	3.71
	▲	P02671	Fibrinogen alpha chain OS=Homo sapiens GN=FGA PE=1 SV=2 - [FIBA_HUMAN]	33.00	33.00	1.79	3.66
	▲	Q13451	Peptidyl-prolyl cis-trans isomerase FKBP5 OS=Homo sapiens GN=FKBP5 PE=1 SV=2 - [FKBP5_HUMAN]	3.00	3.00	1.69	3.63
	▲	P07451	Carbonic anhydrase 3 OS=Homo sapiens GN=CA3 PE=1 SV=3 - [CAH3_HUMAN]	6.00	6.00	1.98	3.63
	▲	Q969T9	WW domain-binding protein 2 OS=Homo sapiens GN=WBP2 PE=1 SV=1 - [WBP2_HUMAN]	2.00	2.00	1.68	3.63
	▲	P00742	Coagulation factor X OS=Homo sapiens GN=F10 PE=1 SV=2 - [FA10_HUMAN]	3.00	3.00	1.31	3.62
	▲	P08590	Myosin light chain 3 OS=Homo sapiens GN=MYL3 PE=1 SV=3 - [MYL3_HUMAN]	4.00	4.00	1.70	3.61
	▲	P52758	Ribonuclease UK114 OS=Homo sapiens GN=HRSP12 PE=1 SV=1 - [UK114_HUMAN]	6.00	6.00	1.14	3.58
	▲	P30048	Thioredoxin-dependent peroxide reductase, mitochondrial OS=Homo sapiens GN=PRDX3 PE=1 SV=3 - [PRDX	3.00	3.00	1.36	3.55
	▲	P09012	U1 small nuclear ribonucleoprotein A OS=Homo sapiens GN=SNRPA PE=1 SV=3 - [SNRPA_HUMAN]	1.00	1.00	1.43	3.54
	▲	A6NKN8	Purkinje cell protein 4-like protein 1 OS=Homo sapiens GN=PCP4L1 PE=2 SV=3 - [PC4L1_HUMAN]	1.00	1.00	1.74	3.54
	▲	P01763	Ig heavy chain V-III region WEA OS=Homo sapiens PE=1 SV=1 - [HV302_HUMAN]	1.00	2.00	1.52	3.53
	▲	Q6UWP8	Suprabasin OS=Homo sapiens GN=SBSN PE=2 SV=1 - [SBSN_HUMAN]	4.00	4.00	1.34	3.46
	▲	P02766	Transthyretin OS=Homo sapiens GN=TTR PE=1 SV=1 - [TTHY_HUMAN]	10.00	10.00	1.49	3.46
	▲	P01037	Cystatin-SN OS=Homo sapiens GN=CST1 PE=1 SV=2 - [CYTN_HUMAN]	3.00	5.00	1.29	3.46
	▲	P13671	Complement component C6 OS=Homo sapiens GN=C6 PE=1 SV=3 - [CO6_HUMAN]	16.00	16.00	1.97	3.45
	▲	Q99969	Retinoic acid receptor responder protein 2 OS=Homo sapiens GN=RARRES2 PE=1 SV=1 - [RARR2_HUMAN]	4.00	4.00	0.76	3.45
	▲	Q8WW12	PEST proteolytic signal-containing nuclear protein OS=Homo sapiens GN=PCNP PE=1 SV=2 - [PCNP_HUMA	1.00	1.00	1.49	3.43
	▲	P01019	Angiotensinogen OS=Homo sapiens GN=AGT PE=1 SV=1 - [ANGT_HUMAN]	14.00	14.00	1.92	3.40
	▲	P63208	S-phase kinase-associated protein 1 OS=Homo sapiens GN=SKP1 PE=1 SV=2 - [SKP1_HUMAN]	11.00	11.00	1.38	3.39
	▲	Q9UKK9	ADP-sugar pyrophosphatase OS=Homo sapiens GN=NUDT5 PE=1 SV=1 - [NUDT5_HUMAN]	4.00	4.00	1.61	3.37
	▲	Q15149	Plectin-1 OS=Homo sapiens GN=PLEC1 PE=1 SV=3 - [PLEC1_HUMAN]	3.00	3.00	1.93	3.33
	▲	P09493	Tropomyosin alpha-1 chain OS=Homo sapiens GN=TPM1 PE=1 SV=2 - [TPM1_HUMAN]	1.00	4.00	1.53	3.31
	▲	P02747	Complement C1q subcomponent subunit C OS=Homo sapiens GN=C1QC PE=1 SV=3 - [C1QC_HUMAN]	1.00	1.00	1.74	3.30
	▲	P01031	Complement C5 OS=Homo sapiens GN=C5 PE=1 SV=4 - [CO5_HUMAN]	21.00	21.00	1.74	3.30
	▲	P50552	Vasodilator-stimulated phosphoprotein OS=Homo sapiens GN=VASP PE=1 SV=3 - [VASP_HUMAN]	3.00	3.00	1.39	3.29
	▲	P22061	Protein-L-isoaspartate(D-aspartate) O-methyltransferase OS=Homo sapiens GN=PCMT1 PE=1 SV=3 - [PIMT_]	4.00	4.00	1.29	3.27
	▲	Q9NS69	Mitochondrial import receptor subunit TOM22 homolog OS=Homo sapiens GN=TOMM22 PE=1 SV=3 - [TOM	1.00	1.00	1.46	3.25
	▲	P01771	Ig heavy chain V-III region HIL OS=Homo sapiens PE=1 SV=1 - [HV310_HUMAN]	2.00	2.00	1.72	3.25
	▲	P35749	Myosin-11 OS=Homo sapiens GN=MYH11 PE=1 SV=3 - [MYH11_HUMAN]	5.00	6.00	1.59	3.23
	▲	Q92496	Complement factor H-related protein 4 OS=Homo sapiens GN=CFHR4 PE=1 SV=2 - [FHR4_HUMAN]	4.00	4.00	1.84	3.23
	▲	Q02223	Tumor necrosis factor receptor superfamily member 17 OS=Homo sapiens GN=TNFRSF17 PE=1 SV=1 - [TNR	1.00	1.00	0.89	3.23
	▲	P08709	Coagulation factor VII OS=Homo sapiens GN=F7 PE=1 SV=1 - [FA7_HUMAN]	5.00	5.00	1.74	3.22

	▲	Q96S96	Phosphatidylethanolamine-binding protein 4 OS=Homo sapiens GN=PEBP4 PE=1 SV=3 - [PEBP4_HUMAN]	7.00	7.00	1.18	3.21
	▲	Q86X29	Lipolysis-stimulated lipoprotein receptor OS=Homo sapiens GN=LSR PE=1 SV=4 - [LSR_HUMAN]	6.00	6.00	1.33	3.20
	▲	P82930	28S ribosomal protein S34, mitochondrial OS=Homo sapiens GN=MRPS34 PE=1 SV=2 - [RT34_HUMAN]	1.00	1.00	1.39	3.20
	▲	P23396	40S ribosomal protein S3 OS=Homo sapiens GN=RPS3 PE=1 SV=2 - [RS3_HUMAN]	1.00	1.00	0.89	3.19
	▲	Q9UN36	Protein NDRG2 OS=Homo sapiens GN=NDRG2 PE=1 SV=2 - [NDRG2_HUMAN]	2.00	2.00	1.17	3.18
	▲	P11171	Protein 4.1 OS=Homo sapiens GN=EPB41 PE=1 SV=4 - [41_HUMAN]	1.00	1.00	0.85	3.18
	▲	Q13508	Ecto-ADP-ribosyltransferase 3 OS=Homo sapiens GN=ART3 PE=1 SV=2 - [NAR3_HUMAN]	11.00	11.00	1.85	3.18
	▲	P01815	Ig heavy chain V-II region COR OS=Homo sapiens PE=1 SV=1 - [HV202_HUMAN]	1.00	1.00	1.84	3.17
	▲	O95084	Serine protease 23 OS=Homo sapiens GN=PRSS23 PE=1 SV=1 - [PRS23_HUMAN]	2.00	2.00	1.19	3.15
	▲	Q92688	Acidic leucine-rich nuclear phosphoprotein 32 family member B OS=Homo sapiens GN=ANP32B PE=1 SV=1 -	1.00	1.00	1.16	3.13
	▲	Q641Q3	Meteorin-like protein OS=Homo sapiens GN=METRNL PE=2 SV=1 - [METRL_HUMAN]	1.00	1.00	1.09	3.10
	▲	Q86UE4	Protein LYRIC OS=Homo sapiens GN=MTDH PE=1 SV=2 - [LYRIC_HUMAN]	2.00	2.00	0.85	3.09
	▲	Q96HC4	PDZ and LIM domain protein 5 OS=Homo sapiens GN=PDLIM5 PE=1 SV=4 - [PDLI5_HUMAN]	2.00	2.00	1.24	3.08
	▲	P37802	Transgelin-2 OS=Homo sapiens GN=TAGLN2 PE=1 SV=3 - [TAGL2_HUMAN]	8.00	8.00	0.73	3.08
	▲	P81605	Dermcidin OS=Homo sapiens GN=DCD PE=1 SV=2 - [DCD_HUMAN]	7.00	7.00	1.32	3.07
	▲	Q96L50	Peptidylprolyl isomerase-like 5 OS=Homo sapiens GN=PPIL5 PE=1 SV=2 - [LLR1_HUMAN]	1.00	1.00	1.42	3.07
	▲	P49908	Selenoprotein P OS=Homo sapiens GN=SEPP1 PE=1 SV=3 - [SEPP1_HUMAN]	5.00	5.00	1.68	3.05
	▲	Q01524	Defensin-6 OS=Homo sapiens GN=DEFA6 PE=1 SV=1 - [DEF6_HUMAN]	2.00	2.00	1.88	3.04
	▲	Q9P2E9	Ribosome-binding protein 1 OS=Homo sapiens GN=RRBP1 PE=1 SV=4 - [RRBP1_HUMAN]	6.00	6.00	1.43	3.04
	▲	P42167	Lamina-associated polypeptide 2, isoforms beta/gamma OS=Homo sapiens GN=TMPO PE=1 SV=2 - [LAP2B_	1.00	1.00	1.11	3.04
	▲	Q86TD4	Sarcalumenin OS=Homo sapiens GN=SRL PE=2 SV=2 - [SRCA_HUMAN]	2.00	2.00	1.48	3.04
	▲	P78539	Sushi repeat-containing protein SRPX OS=Homo sapiens GN=SRPX PE=2 SV=1 - [SRPX_HUMAN]	1.00	1.00	1.58	3.02
	▲	Q92932	Receptor-type tyrosine-protein phosphatase N2 OS=Homo sapiens GN=PTPRN2 PE=1 SV=2 - [PTPR2_HUMA	3.00	3.00	1.67	3.01
	▲	Q99972	Myocilin OS=Homo sapiens GN=MYOC PE=1 SV=2 - [MYOC_HUMAN]	11.00	11.00	1.50	3.00
	▲	Q8NHM4	Putative trypsin-6 OS=Homo sapiens GN=TRY6 PE=5 SV=1 - [TRY6_HUMAN]	1.00	3.00	1.40	3.00
	▲	P07237	Protein disulfide-isomerase OS=Homo sapiens GN=P4HB PE=1 SV=3 - [PDIA1_HUMAN]	11.00	11.00	1.52	2.98
	▲	Q9H8Y8	Golgi reassembly-stacking protein 2 OS=Homo sapiens GN=GORASP2 PE=1 SV=3 - [GORS2_HUMAN]	1.00	1.00	1.71	2.98
	▲	Q8N8N7	Prostaglandin reductase 2 OS=Homo sapiens GN=PTGR2 PE=1 SV=1 - [PTGR2_HUMAN]	7.00	7.00	1.27	2.96
	▲	P56159	GDNF family receptor alpha-1 OS=Homo sapiens GN=GFRA1 PE=2 SV=2 - [GFRA1_HUMAN]	1.00	1.00	1.45	2.96
	▲	P00451	Coagulation factor VIII OS=Homo sapiens GN=F8 PE=1 SV=1 - [FA8_HUMAN]	1.00	1.00	1.59	2.96
	▲	Q13361	Microfibrillar-associated protein 5 OS=Homo sapiens GN=MFAP5 PE=1 SV=1 - [MFAP5_HUMAN]	3.00	3.00	1.89	2.94
	▲	P30508	HLA class I histocompatibility antigen, Cw-12 alpha chain OS=Homo sapiens GN=HLA-C PE=1 SV=2 - [1C12	3.00	5.00	1.31	2.94
	▲	P24666	Low molecular weight phosphotyrosine protein phosphatase OS=Homo sapiens GN=ACP1 PE=1 SV=3 - [PPAC	2.00	2.00	0.80	2.93
	▲	Q9UBY9	Heat shock protein beta-7 OS=Homo sapiens GN=HSPB7 PE=1 SV=1 - [HSPB7_HUMAN]	1.00	1.00	1.75	2.92
	▲	P01343	Insulin-like growth factor IA OS=Homo sapiens GN=IGF1 PE=1 SV=1 - [IGF1A_HUMAN]	2.00	2.00	1.46	2.91
	▲	P09486	SPARC OS=Homo sapiens GN=SPARC PE=1 SV=1 - [SPRC_HUMAN]	1.00	1.00	1.82	2.91

	▲	Q71DI3	Histone H3.2 OS=Homo sapiens GN=HIST2H3A PE=1 SV=3 - [H32_HUMAN]	1.00	1.00	1.37	2.91
	▲	Q09666	Neuroblast differentiation-associated protein AHNAK OS=Homo sapiens GN=AHNAK PE=1 SV=2 - [AHNK_	61.00	61.00	1.45	2.90
	▲	O60506	Heterogeneous nuclear ribonucleoprotein Q OS=Homo sapiens GN=SYNCRIP PE=1 SV=2 - [HNRPQ_HUMA	1.00	1.00	1.08	2.90
	▲	P61978	Heterogeneous nuclear ribonucleoprotein K OS=Homo sapiens GN=HNRNPK PE=1 SV=1 - [HNRPK_HUMA	1.00	1.00	0.90	2.90
	▲	Q96C90	Protein phosphatase 1 regulatory subunit 14B OS=Homo sapiens GN=PPP1R14B PE=1 SV=3 - [PP14B_HUMA	1.00	1.00	1.50	2.89
	▲	P00167	Cytochrome b5 OS=Homo sapiens GN=CYB5A PE=1 SV=2 - [CYB5_HUMAN]	2.00	2.00	1.76	2.89
	▲	P20908	Collagen alpha-1(V) chain OS=Homo sapiens GN=COL5A1 PE=1 SV=3 - [CO5A1_HUMAN]	2.00	2.00	1.55	2.87
	▲	Q9UKY7	Protein CDV3 homolog OS=Homo sapiens GN=CDV3 PE=1 SV=1 - [CDV3_HUMAN]	3.00	3.00	1.26	2.85
	▲	P50281	Matrix metalloproteinase-14 OS=Homo sapiens GN=MMP14 PE=1 SV=2 - [MMP14_HUMAN]	1.00	1.00	1.23	2.84
	▲	P37840	Alpha-synuclein OS=Homo sapiens GN=SNCA PE=1 SV=1 - [SYUA_HUMAN]	4.00	4.00	1.93	2.83
	▲	O75781	Paralemmin OS=Homo sapiens GN=PALM PE=1 SV=2 - [PALM_HUMAN]	3.00	3.00	1.23	2.83
	▲	Q07812	Apoptosis regulator BAX OS=Homo sapiens GN=BAX PE=1 SV=1 - [BAX_HUMAN]	4.00	4.00	1.74	2.83
	▲	Q92575	UBX domain-containing protein 4 OS=Homo sapiens GN=UBXN4 PE=1 SV=2 - [UBXN4_HUMAN]	1.00	1.00	1.58	2.82
	▲	Q9NRR1	Cytokine-like protein 1 OS=Homo sapiens GN=CYTL1 PE=1 SV=1 - [CYTL1_HUMAN]	2.00	2.00	1.29	2.81
	▲	P35754	Glutaredoxin-1 OS=Homo sapiens GN=GLRX PE=1 SV=2 - [GLRX1_HUMAN]	5.00	5.00	1.43	2.81
	▲	P35579	Myosin-9 OS=Homo sapiens GN=MYH9 PE=1 SV=4 - [MYH9_HUMAN]	13.00	14.00	1.46	2.81
	▲	P01008	Antithrombin-III OS=Homo sapiens GN=SERPINC1 PE=1 SV=1 - [ANT3_HUMAN]	27.00	27.00	1.86	2.80
	▲	Q99497	Protein DJ-1 OS=Homo sapiens GN=PARK7 PE=1 SV=2 - [PARK7_HUMAN]	11.00	11.00	0.89	2.79
	▲	P02749	Beta-2-glycoprotein 1 OS=Homo sapiens GN=APOH PE=1 SV=3 - [APOH_HUMAN]	17.00	17.00	1.39	2.78
	▲	P33241	Lymphocyte-specific protein 1 OS=Homo sapiens GN=LSP1 PE=1 SV=1 - [LSP1_HUMAN]	3.00	3.00	1.29	2.78
	▲	O00763	Acetyl-CoA carboxylase 2 OS=Homo sapiens GN=ACACB PE=1 SV=2 - [ACACB_HUMAN]	1.00	1.00	0.80	2.76
	▲	Q9HD89	Resistin OS=Homo sapiens GN=RETN PE=2 SV=1 - [RETN_HUMAN]	5.00	5.00	1.26	2.75
	▲	P55058	Phospholipid transfer protein OS=Homo sapiens GN=PLTP PE=1 SV=1 - [PLTP_HUMAN]	4.00	4.00	1.45	2.74
	▲	O14817	Tetraspanin-4 OS=Homo sapiens GN=TSPAN4 PE=1 SV=1 - [TSN4_HUMAN]	1.00	1.00	1.44	2.73
	▲	O00479	High mobility group nucleosome-binding domain-containing protein 4 OS=Homo sapiens GN=HMGN4 PE=1 S	1.00	1.00	1.31	2.72
	▲	P01743	Ig heavy chain V-I region HG3 OS=Homo sapiens PE=4 SV=1 - [HV102_HUMAN]	2.00	3.00	1.76	2.72
	▲	O60664	Perilipin-3 OS=Homo sapiens GN=PLIN3 PE=1 SV=2 - [PLIN3_HUMAN]	7.00	7.00	1.21	2.70
	▲	P47897	Glutaminyl-tRNA synthetase OS=Homo sapiens GN=QARS PE=1 SV=1 - [SYQ_HUMAN]	1.00	1.00	1.35	2.70
	▲	O60814	Histone H2B type 1-K OS=Homo sapiens GN=HIST1H2BK PE=1 SV=3 - [H2B1K_HUMAN]	2.00	2.00	1.69	2.69
	▲	P01859	Ig gamma-2 chain C region OS=Homo sapiens GN=IGHG2 PE=1 SV=2 - [IGHG2_HUMAN]	8.00	16.00	2.00	2.66
	▲	O43237	Cytoplasmic dynein 1 light intermediate chain 2 OS=Homo sapiens GN=DYNC1LI2 PE=1 SV=1 - [DC1L2_HU	1.00	1.00	1.90	2.66
	▲	P78563	Double-stranded RNA-specific editase 1 OS=Homo sapiens GN=ADARB1 PE=1 SV=1 - [RED1_HUMAN]	1.00	1.00	1.18	2.65
	▲	Q9UDY2	Tight junction protein ZO-2 OS=Homo sapiens GN=TJP2 PE=1 SV=1 - [ZO2_HUMAN]	1.00	1.00	1.02	2.63
	▲	Q9Y4L1	Hypoxia up-regulated protein 1 OS=Homo sapiens GN=HYOU1 PE=1 SV=1 - [HYOU1_HUMAN]	9.00	9.00	1.51	2.63
	▲	Q9Y5C1	Angiopoietin-related protein 3 OS=Homo sapiens GN=ANGPTL3 PE=1 SV=1 - [ANGL3_HUMAN]	4.00	4.00	1.65	2.63
	▲	P04070	Vitamin K-dependent protein C OS=Homo sapiens GN=PROC PE=1 SV=1 - [PROC_HUMAN]	2.00	2.00	0.93	2.61

	▲	Q15738	Sterol-4-alpha-carboxylate 3-dehydrogenase, decarboxylating OS=Homo sapiens GN=NSDHL PE=1 SV=2 - [N	1.00	1.00	1.58	2.60
	▲	P49257	Protein ERGIC-53 OS=Homo sapiens GN=LMAN1 PE=1 SV=2 - [LMAN1_HUMAN]	2.00	2.00	1.38	2.59
	▲	Q9BSH5	Haloacid dehalogenase-like hydrolase domain-containing protein 3 OS=Homo sapiens GN=HDHD3 PE=1 SV=1	1.00	1.00	1.41	2.59
	▲	Q16891	Mitochondrial inner membrane protein OS=Homo sapiens GN=IMMT PE=1 SV=1 - [IMMT_HUMAN]	2.00	2.00	0.95	2.55
	▲	Q86YT9	Junctional adhesion molecule-like OS=Homo sapiens GN=AMICA1 PE=1 SV=1 - [JAML1_HUMAN]	1.00	1.00	1.59	2.55
	▲	P18065	Insulin-like growth factor-binding protein 2 OS=Homo sapiens GN=IGFBP2 PE=1 SV=2 - [IBP2_HUMAN]	10.00	10.00	1.21	2.55
	▲	P00748	Coagulation factor XII OS=Homo sapiens GN=F12 PE=1 SV=2 - [FA12_HUMAN]	6.00	6.00	1.18	2.55
	▲	P25325	3-mercaptopyruvate sulfurtransferase OS=Homo sapiens GN=MPST PE=1 SV=3 - [THTM_HUMAN]	7.00	7.00	1.01	2.54
	▲	Q14574	Desmocollin-3 OS=Homo sapiens GN=DSC3 PE=1 SV=3 - [DSC3_HUMAN]	3.00	3.00	0.98	2.53
	▲	Q9UHD8	Septin-9 OS=Homo sapiens GN=SEPT9 PE=1 SV=2 - [SEPT9_HUMAN]	1.00	1.00	1.97	2.53
	▲	P41439	Folate receptor gamma OS=Homo sapiens GN=FOLR3 PE=1 SV=1 - [FOLR3_HUMAN]	3.00	5.00	0.78	2.53
	▲	O15372	Eukaryotic translation initiation factor 3 subunit H OS=Homo sapiens GN=EIF3H PE=1 SV=1 - [EIF3H_HUMA	1.00	1.00	0.67	2.52
	▲	P29279	Connective tissue growth factor OS=Homo sapiens GN=CTGF PE=1 SV=2 - [CTGF_HUMAN]	8.00	8.00	1.70	2.52
	▲	P52597	Heterogeneous nuclear ribonucleoprotein F OS=Homo sapiens GN=HNRNPF PE=1 SV=3 - [HNRPF_HUMAN	1.00	1.00	1.30	2.52
	▲	O43390	Heterogeneous nuclear ribonucleoprotein R OS=Homo sapiens GN=HNRNPR PE=1 SV=1 - [HNRPR_HUMAN	2.00	2.00	1.22	2.51
	▲	P38117	Electron transfer flavoprotein subunit beta OS=Homo sapiens GN=ETFB PE=1 SV=3 - [ETFB_HUMAN]	1.00	1.00	1.18	2.51
	▲	Q99439	Calponin-2 OS=Homo sapiens GN=CNN2 PE=1 SV=4 - [CNN2_HUMAN]	1.00	1.00	1.31	2.51
	▲	P31146	Coronin-1A OS=Homo sapiens GN=CORO1A PE=1 SV=4 - [COR1A_HUMAN]	2.00	2.00	1.91	2.51
	▲	Q15847	Adipose most abundant gene transcript 2 protein OS=Homo sapiens GN=APM2 PE=1 SV=1 - [APM2_HUMAN	5.00	5.00	1.85	2.50
	▲	Q15942	Zyxin OS=Homo sapiens GN=ZYX PE=1 SV=1 - [ZYX_HUMAN]	3.00	3.00	1.75	2.50
	▲	P60985	Keratinocyte differentiation-associated protein OS=Homo sapiens GN=KRTDAP PE=1 SV=1 - [KTDAP_HUM	1.00	1.00	0.98	2.50
	▲	Q9NP55	Protein Plunc OS=Homo sapiens GN=PLUNC PE=1 SV=1 - [PLUNC_HUMAN]	4.00	4.00	1.44	2.49
	▲	Q687X5	Metalloreductase STEAP4 OS=Homo sapiens GN=STEAP4 PE=1 SV=1 - [STEA4_HUMAN]	1.00	1.00	1.29	2.49
	▲	Q15517	Corneodesmosin OS=Homo sapiens GN=CDSN PE=1 SV=3 - [CDSN_HUMAN]	4.00	4.00	1.73	2.49
	▲	O14983	Sarcoplasmic/endoplasmic reticulum calcium ATPase 1 OS=Homo sapiens GN=ATP2A1 PE=1 SV=1 - [AT2A1	1.00	1.00	1.42	2.48
	▲	Q9H361	Polyadenylate-binding protein 3 OS=Homo sapiens GN=PABPC3 PE=1 SV=2 - [PABP3_HUMAN]	2.00	2.00	1.32	2.47
	▲	Q9BVG4	UPF0368 protein Cxorf26 OS=Homo sapiens GN=CXorf26 PE=1 SV=1 - [CX026_HUMAN]	1.00	1.00	1.13	2.46
	▲	P38646	Stress-70 protein, mitochondrial OS=Homo sapiens GN=HSPA9 PE=1 SV=2 - [GRP75_HUMAN]	2.00	2.00	1.57	2.45
	▲	Q9Y279	V-set and immunoglobulin domain-containing protein 4 OS=Homo sapiens GN=VSIG4 PE=1 SV=1 - [VSIG4_I	9.00	9.00	1.56	2.45
	▲	Q6E0U4	Dermokine OS=Homo sapiens GN=DMKN PE=1 SV=3 - [DMKN_HUMAN]	4.00	4.00	1.69	2.45
	▲	P62993	Growth factor receptor-bound protein 2 OS=Homo sapiens GN=GRB2 PE=1 SV=1 - [GRB2_HUMAN]	3.00	3.00	1.52	2.44
	▲	P02743	Serum amyloid P-component OS=Homo sapiens GN=APCS PE=1 SV=2 - [SAMP_HUMAN]	5.00	5.00	1.31	2.43
	▲	P19338	Nucleolin OS=Homo sapiens GN=NCL PE=1 SV=3 - [NUCL_HUMAN]	2.00	2.00	0.96	2.43
	▲	P18136	Ig kappa chain V-III region HIC OS=Homo sapiens PE=2 SV=1 - [KV313_HUMAN]	1.00	6.00	1.73	2.41
	▲	P37837	Transaldolase OS=Homo sapiens GN=TALDO1 PE=1 SV=2 - [TALDO_HUMAN]	5.00	5.00	1.51	2.40
	▲	Q5THK1	Uncharacterized protein C22orf30 OS=Homo sapiens GN=C22orf30 PE=1 SV=1 - [CV030_HUMAN]	1.00	1.00	1.48	2.40

	▲	P35527	Keratin, type I cytoskeletal 9 OS=Homo sapiens GN=KRT9 PE=1 SV=3 - [K1C9_HUMAN]	12.00	13.00	1.50	2.39
	▲	Q9Y3C8	Ubiquitin-fold modifier-conjugating enzyme 1 OS=Homo sapiens GN=UFC1 PE=1 SV=3 - [UFC1_HUMAN]	2.00	2.00	1.33	2.39
	▲	P00738	Haptoglobin OS=Homo sapiens GN=HP PE=1 SV=1 - [HPT_HUMAN]	12.00	23.00	1.45	2.39
	▲	Q7Z434	Mitochondrial antiviral-signaling protein OS=Homo sapiens GN=MAVS PE=1 SV=2 - [MAVS_HUMAN]	1.00	1.00	1.39	2.38
	▲	O14791	Apolipoprotein L1 OS=Homo sapiens GN=APOL1 PE=1 SV=5 - [APOL1_HUMAN]	3.00	3.00	1.23	2.37
	▲	Q10567	AP-1 complex subunit beta-1 OS=Homo sapiens GN=AP1B1 PE=1 SV=2 - [AP1B1_HUMAN]	3.00	5.00	1.22	2.37
	▲	Q16643	Drebrin OS=Homo sapiens GN=DBN1 PE=1 SV=4 - [DREB_HUMAN]	2.00	2.00	1.63	2.36
	▲	P14866	Heterogeneous nuclear ribonucleoprotein L OS=Homo sapiens GN=HNRNPL PE=1 SV=2 - [HNRPL_HUMAN	1.00	1.00	1.11	2.35
	▲	Q86V88	Magnesium-dependent phosphatase 1 OS=Homo sapiens GN=MDP1 PE=1 SV=1 - [MGDP1_HUMAN]	2.00	2.00	1.08	2.35
	▲	Q14696	LDLR chaperone MESD OS=Homo sapiens GN=MESDC2 PE=1 SV=2 - [MESD_HUMAN]	1.00	1.00	1.28	2.35
	▲	P04003	C4b-binding protein alpha chain OS=Homo sapiens GN=C4BPA PE=1 SV=2 - [C4BPA_HUMAN]	4.00	4.00	1.39	2.34
	▲	O75223	Gamma-glutamylcyclotransferase OS=Homo sapiens GN=GGCT PE=1 SV=1 - [GGCT_HUMAN]	7.00	7.00	1.12	2.34
	▲	O95477	ATP-binding cassette sub-family A member 1 OS=Homo sapiens GN=ABCA1 PE=1 SV=2 - [ABCA1_HUMAN	1.00	1.00	1.37	2.34
	▲	Q9NR12	PDZ and LIM domain protein 7 OS=Homo sapiens GN=PDLIM7 PE=1 SV=1 - [PDLI7_HUMAN]	2.00	2.00	1.57	2.32
	▲	Q6IBS0	Twinfilin-2 OS=Homo sapiens GN=TWF2 PE=1 SV=2 - [TWF2_HUMAN]	2.00	2.00	1.36	2.32
	▲	P01708	Ig lambda chain V-II region BUR OS=Homo sapiens PE=1 SV=1 - [LV205_HUMAN]	1.00	2.00	1.97	2.31
	▲	O75367	Core histone macro-H2A.1 OS=Homo sapiens GN=H2AFY PE=1 SV=4 - [H2AY_HUMAN]	1.00	1.00	1.69	2.29
	▲	P30101	Protein disulfide-isomerase A3 OS=Homo sapiens GN=PDIA3 PE=1 SV=4 - [PDIA3_HUMAN]	6.00	6.00	1.37	2.29
	▲	O15467	C-C motif chemokine 16 OS=Homo sapiens GN=CCL16 PE=1 SV=1 - [CCL16_HUMAN]	1.00	1.00	1.08	2.28
	▲	P30740	Leukocyte elastase inhibitor OS=Homo sapiens GN=SERPINB1 PE=1 SV=1 - [ILEU_HUMAN]	7.00	7.00	1.31	2.28
	▲	Q8NBK3	Sulfatase-modifying factor 1 OS=Homo sapiens GN=SUMF1 PE=1 SV=3 - [SUMF1_HUMAN]	3.00	3.00	1.71	2.28
	▲	Q08830	Fibrinogen-like protein 1 OS=Homo sapiens GN=FGL1 PE=1 SV=3 - [FGL1_HUMAN]	2.00	2.00	1.04	2.27
	▲	P46976	Glycogenin-1 OS=Homo sapiens GN=GYG1 PE=1 SV=4 - [GLYG_HUMAN]	2.00	2.00	1.22	2.26
	▲	O43504	Hepatitis B virus X-interacting protein OS=Homo sapiens GN=HBXIP PE=1 SV=1 - [HBXIP_HUMAN]	2.00	2.00	1.16	2.26
	▲	O15145	Actin-related protein 2/3 complex subunit 3 OS=Homo sapiens GN=ARPC3 PE=1 SV=3 - [ARPC3_HUMAN]	2.00	2.00	0.94	2.24
	▲	P01596	Ig kappa chain V-I region CAR OS=Homo sapiens PE=1 SV=1 - [KV104_HUMAN]	2.00	3.00	1.43	2.24
	▲	Q9BV57	1,2-dihydroxy-3-keto-5-methylthiopentene dioxygenase OS=Homo sapiens GN=ADI1 PE=1 SV=1 - [MTND_H	1.00	1.00	1.10	2.24
	▲	Q9UKJ1	Paired immunoglobulin-like type 2 receptor alpha OS=Homo sapiens GN=PILRA PE=1 SV=3 - [PILRA_HUMA	2.00	2.00	1.84	2.24
	▲	Q06323	Proteasome activator complex subunit 1 OS=Homo sapiens GN=PSME1 PE=1 SV=1 - [PSME1_HUMAN]	2.00	2.00	1.48	2.23
	▲	P10599	Thioredoxin OS=Homo sapiens GN=TXN PE=1 SV=3 - [THIO_HUMAN]	7.00	7.00	1.16	2.22
	▲	P08727	Keratin, type I cytoskeletal 19 OS=Homo sapiens GN=KRT19 PE=1 SV=3 - [K1C19_HUMAN]	4.00	9.00	1.07	2.22
	▲	P26641	Elongation factor 1-gamma OS=Homo sapiens GN=EEF1G PE=1 SV=3 - [EF1G_HUMAN]	5.00	5.00	1.69	2.21
	▲	P80419	Ig heavy chain V-III region GAR OS=Homo sapiens PE=1 SV=1 - [HV322_HUMAN]	2.00	2.00	1.00	2.21
	▲	P61604	10 kDa heat shock protein, mitochondrial OS=Homo sapiens GN=HSPE1 PE=1 SV=2 - [CH10_HUMAN]	4.00	4.00	1.32	2.20
	▲	P02545	Lamin-A/C OS=Homo sapiens GN=LMNA PE=1 SV=1 - [LMNA_HUMAN]	4.00	4.00	1.63	2.20
	▲	P51884	Lumican OS=Homo sapiens GN=LUM PE=1 SV=2 - [LUM_HUMAN]	14.00	14.00	1.65	2.20

	▲	Q8IX05	CD302 antigen OS=Homo sapiens GN=CD302 PE=1 SV=1 - [CD302_HUMAN]	1.00	1.00	1.46	2.19
	▲	P61086	Ubiquitin-conjugating enzyme E2 K OS=Homo sapiens GN=UBE2K PE=1 SV=3 - [UBE2K_HUMAN]	4.00	4.00	0.99	2.19
	▲	O00339	Matrilin-2 OS=Homo sapiens GN=MATN2 PE=1 SV=3 - [MATN2_HUMAN]	1.00	1.00	1.78	2.19
	▲	O95633	Follistatin-related protein 3 OS=Homo sapiens GN=FSTL3 PE=1 SV=1 - [FSTL3_HUMAN]	4.00	4.00	1.55	2.18
	▲	Q15904	V-type proton ATPase subunit S1 OS=Homo sapiens GN=ATP6AP1 PE=1 SV=2 - [VAS1_HUMAN]	5.00	5.00	1.36	2.18
	▲	P07225	Vitamin K-dependent protein S OS=Homo sapiens GN=PROS1 PE=1 SV=1 - [PROS_HUMAN]	6.00	6.00	1.37	2.18
	▲	Q15843	NEDD8 OS=Homo sapiens GN=NEDD8 PE=1 SV=1 - [NEDD8_HUMAN]	1.00	1.00	0.82	2.17
	▲	P20039	HLA class II histocompatibility antigen, DRB1-11 beta chain OS=Homo sapiens GN=HLA-DRB1 PE=2 SV=1 -	2.00	2.00	0.53	2.17
	▲	Q9Y2V2	Calcium-regulated heat stable protein 1 OS=Homo sapiens GN=CARHSP1 PE=1 SV=2 - [CHSP1_HUMAN]	2.00	2.00	0.92	2.16
	▲	P29622	Kallistatin OS=Homo sapiens GN=SERPINA4 PE=1 SV=3 - [KAIN_HUMAN]	16.00	16.00	1.15	2.16
	▲	P54725	UV excision repair protein RAD23 homolog A OS=Homo sapiens GN=RAD23A PE=1 SV=1 - [RD23A_HUM	2.00	3.00	1.90	2.15
	▲	P52272	Heterogeneous nuclear ribonucleoprotein M OS=Homo sapiens GN=HNRNPM PE=1 SV=3 - [HNRPM_HUMA	2.00	2.00	1.04	2.15
	▲	Q5VY80	Retinoic acid early transcript 1L protein OS=Homo sapiens GN=RAET1L PE=2 SV=1 - [RET1L_HUMAN]	1.00	1.00	1.98	2.15
	▲	Q1KMD3	Heterogeneous nuclear ribonucleoprotein U-like protein 2 OS=Homo sapiens GN=HNRNPUL2 PE=1 SV=1 - [H	3.00	3.00	1.93	2.14
	▲	Q9P0L0	Vesicle-associated membrane protein-associated protein A OS=Homo sapiens GN=VAPA PE=1 SV=3 - [VAPA	1.00	1.00	1.31	2.14
	▲	Q08623	Haloacid dehalogenase-like hydrolase domain-containing protein 1A OS=Homo sapiens GN=HDHD1A PE=1 S	4.00	4.00	1.01	2.14
	▲	P01598	Ig kappa chain V-I region EU OS=Homo sapiens PE=1 SV=1 - [KV106_HUMAN]	2.00	3.00	1.68	2.14
	▲	P35858	Insulin-like growth factor-binding protein complex acid labile subunit OS=Homo sapiens GN=IGFALS PE=1 S	6.00	6.00	1.15	2.14
	▲	O76070	Gamma-synuclein OS=Homo sapiens GN=SNCG PE=1 SV=2 - [SYUG_HUMAN]	5.00	5.00	1.10	2.13
	▲	Q9UBP4	Dickkopf-related protein 3 OS=Homo sapiens GN=DKK3 PE=1 SV=1 - [DKK3_HUMAN]	5.00	5.00	1.59	2.13
	▲	P01600	Ig kappa chain V-I region Hau OS=Homo sapiens PE=1 SV=1 - [KV108_HUMAN]	1.00	2.00	1.58	2.13
	▲	P13796	Plastin-2 OS=Homo sapiens GN=LCP1 PE=1 SV=5 - [PLSL_HUMAN]	19.00	25.00	1.41	2.13
	▲	O43865	Putative adenosylhomocysteinase 2 OS=Homo sapiens GN=AHCYL1 PE=1 SV=2 - [SAHH2_HUMAN]	1.00	2.00	1.82	2.13
	▲	Q4KMP7	TBC1 domain family member 10B OS=Homo sapiens GN=TBC1D10B PE=1 SV=3 - [TB10B_HUMAN]	2.00	2.00	1.57	2.12
	▲	P06753	Tropomyosin alpha-3 chain OS=Homo sapiens GN=TPM3 PE=1 SV=1 - [TPM3_HUMAN]	1.00	4.00	1.69	2.12
	▲	Q86Y82	Syntaxin-12 OS=Homo sapiens GN=STX12 PE=1 SV=1 - [STX12_HUMAN]	1.00	1.00	0.93	2.12
	▲	P05156	Complement factor I OS=Homo sapiens GN=CFI PE=1 SV=1 - [CFAI_HUMAN]	19.00	19.00	1.81	2.12
	▲	P48052	Carboxypeptidase A2 OS=Homo sapiens GN=CPA2 PE=1 SV=3 - [CBPA2_HUMAN]	1.00	1.00	1.43	2.11
	▲	P02794	Ferritin heavy chain OS=Homo sapiens GN=FTH1 PE=1 SV=2 - [FRIH_HUMAN]	6.00	6.00	0.81	2.11
	▲	Q9NP84	Tumor necrosis factor receptor superfamily member 12A OS=Homo sapiens GN=TNFRSF12A PE=1 SV=1 - [T	1.00	1.00	1.86	2.10
	▲	O75347	Tubulin-specific chaperone A OS=Homo sapiens GN=TBCA PE=1 SV=3 - [TBCA_HUMAN]	5.00	5.00	0.55	2.10
	▲	P82980	Retinol-binding protein 5 OS=Homo sapiens GN=RBP5 PE=1 SV=3 - [RET5_HUMAN]	4.00	4.00	1.06	2.09
	▲	P09958	Furin OS=Homo sapiens GN=FURIN PE=1 SV=2 - [FURIN_HUMAN]	2.00	2.00	1.42	2.08
	▲	P58335	Anthrax toxin receptor 2 OS=Homo sapiens GN=ANTXR2 PE=1 SV=4 - [ANTR2_HUMAN]	3.00	3.00	1.87	2.08
	▲	P49773	Histidine triad nucleotide-binding protein 1 OS=Homo sapiens GN=HINT1 PE=1 SV=2 - [HINT1_HUMAN]	1.00	1.00	1.23	2.08
	▲	P51888	Prolargin OS=Homo sapiens GN=PRELP PE=1 SV=1 - [PRELP_HUMAN]	1.00	1.00	1.16	2.08

	▲	Q99952	Tyrosine-protein phosphatase non-receptor type 18 OS=Homo sapiens GN=PTPN18 PE=1 SV=2 - [PTN18_HU	1.00	1.00	1.03	2.08
	▲	Q9NUQ9	Protein FAM49B OS=Homo sapiens GN=FAM49B PE=1 SV=1 - [FA49B_HUMAN]	6.00	6.00	1.20	2.06
	▲	P12724	Eosinophil cationic protein OS=Homo sapiens GN=RNASE3 PE=1 SV=2 - [ECP_HUMAN]	3.00	3.00	1.38	2.05
	▲	Q9Y6W5	Wiskott-Aldrich syndrome protein family member 2 OS=Homo sapiens GN=WASF2 PE=1 SV=3 - [WASF2_H	1.00	1.00	1.19	2.05
	▲	O95861	3'(2'),5'-bisphosphate nucleotidase 1 OS=Homo sapiens GN=BPNT1 PE=1 SV=1 - [BPNT1_HUMAN]	7.00	7.00	1.57	2.05
	▲	P26368	Splicing factor U2AF 65 kDa subunit OS=Homo sapiens GN=U2AF2 PE=1 SV=4 - [U2AF2_HUMAN]	1.00	1.00	1.35	2.04
	▲	O95747	Serine/threonine-protein kinase OSR1 OS=Homo sapiens GN=OXSR1 PE=1 SV=1 - [OXSR1_HUMAN]	2.00	2.00	1.42	2.04
	▲	P21980	Protein-glutamine gamma-glutamyltransferase 2 OS=Homo sapiens GN=TGM2 PE=1 SV=2 - [TGM2_HUMAN	4.00	4.00	1.14	2.04
	▲	Q8TE58	A disintegrin and metalloproteinase with thrombospondin motifs 15 OS=Homo sapiens GN=ADAMTS15 PE=2	1.00	1.00	0.87	2.03
	▲	Q8WWI5	Choline transporter-like protein 1 OS=Homo sapiens GN=SLC44A1 PE=1 SV=1 - [CTL1_HUMAN]	1.00	1.00	1.82	2.03
	▲	Q96AB3	Isochorismatase domain-containing protein 2, mitochondrial OS=Homo sapiens GN=ISOC2 PE=1 SV=1 - [ISOC	1.00	1.00	1.51	2.02
	▲	P07307	Asialoglycoprotein receptor 2 OS=Homo sapiens GN=ASGR2 PE=1 SV=2 - [ASGR2_HUMAN]	5.00	5.00	1.49	2.02
	▲	Q9Y490	Talin-1 OS=Homo sapiens GN=TLN1 PE=1 SV=3 - [TLN1_HUMAN]	16.00	16.00	1.61	2.02
	▲	Q9C0C2	182 kDa tankyrase-1-binding protein OS=Homo sapiens GN=TNKS1BP1 PE=1 SV=3 - [TB182_HUMAN]	1.00	1.00	1.00	2.02
	▲	P01604	Ig kappa chain V-I region Kue OS=Homo sapiens PE=1 SV=1 - [KV112_HUMAN]	2.00	2.00	1.56	2.01
	▲	Q8N5J2	Protein FAM63A OS=Homo sapiens GN=FAM63A PE=1 SV=1 - [FA63A_HUMAN]	1.00	1.00	1.65	2.01
	▲	O60234	Glia maturation factor gamma OS=Homo sapiens GN=GMFG PE=1 SV=1 - [GMFG_HUMAN]	1.00	2.00	1.83	2.00
	▲	P04179	Superoxide dismutase [Mn], mitochondrial OS=Homo sapiens GN=SOD2 PE=1 SV=2 - [SODM_HUMAN]	5.00	5.00	1.14	2.00
		**Down-Regulated Proteins in two Subgroups (109)**					
▽	▽	P13647	Keratin, type II cytoskeletal 5 OS=Homo sapiens GN=KRT5 PE=1 SV=3 - [K2C5_HUMAN]	5.00	13.00	0.47	0.48
▽	▽	P54578	Ubiquitin carboxyl-terminal hydrolase 14 OS=Homo sapiens GN=USP14 PE=1 SV=3 - [UBP14_HUMAN]	1.00	1.00	0.36	0.48
▽	▽	P21281	V-type proton ATPase subunit B, brain isoform OS=Homo sapiens GN=ATP6V1B2 PE=1 SV=3 - [VATB2_HU	1.00	1.00	0.42	0.48
▽	▽	O43895	Xaa-Pro aminopeptidase 2 OS=Homo sapiens GN=XPNPEP2 PE=1 SV=3 - [XPP2_HUMAN]	3.00	3.00	0.47	0.48
▽	▽	Q9UGB7	Inositol oxygenase OS=Homo sapiens GN=MIOX PE=1 SV=1 - [MIOX_HUMAN]	2.00	2.00	0.34	0.47
▽	▽	P61457	Pterin-4-alpha-carbinolamine dehydratase OS=Homo sapiens GN=PCBD1 PE=1 SV=2 - [PHS_HUMAN]	1.00	1.00	0.29	0.47
▽	▽	P02538	Keratin, type II cytoskeletal 6A OS=Homo sapiens GN=KRT6A PE=1 SV=3 - [K2C6A_HUMAN]	5.00	14.00	0.40	0.47
▽	▽	Q66K79	Carboxypeptidase Z OS=Homo sapiens GN=CPZ PE=1 SV=2 - [CBPZ_HUMAN]	3.00	3.00	0.45	0.47
▽	▽	Q9Y6U3	Adseverin OS=Homo sapiens GN=SCIN PE=1 SV=4 - [ADSV_HUMAN]	2.00	2.00	0.47	0.46
▽	▽	P13797	Plastin-3 OS=Homo sapiens GN=PLS3 PE=1 SV=4 - [PLST_HUMAN]	4.00	10.00	0.48	0.44
▽	▽	P48061	Stromal cell-derived factor 1 OS=Homo sapiens GN=CXCL12 PE=1 SV=1 - [SDF1_HUMAN]	2.00	2.00	0.39	0.44
▽	▽	Q8N2U0	UPF0451 protein C17orf61 OS=Homo sapiens GN=C17orf61 PE=2 SV=1 - [CQ061_HUMAN]	2.00	2.00	0.33	0.44
▽	▽	Q9Y5K6	CD2-associated protein OS=Homo sapiens GN=CD2AP PE=1 SV=1 - [CD2AP_HUMAN]	2.00	2.00	0.49	0.44
▽	▽	Q68BL7	Olfactomedin-like protein 2A OS=Homo sapiens GN=OLFML2A PE=2 SV=1 - [OLM2A_HUMAN]	1.00	1.00	0.44	0.43
▽	▽	P15907	Beta-galactoside alpha-2,6-sialyltransferase 1 OS=Homo sapiens GN=ST6GAL1 PE=1 SV=1 - [SIAT1_HUMA	1.00	1.00	0.38	0.43
▽	▽	P37235	Hippocalcin-like protein 1 OS=Homo sapiens GN=HPCAL1 PE=1 SV=3 - [HPCL1_HUMAN]	2.00	2.00	0.43	0.43
▽	▽	P22732	Solute carrier family 2, facilitated glucose transporter member 5 OS=Homo sapiens GN=SLC2A5 PE=1 SV=1 -	4.00	4.00	0.47	0.42

▽	▽	Q9UL25	Ras-related protein Rab-21 OS=Homo sapiens GN=RAB21 PE=1 SV=3 - [RAB21_HUMAN]	1.00	1.00	0.50	0.42
▽	▽	P36543	V-type proton ATPase subunit E 1 OS=Homo sapiens GN=ATP6V1E1 PE=1 SV=1 - [VATE1_HUMAN]	4.00	4.00	0.49	0.41
▽	▽	Q86UN2	Reticulon-4 receptor-like 1 OS=Homo sapiens GN=RTN4RL1 PE=2 SV=1 - [R4RL1_HUMAN]	1.00	1.00	0.48	0.41
▽	▽	P55822	SH3 domain-binding glutamic acid-rich protein OS=Homo sapiens GN=SH3BGR PE=1 SV=3 - [SH3BG_HUM	1.00	1.00	0.39	0.39
▽	▽	Q9Y5H7	Protocadherin alpha-5 OS=Homo sapiens GN=PCDHA5 PE=1 SV=1 - [PCDA5_HUMAN]	2.00	2.00	0.49	0.39
▽	▽	O75695	Protein XRP2 OS=Homo sapiens GN=RP2 PE=1 SV=4 - [XRP2_HUMAN]	1.00	1.00	0.45	0.38
▽	▽	P01189	Pro-opiomelanocortin OS=Homo sapiens GN=POMC PE=1 SV=2 - [COLI_HUMAN]	2.00	2.00	0.38	0.38
▽	▽	P35321	Cornifin-A OS=Homo sapiens GN=SPRR1A PE=1 SV=2 - [SPR1A_HUMAN]	3.00	3.00	0.49	0.37
▽	▽	Q9NQS3	Poliovirus receptor-related protein 3 OS=Homo sapiens GN=PVRL3 PE=1 SV=1 - [PVRL3_HUMAN]	2.00	2.00	0.47	0.37
▽	▽	P08238	Heat shock protein HSP 90-beta OS=Homo sapiens GN=HSP90AB1 PE=1 SV=4 - [HS90B_HUMAN]	3.00	9.00	0.45	0.37
▽	▽	P08473	Neprilysin OS=Homo sapiens GN=MME PE=1 SV=2 - [NEP_HUMAN]	26.00	26.00	0.37	0.36
▽	▽	P56199	Integrin alpha-1 OS=Homo sapiens GN=ITGA1 PE=1 SV=2 - [ITA1_HUMAN]	1.00	1.00	0.50	0.36
▽	▽	A0AVF1	Tetratricopeptide repeat protein 26 OS=Homo sapiens GN=TTC26 PE=2 SV=1 - [TTC26_HUMAN]	1.00	1.00	0.48	0.36
▽	▽	Q8WWT9	Solute carrier family 13 member 3 OS=Homo sapiens GN=SLC13A3 PE=2 SV=1 - [S13A3_HUMAN]	1.00	1.00	0.49	0.35
▽	▽	P98161	Polycystin-1 OS=Homo sapiens GN=PKD1 PE=1 SV=3 - [PKD1_HUMAN]	1.00	1.00	0.48	0.35
▽	▽	P52434	DNA-directed RNA polymerases I, II, and III subunit RPABC3 OS=Homo sapiens GN=POLR2H PE=1 SV=4 -	1.00	1.00	0.46	0.35
▽	▽	P55017	Solute carrier family 12 member 3 OS=Homo sapiens GN=SLC12A3 PE=1 SV=2 - [S12A3_HUMAN]	6.00	6.00	0.42	0.35
▽	▽	O43511	Pendrin OS=Homo sapiens GN=SLC26A4 PE=1 SV=1 - [S26A4_HUMAN]	2.00	2.00	0.33	0.35
▽	▽	P14555	Phospholipase A2, membrane associated OS=Homo sapiens GN=PLA2G2A PE=1 SV=2 - [PA2GA_HUMAN]	1.00	1.00	0.30	0.34
▽	▽	P07602	Proactivator polypeptide OS=Homo sapiens GN=PSAP PE=1 SV=2 - [SAP_HUMAN]	18.00	18.00	0.45	0.33
▽	▽	Q495M3	Proton-coupled amino acid transporter 2 OS=Homo sapiens GN=SLC36A2 PE=2 SV=1 - [S36A2_HUMAN]	3.00	3.00	0.49	0.33
▽	▽	Q9H9H4	Vacuolar protein sorting-associated protein 37B OS=Homo sapiens GN=VPS37B PE=1 SV=1 - [VP37B_HUMA	2.00	2.00	0.46	0.33
▽	▽	P13646	Keratin, type I cytoskeletal 13 OS=Homo sapiens GN=KRT13 PE=1 SV=4 - [K1C13_HUMAN]	1.00	4.00	0.40	0.33
▽	▽	P29972	Aquaporin-1 OS=Homo sapiens GN=AQP1 PE=1 SV=3 - [AQP1_HUMAN]	2.00	2.00	0.47	0.33
▽	▽	P08758	Annexin A5 OS=Homo sapiens GN=ANXA5 PE=1 SV=2 - [ANXA5_HUMAN]	13.00	13.00	0.42	0.33
▽	▽	P55209	Nucleosome assembly protein 1-like 1 OS=Homo sapiens GN=NAP1L1 PE=1 SV=1 - [NP1L1_HUMAN]	1.00	1.00	0.43	0.32
▽	▽	Q99584	Protein S100-A13 OS=Homo sapiens GN=S100A13 PE=1 SV=1 - [S10AD_HUMAN]	4.00	4.00	0.44	0.31
▽	▽	P07288	Prostate-specific antigen OS=Homo sapiens GN=KLK3 PE=1 SV=2 - [KLK3_HUMAN]	11.00	12.00	0.45	0.31
▽	▽	P08637	Low affinity immunoglobulin gamma Fc region receptor III-A OS=Homo sapiens GN=FCGR3A PE=2 SV=2 - [	1.00	2.00	0.39	0.30
▽	▽	Q8N163	Protein KIAA1967 OS=Homo sapiens GN=KIAA1967 PE=1 SV=2 - [K1967_HUMAN]	1.00	1.00	0.46	0.30
▽	▽	Q9ULC0	Endomucin OS=Homo sapiens GN=EMCN PE=1 SV=2 - [MUCEN_HUMAN]	3.00	3.00	0.50	0.30
▽	▽	O00194	Ras-related protein Rab-27B OS=Homo sapiens GN=RAB27B PE=1 SV=4 - [RB27B_HUMAN]	3.00	4.00	0.43	0.30
▽	▽	P08133	Annexin A6 OS=Homo sapiens GN=ANXA6 PE=1 SV=3 - [ANXA6_HUMAN]	3.00	3.00	0.42	0.30
▽	▽	P20337	Ras-related protein Rab-3B OS=Homo sapiens GN=RAB3B PE=1 SV=2 - [RAB3B_HUMAN]	3.00	4.00	0.35	0.30
▽	▽	Q9UIK5	Tomoregulin-2 OS=Homo sapiens GN=TMEFF2 PE=1 SV=1 - [TEFF2_HUMAN]	1.00	1.00	0.47	0.29
▽	▽	P16066	Atrial natriuretic peptide receptor 1 OS=Homo sapiens GN=NPR1 PE=1 SV=1 - [ANPRA_HUMAN]	1.00	1.00	0.44	0.29

▽	▽	P51654	Glypican-3 OS=Homo sapiens GN=GPC3 PE=1 SV=1 - [GPC3_HUMAN]	4.00	4.00	0.45	0.29
▽	▽	P47972	Neuronal pentraxin-2 OS=Homo sapiens GN=NPTX2 PE=1 SV=2 - [NPTX2_HUMAN]	1.00	1.00	0.43	0.28
▽	▽	P31431	Syndecan-4 OS=Homo sapiens GN=SDC4 PE=1 SV=2 - [SDC4_HUMAN]	4.00	4.00	0.50	0.28
▽	▽	Q13867	Bleomycin hydrolase OS=Homo sapiens GN=BLMH PE=1 SV=1 - [BLMH_HUMAN]	4.00	4.00	0.29	0.28
▽	▽	Q14108	Lysosome membrane protein 2 OS=Homo sapiens GN=SCARB2 PE=1 SV=2 - [SCRB2_HUMAN]	2.00	2.00	0.30	0.28
▽	▽	P23458	Tyrosine-protein kinase JAK1 OS=Homo sapiens GN=JAK1 PE=1 SV=2 - [JAK1_HUMAN]	1.00	1.00	0.45	0.27
▽	▽	Q9Y376	Calcium-binding protein 39 OS=Homo sapiens GN=CAB39 PE=1 SV=1 - [CAB39_HUMAN]	1.00	1.00	0.34	0.27
▽	▽	P30626	Sorcin OS=Homo sapiens GN=SRI PE=1 SV=1 - [SORCN_HUMAN]	4.00	4.00	0.23	0.27
▽	▽	Q8N4F0	Bactericidal/permeability-increasing protein-like 1 OS=Homo sapiens GN=BPIL1 PE=1 SV=2 - [BPIL1_HUMA	1.00	1.00	0.42	0.27
▽	▽	P21926	CD9 antigen OS=Homo sapiens GN=CD9 PE=1 SV=4 - [CD9_HUMAN]	3.00	3.00	0.35	0.26
▽	▽	Q6UX73	Uncharacterized protein C16orf89 OS=Homo sapiens GN=C16orf89 PE=2 SV=2 - [CP089_HUMAN]	7.00	7.00	0.44	0.26
▽	▽	O43692	Peptidase inhibitor 15 OS=Homo sapiens GN=PI15 PE=1 SV=1 - [PI15_HUMAN]	2.00	3.00	0.46	0.26
▽	▽	Q08188	Protein-glutamine gamma-glutamyltransferase E OS=Homo sapiens GN=TGM3 PE=1 SV=4 - [TGM3_HUMAN	4.00	4.00	0.37	0.25
▽	▽	Q9UK41	Vacuolar protein sorting-associated protein 28 homolog OS=Homo sapiens GN=VPS28 PE=1 SV=1 - [VPS28_	8.00	8.00	0.49	0.25
▽	▽	Q6IPM2	IQ domain-containing protein E OS=Homo sapiens GN=IQCE PE=1 SV=2 - [IQCE_HUMAN]	1.00	1.00	0.39	0.24
▽	▽	Q9BV36	Melanophilin OS=Homo sapiens GN=MLPH PE=1 SV=1 - [MELPH_HUMAN]	1.00	1.00	0.35	0.24
▽	▽	P55259	Pancreatic secretory granule membrane major glycoprotein GP2 OS=Homo sapiens GN=GP2 PE=2 SV=3 - [GP	8.00	8.00	0.48	0.23
▽	▽	P01266	Thyroglobulin OS=Homo sapiens GN=TG PE=1 SV=5 - [THYG_HUMAN]	1.00	1.00	0.43	0.23
▽	▽	Q08629	Testican-1 OS=Homo sapiens GN=SPOCK1 PE=1 SV=1 - [TICN1_HUMAN]	2.00	2.00	0.34	0.23
▽	▽	P12277	Creatine kinase B-type OS=Homo sapiens GN=CKB PE=1 SV=1 - [KCRB_HUMAN]	9.00	9.00	0.38	0.23
▽	▽	O95183	Vesicle-associated membrane protein 5 OS=Homo sapiens GN=VAMP5 PE=1 SV=1 - [VAMP5_HUMAN]	1.00	1.00	0.49	0.22
▽	▽	O00219	Hyaluronan synthase 3 OS=Homo sapiens GN=HAS3 PE=2 SV=3 - [HAS3_HUMAN]	1.00	1.00	0.46	0.22
▽	▽	Q9NQ84	G-protein coupled receptor family C group 5 member C OS=Homo sapiens GN=GPRC5C PE=1 SV=2 - [GPC5C	7.00	7.00	0.45	0.22
▽	▽	Q9Y3R5	Protein dopey-2 OS=Homo sapiens GN=DOPEY2 PE=1 SV=4 - [DOP2_HUMAN]	3.00	3.00	0.38	0.22
▽	▽	Q8IYJ3	Synaptotagmin-like protein 1 OS=Homo sapiens GN=SYTL1 PE=1 SV=1 - [SYTL1_HUMAN]	1.00	1.00	0.29	0.21
▽	▽	Q9BTM9	Ubiquitin-related modifier 1 homolog OS=Homo sapiens GN=URM1 PE=1 SV=1 - [URM1_HUMAN]	2.00	2.00	0.22	0.21
▽	▽	Q9UBV8	Peflin OS=Homo sapiens GN=PEF1 PE=1 SV=1 - [PEF1_HUMAN]	3.00	3.00	0.46	0.20
▽	▽	P20138	Myeloid cell surface antigen CD33 OS=Homo sapiens GN=CD33 PE=1 SV=2 - [CD33_HUMAN]	1.00	1.00	0.49	0.20
▽	▽	P06870	Kallikrein-1 OS=Homo sapiens GN=KLK1 PE=1 SV=2 - [KLK1_HUMAN]	9.00	9.00	0.47	0.19
▽	▽	P15309	Prostatic acid phosphatase OS=Homo sapiens GN=ACPP PE=1 SV=3 - [PPAP_HUMAN]	12.00	12.00	0.31	0.19
▽	▽	P28907	ADP-ribosyl cyclase 1 OS=Homo sapiens GN=CD38 PE=1 SV=2 - [CD38_HUMAN]	2.00	2.00	0.23	0.19
▽	▽	P51159	Ras-related protein Rab-27A OS=Homo sapiens GN=RAB27A PE=1 SV=3 - [RB27A_HUMAN]	2.00	3.00	0.29	0.19
▽	▽	Q9H9K5	Uncharacterized protein LP9056 OS=Homo sapiens GN=LP9056 PE=2 SV=1 - [L9056_HUMAN]	1.00	1.00	0.38	0.19
▽	▽	P24855	Deoxyribonuclease-1 OS=Homo sapiens GN=DNASE1 PE=1 SV=1 - [DNAS1_HUMAN]	8.00	8.00	0.42	0.19
▽	▽	Q9Y277	Voltage-dependent anion-selective channel protein 3 OS=Homo sapiens GN=VDAC3 PE=1 SV=1 - [VDAC3_H	1.00	1.00	0.12	0.18
▽	▽	Q9H1C7	UPF0467 protein C5orf32 OS=Homo sapiens GN=C5orf32 PE=2 SV=1 - [CE032_HUMAN]	1.00	1.00	0.39	0.18

▽	▽	Q96PX8	SLIT and NTRK-like protein 1 OS=Homo sapiens GN=SLITRK1 PE=1 SV=2 - [SLIK1_HUMAN]	2.00	2.00	0.33	0.18
▽	▽	Q9H0B8	Cysteine-rich secretory protein LCCL domain-containing 2 OS=Homo sapiens GN=CRISPLD2 PE=1 SV=1 - [C	5.00	6.00	0.31	0.18
▽	▽	P08118	Beta-microseminoprotein OS=Homo sapiens GN=MSMB PE=1 SV=1 - [MSMB_HUMAN]	2.00	2.00	0.26	0.18
▽	▽	O14494	Lipid phosphate phosphohydrolase 1 OS=Homo sapiens GN=PPAP2A PE=1 SV=1 - [LPP1_HUMAN]	3.00	3.00	0.42	0.17
▽	▽	Q99732	Lipopolysaccharide-induced tumor necrosis factor-alpha factor OS=Homo sapiens GN=LITAF PE=1 SV=2 - [L	1.00	1.00	0.35	0.17
▽	▽	Q16348	Solute carrier family 15 member 2 OS=Homo sapiens GN=SLC15A2 PE=2 SV=2 - [S15A2_HUMAN]	1.00	1.00	0.35	0.17
▽	▽	P21439	Multidrug resistance protein 3 OS=Homo sapiens GN=ABCB4 PE=1 SV=2 - [MDR3_HUMAN]	1.00	1.00	0.36	0.17
▽	▽	Q9NZH0	G-protein coupled receptor family C group 5 member B OS=Homo sapiens GN=GPRC5B PE=2 SV=2 - [GPC5I	2.00	2.00	0.43	0.17
▽	▽	P19835	Bile salt-activated lipase OS=Homo sapiens GN=CEL PE=1 SV=3 - [CEL_HUMAN]	17.00	17.00	0.42	0.17
▽	▽	Q9UBC9	Small proline-rich protein 3 OS=Homo sapiens GN=SPRR3 PE=1 SV=2 - [SPRR3_HUMAN]	11.00	11.00	0.42	0.16
▽	▽	O15393	Transmembrane protease serine 2 OS=Homo sapiens GN=TMPRSS2 PE=1 SV=3 - [TMPS2_HUMAN]	4.00	4.00	0.41	0.16
▽	▽	P14410	Sucrase-isomaltase, intestinal OS=Homo sapiens GN=SI PE=1 SV=5 - [SUIS_HUMAN]	1.00	2.00	0.27	0.15
▽	▽	P01225	Follitropin subunit beta OS=Homo sapiens GN=FSHB PE=1 SV=2 - [FSHB_HUMAN]	2.00	2.00	0.44	0.15
▽	▽	P28906	Hematopoietic progenitor cell antigen CD34 OS=Homo sapiens GN=CD34 PE=1 SV=2 - [CD34_HUMAN]	1.00	1.00	0.43	0.15
▽	▽	O43653	Prostate stem cell antigen OS=Homo sapiens GN=PSCA PE=1 SV=1 - [PSCA_HUMAN]	1.00	1.00	0.06	0.15
▽	▽	O15173	Membrane-associated progesterone receptor component 2 OS=Homo sapiens GN=PGRMC2 PE=1 SV=1 - [PGR	1.00	2.00	0.25	0.13
▽	▽	Q9UBG3	Cornulin OS=Homo sapiens GN=CRNN PE=1 SV=1 - [CRNN_HUMAN]	4.00	4.00	0.12	0.13
▽	▽	Q9H159	Cadherin-19 OS=Homo sapiens GN=CDH19 PE=2 SV=1 - [CAD19_HUMAN]	1.00	1.00	0.44	0.12
▽	▽	O95460	Matrilin-4 OS=Homo sapiens GN=MATN4 PE=1 SV=3 - [MATN4_HUMAN]	2.00	2.00	0.20	0.11
▽	▽	Q3L8U1	Chromodomain-helicase-DNA-binding protein 9 OS=Homo sapiens GN=CHD9 PE=1 SV=2 - [CHD9_HUMAN	1.00	1.00	0.37	0.10
		**Down-Regulated Proteins in any one Subgroups (428)**					
▽		P02792	Ferritin light chain OS=Homo sapiens GN=FTL PE=1 SV=2 - [FRIL_HUMAN]	7.00	7.00	0.43	1.68
▽		P08779	Keratin, type I cytoskeletal 16 OS=Homo sapiens GN=KRT16 PE=1 SV=4 - [K1C16_HUMAN]	2.00	8.00	0.38	1.61
▽		Q93088	Betaine--homocysteine S-methyltransferase 1 OS=Homo sapiens GN=BHMT PE=1 SV=2 - [BHMT1_HUMAN	10.00	13.00	0.43	1.48
▽		O75891	10-formyltetrahydrofolate dehydrogenase OS=Homo sapiens GN=ALDH1L1 PE=1 SV=2 - [FTHFD_HUMAN]	4.00	4.00	0.44	1.37
▽		Q9UKL6	Phosphatidylcholine transfer protein OS=Homo sapiens GN=PCTP PE=1 SV=1 - [PPCT_HUMAN]	1.00	1.00	0.48	1.35
▽		P61925	cAMP-dependent protein kinase inhibitor alpha OS=Homo sapiens GN=PKIA PE=1 SV=2 - [IPKA_HUMAN]	1.00	1.00	0.42	1.30
▽		P20711	Aromatic-L-amino-acid decarboxylase OS=Homo sapiens GN=DDC PE=1 SV=1 - [DDC_HUMAN]	5.00	5.00	0.40	1.15
▽		Q15020	Squamous cell carcinoma antigen recognized by T-cells 3 OS=Homo sapiens GN=SART3 PE=1 SV=1 - [SART	2.00	2.00	0.49	1.15
▽		P19971	Thymidine phosphorylase OS=Homo sapiens GN=TYMP PE=1 SV=2 - [TYPH_HUMAN]	2.00	2.00	0.29	1.15
▽		P49368	T-complex protein 1 subunit gamma OS=Homo sapiens GN=CCT3 PE=1 SV=4 - [TCPG_HUMAN]	1.00	1.00	0.49	0.97
▽		Q14117	Dihydropyrimidinase OS=Homo sapiens GN=DPYS PE=1 SV=1 - [DPYS_HUMAN]	1.00	1.00	0.48	0.93
▽		Q92597	Protein NDRG1 OS=Homo sapiens GN=NDRG1 PE=1 SV=1 - [NDRG1_HUMAN]	3.00	3.00	0.45	0.92
▽		O75874	Isocitrate dehydrogenase [NADP] cytoplasmic OS=Homo sapiens GN=IDH1 PE=1 SV=2 - [IDHC_HUMAN]	14.00	14.00	0.44	0.77
▽		P00352	Retinal dehydrogenase 1 OS=Homo sapiens GN=ALDH1A1 PE=1 SV=2 - [AL1A1_HUMAN]	14.00	15.00	0.48	0.77
▽		P35558	Phosphoenolpyruvate carboxykinase, cytosolic [GTP] OS=Homo sapiens GN=PCK1 PE=1 SV=3 - [PCKGC_HU	3.00	3.00	0.47	0.71

▽		A0AV96	RNA-binding protein 47 OS=Homo sapiens GN=RBM47 PE=1 SV=1 - [RBM47_HUMAN]	1.00	1.00	0.49	0.70
▽		Q8IZP2	Putative protein FAM10A4 OS=Homo sapiens GN=FAM10A4 PE=5 SV=1 - [F10A4_HUMAN]	3.00	3.00	0.48	0.66
▽		Q9H477	Ribokinase OS=Homo sapiens GN=RBKS PE=1 SV=1 - [RBSK_HUMAN]	2.00	2.00	0.45	0.66
▽		P00568	Adenylate kinase isoenzyme 1 OS=Homo sapiens GN=AK1 PE=1 SV=3 - [KAD1_HUMAN]	2.00	2.00	0.42	0.62
▽		O75348	V-type proton ATPase subunit G 1 OS=Homo sapiens GN=ATP6V1G1 PE=1 SV=3 - [VATG1_HUMAN]	2.00	2.00	0.47	0.61
▽		P54803	Galactocerebrosidase OS=Homo sapiens GN=GALC PE=1 SV=2 - [GALC_HUMAN]	2.00	2.00	0.47	0.59
▽		Q96HF1	Secreted frizzled-related protein 2 OS=Homo sapiens GN=SFRP2 PE=1 SV=2 - [SFRP2_HUMAN]	1.00	1.00	0.49	0.58
▽		Q96I24	Far upstream element-binding protein 3 OS=Homo sapiens GN=FUBP3 PE=1 SV=2 - [FUBP3_HUMAN]	1.00	1.00	0.41	0.58
▽		Q9UK23	N-acetylglucosamine-1-phosphodiester alpha-N-acetylglucosaminidase OS=Homo sapiens GN=NAGPA PE=2 S	1.00	1.00	0.44	0.58
▽		P05937	Calbindin OS=Homo sapiens GN=CALB1 PE=1 SV=2 - [CALB1_HUMAN]	7.00	7.00	0.42	0.58
▽		Q9BZQ8	Protein Niban OS=Homo sapiens GN=FAM129A PE=1 SV=1 - [NIBAN_HUMAN]	1.00	1.00	0.43	0.56
▽		Q00796	Sorbitol dehydrogenase OS=Homo sapiens GN=SORD PE=1 SV=4 - [DHSO_HUMAN]	9.00	9.00	0.44	0.55
▽		P35270	Sepiapterin reductase OS=Homo sapiens GN=SPR PE=1 SV=1 - [SPRE_HUMAN]	1.00	1.00	0.40	0.55
▽		O43451	Maltase-glucoamylase, intestinal OS=Homo sapiens GN=MGAM PE=1 SV=5 - [MGA_HUMAN]	32.00	33.00	0.50	0.54
▽		P27348	14-3-3 protein theta OS=Homo sapiens GN=YWHAQ PE=1 SV=1 - [1433T_HUMAN]	1.00	4.00	0.45	0.54
▽		Q8WW52	Protein FAM151A OS=Homo sapiens GN=FAM151A PE=2 SV=2 - [F151A_HUMAN]	4.00	4.00	0.32	0.53
▽		O60294	Leucine carboxyl methyltransferase 2 OS=Homo sapiens GN=LCMT2 PE=1 SV=3 - [LCMT2_HUMAN]	1.00	1.00	0.43	0.52
▽		Q9BWD1	Acetyl-CoA acetyltransferase, cytosolic OS=Homo sapiens GN=ACAT2 PE=1 SV=2 - [THIC_HUMAN]	1.00	1.00	0.46	0.52
▽		Q9H6S3	Epidermal growth factor receptor kinase substrate 8-like protein 2 OS=Homo sapiens GN=EPS8L2 PE=1 SV=2	6.00	6.00	0.47	0.52
▽		P35908	Keratin, type II cytoskeletal 2 epidermal OS=Homo sapiens GN=KRT2 PE=1 SV=2 - [K22E_HUMAN]	10.00	15.00	0.37	0.50
▽		P68402	Platelet-activating factor acetylhydrolase IB subunit beta OS=Homo sapiens GN=PAFAH1B2 PE=1 SV=1 - [PA	1.00	1.00	0.43	0.50
▽		P19801	Amiloride-sensitive amine oxidase [copper-containing] OS=Homo sapiens GN=ABP1 PE=1 SV=4 - [ABP1_HU	6.00	6.00	0.45	0.50
▽		Q8N335	Glycerol-3-phosphate dehydrogenase 1-like protein OS=Homo sapiens GN=GPD1L PE=1 SV=1 - [GPD1L_HU	2.00	2.00	0.37	0.50
▽		P16234	Alpha-type platelet-derived growth factor receptor OS=Homo sapiens GN=PDGFRA PE=1 SV=1 - [PGFRA_HU	1.00	1.00	0.46	
	▽	Q06418	Tyrosine-protein kinase receptor TYRO3 OS=Homo sapiens GN=TYRO3 PE=1 SV=1 - [TYRO3_HUMAN]	5.00	5.00	0.86	0.50
	▽	O75131	Copine-3 OS=Homo sapiens GN=CPNE3 PE=1 SV=1 - [CPNE3_HUMAN]	8.00	9.00	0.90	0.50
	▽	Q9BRK3	Matrix-remodeling-associated protein 8 OS=Homo sapiens GN=MXRA8 PE=1 SV=1 - [MXRA8_HUMAN]	7.00	7.00	1.03	0.50
	▽	P13598	Intercellular adhesion molecule 2 OS=Homo sapiens GN=ICAM2 PE=1 SV=2 - [ICAM2_HUMAN]	4.00	4.00	1.15	0.50
	▽	Q9HBG4	V-type proton ATPase 116 kDa subunit a isoform 4 OS=Homo sapiens GN=ATP6V0A4 PE=1 SV=1 - [VPP4_H	1.00	1.00	0.58	0.50
	▽	Q9H2A7	C-X-C motif chemokine 16 OS=Homo sapiens GN=CXCL16 PE=2 SV=4 - [CXL16_HUMAN]	2.00	2.00	0.85	0.50
	▽	Q1EHB4	Sodium-coupled monocarboxylate transporter 2 OS=Homo sapiens GN=SLC5A12 PE=2 SV=2 - [SC5AC_HUM	1.00	1.00	0.66	0.50
	▽	Q9BXP8	Pappalysin-2 OS=Homo sapiens GN=PAPPA2 PE=1 SV=4 - [PAPP2_HUMAN]	35.00	35.00	1.26	0.50
	▽	Q6W4X9	Mucin-6 OS=Homo sapiens GN=MUC6 PE=1 SV=2 - [MUC6_HUMAN]	2.00	2.00	0.98	0.50
	▽	Q96NY8	Poliovirus receptor-related protein 4 OS=Homo sapiens GN=PVRL4 PE=1 SV=1 - [PVRL4_HUMAN]	5.00	5.00	1.08	0.49
	▽	P25940	Collagen alpha-3(V) chain OS=Homo sapiens GN=COL5A3 PE=1 SV=3 - [CO5A3_HUMAN]	5.00	5.00	0.85	0.49
	▽	Q9H3Z4	DnaJ homolog subfamily C member 5 OS=Homo sapiens GN=DNAJC5 PE=1 SV=1 - [DNJC5_HUMAN]	1.00	1.00	0.72	0.49

	▽	A6NGN9	IgLON family member 5 OS=Homo sapiens GN=IGLON5 PE=2 SV=4 - [IGLO5_HUMAN]	2.00	2.00	1.07	0.49
	▽	P21266	Glutathione S-transferase Mu 3 OS=Homo sapiens GN=GSTM3 PE=1 SV=3 - [GSTM3_HUMAN]	11.00	11.00	0.60	0.49
	▽	Q16819	Meprin A subunit alpha OS=Homo sapiens GN=MEP1A PE=2 SV=2 - [MEP1A_HUMAN]	4.00	4.00	1.02	0.49
	▽	Q9BRT3	Uncharacterized protein C17orf37 OS=Homo sapiens GN=C17orf37 PE=2 SV=1 - [CQ037_HUMAN]	3.00	3.00	0.75	0.49
	▽	P63000	Ras-related C3 botulinum toxin substrate 1 OS=Homo sapiens GN=RAC1 PE=1 SV=1 - [RAC1_HUMAN]	4.00	5.00	0.61	0.49
	▽	O60939	Sodium channel subunit beta-2 OS=Homo sapiens GN=SCN2B PE=1 SV=1 - [SCN2B_HUMAN]	1.00	1.00	1.58	0.49
	▽	P05090	Apolipoprotein D OS=Homo sapiens GN=APOD PE=1 SV=1 - [APOD_HUMAN]	10.00	10.00	1.11	0.49
	▽	Q16827	Receptor-type tyrosine-protein phosphatase O OS=Homo sapiens GN=PTPRO PE=1 SV=2 - [PTPRO_HUMAN	1.00	1.00	0.86	0.49
	▽	P54753	Ephrin type-B receptor 3 OS=Homo sapiens GN=EPHB3 PE=1 SV=2 - [EPHB3_HUMAN]	5.00	5.00	0.77	0.48
	▽	Q9NY97	UDP-GlcNAc:betaGal beta-1,3-N-acetylglucosaminyltransferase 2 OS=Homo sapiens GN=B3GNT2 PE=1 SV=	5.00	5.00	0.82	0.48
	▽	P55285	Cadherin-6 OS=Homo sapiens GN=CDH6 PE=1 SV=1 - [CADH6_HUMAN]	5.00	5.00	0.74	0.48
	▽	P41214	Ligatin OS=Homo sapiens GN=LGTN PE=1 SV=3 - [LIGA_HUMAN]	1.00	1.00	0.60	0.48
	▽	Q68D85	Putative Ig-like domain-containing protein DKFZp686O24166/DKFZp686I21167 OS=Homo sapiens PE=2 SV=	3.00	3.00	0.69	0.48
	▽	Q13332	Receptor-type tyrosine-protein phosphatase S OS=Homo sapiens GN=PTPRS PE=1 SV=2 - [PTPRS_HUMAN]	16.00	16.00	0.90	0.48
	▽	Q9H8L6	Multimerin-2 OS=Homo sapiens GN=MMRN2 PE=1 SV=2 - [MMRN2_HUMAN]	6.00	6.00	0.65	0.48
	▽	P16401	Histone H1.5 OS=Homo sapiens GN=HIST1H1B PE=1 SV=3 - [H15_HUMAN]	2.00	2.00	0.63	0.48
	▽	Q5D862	Filaggrin-2 OS=Homo sapiens GN=FLG2 PE=1 SV=1 - [FILA2_HUMAN]	3.00	3.00	1.04	0.48
	▽	Q9BRG1	Vacuolar protein-sorting-associated protein 25 OS=Homo sapiens GN=VPS25 PE=1 SV=1 - [VPS25_HUMAN]	2.00	2.00	0.77	0.48
	▽	Q9HCM3	UPF0606 protein KIAA1549 OS=Homo sapiens GN=KIAA1549 PE=1 SV=3 - [K1549_HUMAN]	2.00	2.00	0.83	0.48
	▽	P13645	Keratin, type I cytoskeletal 10 OS=Homo sapiens GN=KRT10 PE=1 SV=6 - [K1C10_HUMAN]	17.00	20.00	0.51	0.48
	▽	Q96RW7	Hemicentin-1 OS=Homo sapiens GN=HMCN1 PE=1 SV=2 - [HMCN1_HUMAN]	8.00	8.00	0.82	0.48
	▽	O00144	Frizzled-9 OS=Homo sapiens GN=FZD9 PE=2 SV=1 - [FZD9_HUMAN]	1.00	1.00	0.88	0.48
	▽	Q92956	Tumor necrosis factor receptor superfamily member 14 OS=Homo sapiens GN=TNFRSF14 PE=1 SV=3 - [TNR	4.00	4.00	1.01	0.48
	▽	P36957	Dihydrolipoyllysine-residue succinyltransferase component of 2-oxoglutarate dehydrogenase complex, mitochon	4.00	4.00	0.67	0.48
	▽	P07942	Laminin subunit beta-1 OS=Homo sapiens GN=LAMB1 PE=1 SV=1 - [LAMB1_HUMAN]	6.00	7.00	0.96	0.48
	▽	Q6V0I7	Protocadherin Fat 4 OS=Homo sapiens GN=FAT4 PE=2 SV=2 - [FAT4_HUMAN]	16.00	16.00	0.72	0.48
	▽	Q08334	Interleukin-10 receptor subunit beta OS=Homo sapiens GN=IL10RB PE=1 SV=2 - [I10R2_HUMAN]	2.00	2.00	1.00	0.47
	▽	Q96SL1	Disrupted in renal carcinoma protein 2 OS=Homo sapiens GN=DIRC2 PE=2 SV=1 - [DIRC2_HUMAN]	1.00	1.00	0.79	0.47
	▽	Q5VSG8	Glycoprotein endo-alpha-1,2-mannosidase-like protein OS=Homo sapiens GN=MANEAL PE=2 SV=1 - [MANI	1.00	1.00	0.83	0.47
	▽	P02776	Platelet factor 4 OS=Homo sapiens GN=PF4 PE=1 SV=2 - [PLF4_HUMAN]	2.00	2.00	0.98	0.47
	▽	O00322	Uroplakin-1a OS=Homo sapiens GN=UPK1A PE=2 SV=1 - [UPK1A_HUMAN]	4.00	4.00	0.91	0.47
	▽	P69849	Nodal modulator 3 OS=Homo sapiens GN=NOMO3 PE=2 SV=2 - [NOMO3_HUMAN]	1.00	1.00	0.90	0.47
	▽	O75339	Cartilage intermediate layer protein 1 OS=Homo sapiens GN=CILP PE=1 SV=3 - [CILP1_HUMAN]	2.00	2.00	0.65	0.47
	▽	Q9NY25	C-type lectin domain family 5 member A OS=Homo sapiens GN=CLEC5A PE=1 SV=1 - [CLC5A_HUMAN]	2.00	2.00	0.79	0.47
	▽	P31150	Rab GDP dissociation inhibitor alpha OS=Homo sapiens GN=GDI1 PE=1 SV=2 - [GDIA_HUMAN]	2.00	5.00	0.68	0.47
	▽	P07320	Gamma-crystallin D OS=Homo sapiens GN=CRYGD PE=1 SV=3 - [CRGD_HUMAN]	1.00	1.00	0.65	0.47

	▽	Q9UN37	Vacuolar protein sorting-associated protein 4A OS=Homo sapiens GN=VPS4A PE=1 SV=1 - [VPS4A_HUMAN	7.00	9.00	0.61	0.47
	▽	Q9H190	Syntenin-2 OS=Homo sapiens GN=SDCBP2 PE=2 SV=2 - [SDCB2_HUMAN]	1.00	1.00	0.88	0.47
	▽	P61421	V-type proton ATPase subunit d 1 OS=Homo sapiens GN=ATP6V0D1 PE=1 SV=1 - [VA0D1_HUMAN]	1.00	1.00	0.70	0.47
	▽	O43586	Proline-serine-threonine phosphatase-interacting protein 1 OS=Homo sapiens GN=PSTPIP1 PE=1 SV=1 - [PPIF	1.00	1.00	0.57	0.47
	▽	P36896	Activin receptor type-1B OS=Homo sapiens GN=ACVR1B PE=1 SV=1 - [ACV1B_HUMAN]	1.00	1.00	1.00	0.46
	▽	Q9GZX9	Twisted gastrulation protein homolog 1 OS=Homo sapiens GN=TWSG1 PE=2 SV=1 - [TWSG1_HUMAN]	5.00	5.00	0.99	0.46
	▽	P34059	N-acetylgalactosamine-6-sulfatase OS=Homo sapiens GN=GALNS PE=1 SV=1 - [GALNS_HUMAN]	10.00	10.00	0.61	0.46
	▽	P60953	Cell division control protein 42 homolog OS=Homo sapiens GN=CDC42 PE=1 SV=1 - [CDC42_HUMAN]	4.00	5.00	0.50	0.46
	▽	O60494	Cubilin OS=Homo sapiens GN=CUBN PE=1 SV=4 - [CUBN_HUMAN]	63.00	63.00	0.68	0.46
	▽	P63092	Guanine nucleotide-binding protein G(s) subunit alpha isoforms short OS=Homo sapiens GN=GNAS PE=1 SV=	6.00	6.00	0.65	0.46
	▽	Q13449	Limbic system-associated membrane protein OS=Homo sapiens GN=LSAMP PE=1 SV=2 - [LSAMP_HUMAN	3.00	3.00	0.70	0.46
	▽	Q15111	Inactive phospholipase C-like protein 1 OS=Homo sapiens GN=PLCL1 PE=1 SV=3 - [PLCL1_HUMAN]	2.00	2.00	0.55	0.46
	▽	Q13162	Peroxiredoxin-4 OS=Homo sapiens GN=PRDX4 PE=1 SV=1 - [PRDX4_HUMAN]	1.00	2.00	0.83	0.46
	▽	P53801	Pituitary tumor-transforming gene 1 protein-interacting protein OS=Homo sapiens GN=PTTG1IP PE=1 SV=1 -	2.00	2.00	0.76	0.46
	▽	O60888	Protein CutA OS=Homo sapiens GN=CUTA PE=1 SV=2 - [CUTA_HUMAN]	6.00	6.00	0.67	0.46
	▽	Q9UJ99	Cadherin-22 OS=Homo sapiens GN=CDH22 PE=2 SV=2 - [CAD22_HUMAN]	1.00	1.00	0.70	0.46
	▽	Q05707	Collagen alpha-1(XIV) chain OS=Homo sapiens GN=COL14A1 PE=1 SV=3 - [COEA1_HUMAN]	9.00	9.00	0.93	0.46
	▽	Q9UJ72	Annexin A10 OS=Homo sapiens GN=ANXA10 PE=1 SV=3 - [ANX10_HUMAN]	1.00	1.00	1.43	0.46
	▽	Q8TB96	T-cell immunomodulatory protein OS=Homo sapiens GN=ITFG1 PE=1 SV=1 - [TIP_HUMAN]	4.00	4.00	0.69	0.46
	▽	Q96A22	Uncharacterized protein C11orf52 OS=Homo sapiens GN=C11orf52 PE=1 SV=2 - [CK052_HUMAN]	1.00	1.00	0.72	0.46
	▽	Q8TDQ1	CMRF35-like molecule 1 OS=Homo sapiens GN=CD300LF PE=1 SV=3 - [CLM1_HUMAN]	2.00	2.00	0.82	0.46
	▽	P35241	Radixin OS=Homo sapiens GN=RDX PE=1 SV=1 - [RADI_HUMAN]	3.00	8.00	0.69	0.46
	▽	P10253	Lysosomal alpha-glucosidase OS=Homo sapiens GN=GAA PE=1 SV=3 - [LYAG_HUMAN]	29.00	29.00	0.88	0.46
	▽	Q7Z7M0	Multiple epidermal growth factor-like domains protein 8 OS=Homo sapiens GN=MEGF8 PE=1 SV=2 - [MEGF8	18.00	18.00	0.76	0.46
	▽	P09603	Macrophage colony-stimulating factor 1 OS=Homo sapiens GN=CSF1 PE=1 SV=1 - [CSF1_HUMAN]	11.00	11.00	0.80	0.45
	▽	Q8IWU5	Extracellular sulfatase Sulf-2 OS=Homo sapiens GN=SULF2 PE=1 SV=1 - [SULF2_HUMAN]	3.00	3.00	0.79	0.45
	▽	Q9BUN1	Uncharacterized protein C1orf56 OS=Homo sapiens GN=C1orf56 PE=1 SV=1 - [CA056_HUMAN]	1.00	1.00	0.67	0.45
	▽	Q96QR1	Secretoglobin family 3A member 1 OS=Homo sapiens GN=SCGB3A1 PE=1 SV=2 - [SG3A1_HUMAN]	1.00	1.00	0.50	0.45
	▽	P0C7U0	Extracellular leucine-rich repeat and fibronectin type-III domain-containing protein 1 OS=Homo sapiens GN=EI	1.00	1.00	0.93	0.45
	▽	P24821	Tenascin OS=Homo sapiens GN=TNC PE=1 SV=3 - [TENA_HUMAN]	29.00	29.00	0.86	0.45
	▽	Q08345	Epithelial discoidin domain-containing receptor 1 OS=Homo sapiens GN=DDR1 PE=1 SV=1 - [DDR1_HUMAI	5.00	5.00	0.93	0.45
	▽	P62879	Guanine nucleotide-binding protein G(I)/G(S)/G(T) subunit beta-2 OS=Homo sapiens GN=GNB2 PE=1 SV=3 -	5.00	5.00	0.65	0.45
	▽	Q06481	Amyloid-like protein 2 OS=Homo sapiens GN=APLP2 PE=1 SV=2 - [APLP2_HUMAN]	12.00	12.00	0.65	0.45
	▽	P01130	Low-density lipoprotein receptor OS=Homo sapiens GN=LDLR PE=1 SV=1 - [LDLR_HUMAN]	2.00	2.00	1.16	0.45
	▽	A8MVW5	HEPACAM family member 2 OS=Homo sapiens GN=HEPACAM2 PE=2 SV=1 - [HECA2_HUMAN]	1.00	1.00	0.98	0.45
	▽	Q8TAB3	Protocadherin-19 OS=Homo sapiens GN=PCDH19 PE=1 SV=3 - [PCD19_HUMAN]	2.00	2.00	0.73	0.45

	▽	P32418	Sodium/calcium exchanger 1 OS=Homo sapiens GN=SLC8A1 PE=1 SV=3 - [NAC1_HUMAN]	3.00	3.00	0.65	0.45
	▽	O00526	Uroplakin-2 OS=Homo sapiens GN=UPK2 PE=2 SV=2 - [UPK2_HUMAN]	4.00	4.00	0.78	0.44
	▽	P54289	Voltage-dependent calcium channel subunit alpha-2/delta-1 OS=Homo sapiens GN=CACNA2D1 PE=1 SV=3 -	4.00	4.00	0.73	0.44
	▽	P08519	Apolipoprotein(a) OS=Homo sapiens GN=LPA PE=1 SV=1 - [APOA_HUMAN]	5.00	5.00	0.98	0.44
	▽	P21802	Fibroblast growth factor receptor 2 OS=Homo sapiens GN=FGFR2 PE=1 SV=1 - [FGFR2_HUMAN]	4.00	4.00	0.71	0.44
	▽	P28827	Receptor-type tyrosine-protein phosphatase mu OS=Homo sapiens GN=PTPRM PE=1 SV=2 - [PTPRM_HUMA	1.00	1.00	0.76	0.44
	▽	Q9H461	Frizzled-8 OS=Homo sapiens GN=FZD8 PE=2 SV=1 - [FZD8_HUMAN]	1.00	1.00	0.64	0.44
	▽	P22304	Iduronate 2-sulfatase OS=Homo sapiens GN=IDS PE=1 SV=1 - [IDS_HUMAN]	6.00	6.00	0.67	0.44
	▽	P25189	Myelin protein P0 OS=Homo sapiens GN=MPZ PE=1 SV=1 - [MYP0_HUMAN]	3.00	3.00	1.15	0.44
	▽	O00462	Beta-mannosidase OS=Homo sapiens GN=MANBA PE=1 SV=3 - [MANBA_HUMAN]	17.00	17.00	0.78	0.44
	▽	P51688	N-sulphoglucosamine sulphohydrolase OS=Homo sapiens GN=SGSH PE=1 SV=1 - [SPHM_HUMAN]	9.00	9.00	0.51	0.44
	▽	Q9BY67	Cell adhesion molecule 1 OS=Homo sapiens GN=CADM1 PE=1 SV=2 - [CADM1_HUMAN]	10.00	10.00	0.69	0.44
	▽	P32004	Neural cell adhesion molecule L1 OS=Homo sapiens GN=L1CAM PE=1 SV=2 - [L1CAM_HUMAN]	11.00	11.00	0.81	0.44
	▽	O94910	Latrophilin-1 OS=Homo sapiens GN=LPHN1 PE=1 SV=1 - [LPHN1_HUMAN]	7.00	7.00	0.85	0.44
	▽	P39059	Collagen alpha-1(XV) chain OS=Homo sapiens GN=COL15A1 PE=1 SV=2 - [COFA1_HUMAN]	13.00	13.00	0.79	0.44
	▽	Q9BXI6	TBC1 domain family member 10A OS=Homo sapiens GN=TBC1D10A PE=1 SV=1 - [TB10A_HUMAN]	1.00	1.00	0.51	0.43
	▽	Q9BQI6	Ankyrin repeat domain-containing protein 32 OS=Homo sapiens GN=ANKRD32 PE=1 SV=2 - [ANR32_HUM	1.00	1.00	0.76	0.43
	▽	P23284	Peptidyl-prolyl cis-trans isomerase B OS=Homo sapiens GN=PPIB PE=1 SV=2 - [PPIB_HUMAN]	5.00	5.00	0.51	0.43
	▽	P12259	Coagulation factor V OS=Homo sapiens GN=F5 PE=1 SV=3 - [FA5_HUMAN]	4.00	4.00	0.76	0.43
	▽	P35555	Fibrillin-1 OS=Homo sapiens GN=FBN1 PE=1 SV=2 - [FBN1_HUMAN]	38.00	38.00	0.89	0.43
	▽	O00182	Galectin-9 OS=Homo sapiens GN=LGALS9 PE=1 SV=2 - [LEG9_HUMAN]	4.00	4.00	0.73	0.43
	▽	Q6EMK4	Vasorin OS=Homo sapiens GN=VASN PE=1 SV=1 - [VASN_HUMAN]	9.00	9.00	0.77	0.43
	▽	P53985	Monocarboxylate transporter 1 OS=Homo sapiens GN=SLC16A1 PE=1 SV=2 - [MOT1_HUMAN]	1.00	1.00	0.81	0.43
	▽	Q5SZK8	FRAS1-related extracellular matrix protein 2 OS=Homo sapiens GN=FREM2 PE=1 SV=2 - [FREM2_HUMAN]	19.00	19.00	0.54	0.43
	▽	O43291	Kunitz-type protease inhibitor 2 OS=Homo sapiens GN=SPINT2 PE=1 SV=2 - [SPIT2_HUMAN]	3.00	3.00	0.88	0.43
	▽	Q6UWH4	Protein FAM198B OS=Homo sapiens GN=FAM198B PE=2 SV=1 - [F198B_HUMAN]	1.00	1.00	0.76	0.43
	▽	Q9UNZ2	NSFL1 cofactor p47 OS=Homo sapiens GN=NSFL1C PE=1 SV=2 - [NSF1C_HUMAN]	1.00	1.00	0.64	0.43
	▽	Q9Y240	C-type lectin domain family 11 member A OS=Homo sapiens GN=CLEC11A PE=1 SV=1 - [CLC11_HUMAN]	1.00	1.00	0.77	0.43
	▽	Q15262	Receptor-type tyrosine-protein phosphatase kappa OS=Homo sapiens GN=PTPRK PE=1 SV=2 - [PTPRK_HUM	1.00	1.00	0.75	0.43
	▽	P61019	Ras-related protein Rab-2A OS=Homo sapiens GN=RAB2A PE=1 SV=1 - [RAB2A_HUMAN]	1.00	1.00	0.57	0.43
	▽	O15484	Calpain-5 OS=Homo sapiens GN=CAPN5 PE=2 SV=2 - [CAN5_HUMAN]	2.00	2.00	0.69	0.42
	▽	Q96FE5	Leucine-rich repeat and immunoglobulin-like domain-containing nogo receptor-interacting protein 1 OS=Homo	1.00	1.00	0.88	0.42
	▽	P27105	Erythrocyte band 7 integral membrane protein OS=Homo sapiens GN=STOM PE=1 SV=3 - [STOM_HUMAN]	9.00	9.00	0.56	0.42
	▽	P13987	CD59 glycoprotein OS=Homo sapiens GN=CD59 PE=1 SV=1 - [CD59_HUMAN]	8.00	8.00	0.93	0.42
	▽	Q16832	Discoidin domain-containing receptor 2 OS=Homo sapiens GN=DDR2 PE=1 SV=2 - [DDR2_HUMAN]	4.00	4.00	0.93	0.42
	▽	Q13591	Semaphorin-5A OS=Homo sapiens GN=SEMA5A PE=1 SV=3 - [SEM5A_HUMAN]	2.00	2.00	0.57	0.42

	▽	Q9UF11	Pleckstrin homology domain-containing family B member 1 OS=Homo sapiens GN=PLEKHB1 PE=1 SV=1 - [F	1.00	1.00	0.65	0.42
	▽	Q96JQ0	Protocadherin-16 OS=Homo sapiens GN=DCHS1 PE=2 SV=1 - [PCD16_HUMAN]	6.00	6.00	0.81	0.42
	▽	Q8WV92	MIT domain-containing protein 1 OS=Homo sapiens GN=MITD1 PE=1 SV=1 - [MITD1_HUMAN]	2.00	2.00	0.67	0.42
	▽	Q9P2M7	Cingulin OS=Homo sapiens GN=CGN PE=1 SV=2 - [CING_HUMAN]	1.00	1.00	1.86	0.41
	▽	Q9NRA2	Sialin OS=Homo sapiens GN=SLC17A5 PE=1 SV=2 - [S17A5_HUMAN]	1.00	1.00	0.56	0.41
	▽	P07355	Annexin A2 OS=Homo sapiens GN=ANXA2 PE=1 SV=2 - [ANXA2_HUMAN]	12.00	12.00	0.63	0.41
	▽	Q13621	Solute carrier family 12 member 1 OS=Homo sapiens GN=SLC12A1 PE=1 SV=2 - [S12A1_HUMAN]	6.00	7.00	0.68	0.41
	▽	Q12794	Hyaluronidase-1 OS=Homo sapiens GN=HYAL1 PE=1 SV=2 - [HYAL1_HUMAN]	7.00	7.00	0.52	0.41
	▽	Q99523	Sortilin OS=Homo sapiens GN=SORT1 PE=1 SV=3 - [SORT_HUMAN]	4.00	4.00	0.80	0.41
	▽	Q9H756	Leucine-rich repeat-containing protein 19 OS=Homo sapiens GN=LRRC19 PE=2 SV=1 - [LRC19_HUMAN]	2.00	2.00	0.81	0.41
	▽	P26718	NKG2-D type II integral membrane protein OS=Homo sapiens GN=KLRK1 PE=1 SV=1 - [NKG2D_HUMAN]	1.00	1.00	0.63	0.41
	▽	Q15746	Myosin light chain kinase, smooth muscle OS=Homo sapiens GN=MYLK PE=1 SV=3 - [MYLK_HUMAN]	2.00	2.00	0.53	0.41
	▽	P01589	Interleukin-2 receptor subunit alpha OS=Homo sapiens GN=IL2RA PE=1 SV=1 - [IL2RA_HUMAN]	3.00	3.00	1.16	0.41
	▽	Q9C0H2	Protein tweety homolog 3 OS=Homo sapiens GN=TTYH3 PE=1 SV=3 - [TTYH3_HUMAN]	3.00	3.00	0.62	0.41
	▽	P19075	Tetraspanin-8 OS=Homo sapiens GN=TSPAN8 PE=1 SV=1 - [TSN8_HUMAN]	2.00	2.00	0.69	0.41
	▽	P05067	Amyloid beta A4 protein OS=Homo sapiens GN=APP PE=1 SV=3 - [A4_HUMAN]	10.00	10.00	0.67	0.41
	▽	Q16651	Prostasin OS=Homo sapiens GN=PRSS8 PE=1 SV=1 - [PRSS8_HUMAN]	3.00	3.00	0.87	0.41
	▽	O00560	Syntenin-1 OS=Homo sapiens GN=SDCBP PE=1 SV=1 - [SDCB1_HUMAN]	6.00	6.00	0.63	0.41
	▽	Q8NFZ8	Cell adhesion molecule 4 OS=Homo sapiens GN=CADM4 PE=1 SV=1 - [CADM4_HUMAN]	7.00	7.00	0.80	0.40
	▽	O75340	Programmed cell death protein 6 OS=Homo sapiens GN=PDCD6 PE=1 SV=1 - [PDCD6_HUMAN]	2.00	2.00	0.57	0.40
	▽	O43852	Calumenin OS=Homo sapiens GN=CALU PE=1 SV=2 - [CALU_HUMAN]	6.00	6.00	0.68	0.40
	▽	Q96J84	Kin of IRRE-like protein 1 OS=Homo sapiens GN=KIRREL PE=1 SV=2 - [KIRR1_HUMAN]	7.00	7.00	0.79	0.40
	▽	Q5VY43	Platelet endothelial aggregation receptor 1 OS=Homo sapiens GN=PEAR1 PE=1 SV=1 - [PEAR1_HUMAN]	2.00	2.00	0.76	0.40
	▽	P11117	Lysosomal acid phosphatase OS=Homo sapiens GN=ACP2 PE=1 SV=3 - [PPAL_HUMAN]	10.00	10.00	0.58	0.40
	▽	P08582	Melanotransferrin OS=Homo sapiens GN=MFI2 PE=1 SV=1 - [TRFM_HUMAN]	6.00	6.00	0.65	0.40
	▽	P61366	Osteocrin OS=Homo sapiens GN=OSTN PE=2 SV=1 - [OSTN_HUMAN]	1.00	1.00	1.16	0.40
	▽	P20916	Myelin-associated glycoprotein OS=Homo sapiens GN=MAG PE=1 SV=1 - [MAG_HUMAN]	2.00	2.00	0.99	0.40
	▽	Q6UW56	Apoptosis-related protein 3 OS=Homo sapiens GN=APR3 PE=1 SV=2 - [APR3_HUMAN]	1.00	1.00	1.05	0.40
	▽	Q6FHJ7	Secreted frizzled-related protein 4 OS=Homo sapiens GN=SFRP4 PE=1 SV=2 - [SFRP4_HUMAN]	3.00	3.00	0.72	0.40
	▽	Q8TDQ0	Hepatitis A virus cellular receptor 2 OS=Homo sapiens GN=HAVCR2 PE=1 SV=2 - [TIMD3_HUMAN]	3.00	3.00	0.92	0.40
	▽	Q8IWA5	Choline transporter-like protein 2 OS=Homo sapiens GN=SLC44A2 PE=1 SV=2 - [CTL2_HUMAN]	6.00	6.00	0.60	0.40
	▽	Q9UN70	Protocadherin gamma-C3 OS=Homo sapiens GN=PCDHGC3 PE=1 SV=1 - [PCDGK_HUMAN]	7.00	7.00	0.73	0.40
	▽	P09525	Annexin A4 OS=Homo sapiens GN=ANXA4 PE=1 SV=4 - [ANXA4_HUMAN]	10.00	10.00	0.64	0.40
	▽	Q96GW7	Brevican core protein OS=Homo sapiens GN=BCAN PE=1 SV=2 - [PGCB_HUMAN]	2.00	2.00	0.88	0.39
	▽	Q9Y4C0	Neurexin-3-alpha OS=Homo sapiens GN=NRXN3 PE=2 SV=4 - [NRX3A_HUMAN]	3.00	3.00	1.00	0.39
	▽	Q96FZ7	Charged multivesicular body protein 6 OS=Homo sapiens GN=CHMP6 PE=1 SV=3 - [CHMP6_HUMAN]	1.00	1.00	0.53	0.39

	▽	O60896	Receptor activity-modifying protein 3 OS=Homo sapiens GN=RAMP3 PE=2 SV=1 - [RAMP3_HUMAN]	1.00	1.00	0.53	0.39
	▽	P11234	Ras-related protein Ral-B OS=Homo sapiens GN=RALB PE=1 SV=1 - [RALB_HUMAN]	3.00	3.00	0.68	0.39
	▽	Q8NFJ5	Retinoic acid-induced protein 3 OS=Homo sapiens GN=GPRC5A PE=1 SV=2 - [RAI3_HUMAN]	2.00	2.00	0.57	0.39
	▽	Q8IZF2	Probable G-protein coupled receptor 116 OS=Homo sapiens GN=GPR116 PE=1 SV=3 - [GP116_HUMAN]	6.00	6.00	0.58	0.39
	▽	P55107	Bone morphogenetic protein 3B OS=Homo sapiens GN=GDF10 PE=1 SV=1 - [BMP3B_HUMAN]	1.00	1.00	0.58	0.39
	▽	P02462	Collagen alpha-1(IV) chain OS=Homo sapiens GN=COL4A1 PE=1 SV=3 - [CO4A1_HUMAN]	1.00	1.00	0.57	0.39
	▽	Q8N3J6	Cell adhesion molecule 2 OS=Homo sapiens GN=CADM2 PE=2 SV=1 - [CADM2_HUMAN]	4.00	4.00	0.88	0.39
	▽	P14384	Carboxypeptidase M OS=Homo sapiens GN=CPM PE=1 SV=2 - [CBPM_HUMAN]	12.00	12.00	0.75	0.39
	▽	Q99715	Collagen alpha-1(XII) chain OS=Homo sapiens GN=COL12A1 PE=1 SV=2 - [COCA1_HUMAN]	25.00	25.00	0.72	0.39
	▽	Q9UPR5	Sodium/calcium exchanger 2 OS=Homo sapiens GN=SLC8A2 PE=2 SV=2 - [NAC2_HUMAN]	2.00	2.00	0.91	0.39
	▽	P61586	Transforming protein RhoA OS=Homo sapiens GN=RHOA PE=1 SV=1 - [RHOA_HUMAN]	6.00	6.00	0.56	0.39
	▽	Q9UBX7	Kallikrein-11 OS=Homo sapiens GN=KLK11 PE=1 SV=2 - [KLK11_HUMAN]	7.00	7.00	0.86	0.39
	▽	P35968	Vascular endothelial growth factor receptor 2 OS=Homo sapiens GN=KDR PE=1 SV=2 - [VGFR2_HUMAN]	1.00	1.00	1.27	0.39
	▽	Q15375	Ephrin type-A receptor 7 OS=Homo sapiens GN=EPHA7 PE=1 SV=3 - [EPHA7_HUMAN]	7.00	7.00	0.81	0.39
	▽	Q13477	Mucosal addressin cell adhesion molecule 1 OS=Homo sapiens GN=MADCAM1 PE=1 SV=2 - [MADCA_HUM	5.00	5.00	0.68	0.39
	▽	P16870	Carboxypeptidase E OS=Homo sapiens GN=CPE PE=1 SV=1 - [CBPE_HUMAN]	18.00	18.00	0.63	0.38
	▽	O75074	Low-density lipoprotein receptor-related protein 3 OS=Homo sapiens GN=LRP3 PE=2 SV=2 - [LRP3_HUMAN	1.00	1.00	0.97	0.38
	▽	A8K7I4	Calcium-activated chloride channel regulator 1 OS=Homo sapiens GN=CLCA1 PE=1 SV=2 - [CLCA1_HUMA	1.00	1.00	0.77	0.38
	▽	Q12860	Contactin-1 OS=Homo sapiens GN=CNTN1 PE=1 SV=1 - [CNTN1_HUMAN]	19.00	19.00	0.67	0.38
	▽	P54710	Sodium/potassium-transporting ATPase subunit gamma OS=Homo sapiens GN=FXYD2 PE=1 SV=3 - [ATNG_	1.00	1.00	1.20	0.38
	▽	P17342	Atrial natriuretic peptide receptor 3 OS=Homo sapiens GN=NPR3 PE=1 SV=2 - [ANPRC_HUMAN]	2.00	2.00	0.61	0.38
	▽	P08294	Extracellular superoxide dismutase [Cu-Zn] OS=Homo sapiens GN=SOD3 PE=1 SV=2 - [SODE_HUMAN]	9.00	9.00	0.66	0.38
	▽	O94991	SLIT and NTRK-like protein 5 OS=Homo sapiens GN=SLITRK5 PE=1 SV=2 - [SLIK5_HUMAN]	2.00	2.00	0.74	0.38
	▽	P11047	Laminin subunit gamma-1 OS=Homo sapiens GN=LAMC1 PE=1 SV=3 - [LAMC1_HUMAN]	8.00	8.00	0.85	0.38
	▽	Q9HCN6	Platelet glycoprotein VI OS=Homo sapiens GN=GP6 PE=1 SV=2 - [GPVI_HUMAN]	6.00	6.00	1.01	0.38
	▽	P23468	Receptor-type tyrosine-protein phosphatase delta OS=Homo sapiens GN=PTPRD PE=1 SV=2 - [PTPRD_HUM	2.00	2.00	0.69	0.38
	▽	Q96DA0	Zymogen granule protein 16 homolog B OS=Homo sapiens GN=ZG16B PE=1 SV=3 - [ZG16B_HUMAN]	6.00	6.00	0.97	0.38
	▽	P08174	Complement decay-accelerating factor OS=Homo sapiens GN=CD55 PE=1 SV=4 - [DAF_HUMAN]	13.00	13.00	1.25	0.38
	▽	O75631	Uroplakin-3a OS=Homo sapiens GN=UPK3A PE=1 SV=3 - [UPK3A_HUMAN]	2.00	2.00	0.92	0.38
	▽	Q13291	Signaling lymphocytic activation molecule OS=Homo sapiens GN=SLAMF1 PE=1 SV=1 - [SLAF1_HUMAN]	1.00	1.00	0.92	0.38
	▽	P04899	Guanine nucleotide-binding protein G(i) subunit alpha-2 OS=Homo sapiens GN=GNAI2 PE=1 SV=3 - [GNAI2_	4.00	5.00	0.68	0.38
	▽	P07476	Involucrin OS=Homo sapiens GN=IVL PE=1 SV=2 - [INVO_HUMAN]	3.00	3.00	0.54	0.38
	▽	P15151	Poliovirus receptor OS=Homo sapiens GN=PVR PE=1 SV=2 - [PVR_HUMAN]	3.00	3.00	0.96	0.38
	▽	O43490	Prominin-1 OS=Homo sapiens GN=PROM1 PE=1 SV=1 - [PROM1_HUMAN]	18.00	18.00	0.85	0.38
	▽	O00533	Neural cell adhesion molecule L1-like protein OS=Homo sapiens GN=CHL1 PE=1 SV=3 - [CHL1_HUMAN]	18.00	18.00	0.87	0.38
	▽	Q99608	Necdin OS=Homo sapiens GN=NDN PE=1 SV=1 - [NECD_HUMAN]	1.00	1.00	0.57	0.37

	▽	P01215	Glycoprotein hormones alpha chain OS=Homo sapiens GN=CGA PE=1 SV=1 - [GLHA_HUMAN]	1.00	1.00	0.92	0.37
	▽	Q92896	Golgi apparatus protein 1 OS=Homo sapiens GN=GLG1 PE=1 SV=2 - [GSLG1_HUMAN]	16.00	16.00	0.70	0.37
	▽	Q6UXB3	Ly6/PLAUR domain-containing protein 2 OS=Homo sapiens GN=LYPD2 PE=2 SV=1 - [LYPD2_HUMAN]	1.00	1.00	0.76	0.37
	▽	O75351	Vacuolar protein sorting-associated protein 4B OS=Homo sapiens GN=VPS4B PE=1 SV=2 - [VPS4B_HUMAN	4.00	6.00	0.61	0.37
	▽	P98155	Very low-density lipoprotein receptor OS=Homo sapiens GN=VLDLR PE=1 SV=1 - [VLDLR_HUMAN]	1.00	1.00	0.59	0.37
	▽	O94856	Neurofascin OS=Homo sapiens GN=NFASC PE=1 SV=4 - [NFASC_HUMAN]	1.00	1.00	0.70	0.37
	▽	P01258	Calcitonin OS=Homo sapiens GN=CALCA PE=1 SV=1 - [CALC_HUMAN]	3.00	3.00	0.70	0.37
	▽	P04180	Phosphatidylcholine-sterol acyltransferase OS=Homo sapiens GN=LCAT PE=1 SV=1 - [LCAT_HUMAN]	7.00	7.00	0.78	0.37
	▽	Q92673	Sortilin-related receptor OS=Homo sapiens GN=SORL1 PE=1 SV=2 - [SORL_HUMAN]	16.00	16.00	0.69	0.37
	▽	O95998	Interleukin-18-binding protein OS=Homo sapiens GN=IL18BP PE=1 SV=2 - [I18BP_HUMAN]	4.00	4.00	0.99	0.37
	▽	Q9Y6W3	Calpain-7 OS=Homo sapiens GN=CAPN7 PE=1 SV=1 - [CAN7_HUMAN]	10.00	10.00	0.58	0.37
	▽	Q12913	Receptor-type tyrosine-protein phosphatase eta OS=Homo sapiens GN=PTPRJ PE=1 SV=3 - [PTPRJ_HUMAN]	11.00	11.00	0.59	0.37
	▽	Q86SR0	Secreted Ly-6/uPAR-related protein 2 OS=Homo sapiens GN=SLURP2 PE=2 SV=1 - [SLUR2_HUMAN]	2.00	2.00	0.56	0.37
	▽	Q08722	Leukocyte surface antigen CD47 OS=Homo sapiens GN=CD47 PE=1 SV=1 - [CD47_HUMAN]	1.00	1.00	0.93	0.37
	▽	O00592	Podocalyxin-like protein 1 OS=Homo sapiens GN=PODXL PE=1 SV=2 - [PODXL_HUMAN]	4.00	4.00	0.59	0.37
	▽	Q96EY5	Multivesicular body subunit 12A OS=Homo sapiens GN=FAM125A PE=1 SV=1 - [F125A_HUMAN]	3.00	3.00	0.63	0.37
	▽	Q8N6Q3	CD177 antigen OS=Homo sapiens GN=CD177 PE=1 SV=2 - [CD177_HUMAN]	6.00	6.00	0.90	0.37
	▽	P78324	Tyrosine-protein phosphatase non-receptor type substrate 1 OS=Homo sapiens GN=SIRPA PE=1 SV=1 - [SHPS	6.00	11.00	0.90	0.37
	▽	Q9NNX6	CD209 antigen OS=Homo sapiens GN=CD209 PE=1 SV=1 - [CD209_HUMAN]	2.00	2.00	0.90	0.37
	▽	Q96FQ6	Protein S100-A16 OS=Homo sapiens GN=S100A16 PE=1 SV=1 - [S10AG_HUMAN]	2.00	2.00	0.55	0.37
	▽	Q96FE7	Phosphoinositide-3-kinase-interacting protein 1 OS=Homo sapiens GN=PIK3IP1 PE=1 SV=2 - [P3IP1_HUMAN	3.00	3.00	0.81	0.37
	▽	O75882	Attractin OS=Homo sapiens GN=ATRN PE=1 SV=2 - [ATRN_HUMAN]	20.00	20.00	0.86	0.37
	▽	Q8N386	Leucine-rich repeat-containing protein 25 OS=Homo sapiens GN=LRRC25 PE=2 SV=2 - [LRC25_HUMAN]	2.00	2.00	0.72	0.37
	▽	Q8N271	Prominin-2 OS=Homo sapiens GN=PROM2 PE=1 SV=1 - [PROM2_HUMAN]	7.00	7.00	0.64	0.37
	▽	Q8N9B8	Ras-GEF domain-containing family member 1A OS=Homo sapiens GN=RASGEF1A PE=2 SV=2 - [RGF1A_H	1.00	1.00	0.73	0.37
	▽	Q5JXA9	Signal-regulatory protein beta-2 OS=Homo sapiens GN=SIRPB2 PE=2 SV=1 - [SIRB2_HUMAN]	2.00	2.00	1.22	0.37
	▽	O75019	Leukocyte immunoglobulin-like receptor subfamily A member 1 OS=Homo sapiens GN=LILRA1 PE=2 SV=1 -	1.00	2.00	0.63	0.37
	▽	Q9UM47	Neurogenic locus notch homolog protein 3 OS=Homo sapiens GN=NOTCH3 PE=1 SV=1 - [NOTC3_HUMAN]	2.00	2.00	0.67	0.37
	▽	Q9UNA0	A disintegrin and metalloproteinase with thrombospondin motifs 5 OS=Homo sapiens GN=ADAMTS5 PE=1 SV	1.00	1.00	0.73	0.36
	▽	P09619	Beta-type platelet-derived growth factor receptor OS=Homo sapiens GN=PDGFRB PE=1 SV=1 - [PGFRB_HUI	4.00	4.00	0.84	0.36
	▽	P20062	Transcobalamin-2 OS=Homo sapiens GN=TCN2 PE=1 SV=3 - [TCO2_HUMAN]	5.00	5.00	0.71	0.36
	▽	O00241	Signal-regulatory protein beta-1 OS=Homo sapiens GN=SIRPB1 PE=1 SV=4 - [SIRB1_HUMAN]	11.00	12.00	0.84	0.36
	▽	Q01973	Tyrosine-protein kinase transmembrane receptor ROR1 OS=Homo sapiens GN=ROR1 PE=2 SV=2 - [ROR1_HU	4.00	5.00	1.05	0.36
	▽	Q9UHR4	Brain-specific angiogenesis inhibitor 1-associated protein 2-like protein 1 OS=Homo sapiens GN=BAIAP2L1 PI	5.00	5.00	0.89	0.36
	▽	Q8WVQ1	Soluble calcium-activated nucleotidase 1 OS=Homo sapiens GN=CANT1 PE=1 SV=1 - [CANT1_HUMAN]	4.00	4.00	0.60	0.36
	▽	Q8WWV6	High affinity immunoglobulin alpha and immunoglobulin mu Fc receptor OS=Homo sapiens GN=FCAMR PE=	3.00	3.00	0.88	0.36

	▽	Q96P63	Serpin B12 OS=Homo sapiens GN=SERPINB12 PE=1 SV=1 - [SPB12_HUMAN]	2.00	2.00	1.21	0.36
	▽	Q9Y287	Integral membrane protein 2B OS=Homo sapiens GN=ITM2B PE=1 SV=1 - [ITM2B_HUMAN]	3.00	3.00	0.50	0.36
	▽	P08183	Multidrug resistance protein 1 OS=Homo sapiens GN=ABCB1 PE=1 SV=3 - [MDR1_HUMAN]	5.00	5.00	0.51	0.36
	▽	P12830	Cadherin-1 OS=Homo sapiens GN=CDH1 PE=1 SV=3 - [CADH1_HUMAN]	19.00	20.00	1.03	0.36
	▽	P50995	Annexin A11 OS=Homo sapiens GN=ANXA11 PE=1 SV=1 - [ANX11_HUMAN]	14.00	14.00	0.62	0.36
	▽	P01210	Proenkephalin-A OS=Homo sapiens GN=PENK PE=1 SV=1 - [PENK_HUMAN]	2.00	2.00	0.89	0.36
	▽	Q68CJ9	Cyclic AMP-responsive element-binding protein 3-like protein 3 OS=Homo sapiens GN=CREB3L3 PE=1 SV=2	3.00	3.00	0.67	0.36
	▽	A6NL88	Protein shisa-7 OS=Homo sapiens GN=SHISA7 PE=2 SV=3 - [SHSA7_HUMAN]	1.00	1.00	0.86	0.35
	▽	Q8NDC0	MAPK-interacting and spindle-stabilizing protein-like OS=Homo sapiens GN=MAPK1IP1L PE=1 SV=4 - [MIS	1.00	1.00	0.76	0.35
	▽	Q9GZM5	Protein YIPF3 OS=Homo sapiens GN=YIPF3 PE=1 SV=1 - [YIPF3_HUMAN]	2.00	2.00	0.77	0.35
	▽	Q8TCZ2	CD99 antigen-like protein 2 OS=Homo sapiens GN=CD99L2 PE=2 SV=1 - [C99L2_HUMAN]	3.00	3.00	0.71	0.35
	▽	Q8N307	Mucin-20 OS=Homo sapiens GN=MUC20 PE=1 SV=2 - [MUC20_HUMAN]	4.00	4.00	0.64	0.35
	▽	Q9UQV4	Lysosome-associated membrane glycoprotein 3 OS=Homo sapiens GN=LAMP3 PE=1 SV=3 - [LAMP3_HUMA	3.00	3.00	0.73	0.35
	▽	P62330	ADP-ribosylation factor 6 OS=Homo sapiens GN=ARF6 PE=1 SV=2 - [ARF6_HUMAN]	2.00	2.00	0.84	0.35
	▽	O95297	Myelin protein zero-like protein 1 OS=Homo sapiens GN=MPZL1 PE=1 SV=1 - [MPZL1_HUMAN]	2.00	2.00	0.71	0.35
	▽	P14138	Endothelin-3 OS=Homo sapiens GN=EDN3 PE=1 SV=1 - [EDN3_HUMAN]	1.00	1.00	0.74	0.35
	▽	P16112	Aggrecan core protein OS=Homo sapiens GN=ACAN PE=1 SV=2 - [PGCA_HUMAN]	4.00	4.00	0.70	0.35
	▽	Q8WUT4	Leucine-rich repeat neuronal protein 4 OS=Homo sapiens GN=LRRN4 PE=1 SV=3 - [LRRN4_HUMAN]	1.00	1.00	0.82	0.35
	▽	Q14393	Growth arrest-specific protein 6 OS=Homo sapiens GN=GAS6 PE=1 SV=2 - [GAS6_HUMAN]	3.00	3.00	0.55	0.35
	▽	P35443	Thrombospondin-4 OS=Homo sapiens GN=THBS4 PE=1 SV=2 - [TSP4_HUMAN]	11.00	11.00	0.74	0.34
	▽	P84095	Rho-related GTP-binding protein RhoG OS=Homo sapiens GN=RHOG PE=1 SV=1 - [RHOG_HUMAN]	1.00	2.00	0.55	0.34
	▽	P19022	Cadherin-2 OS=Homo sapiens GN=CDH2 PE=1 SV=4 - [CADH2_HUMAN]	16.00	16.00	0.81	0.34
	▽	Q12864	Cadherin-17 OS=Homo sapiens GN=CDH17 PE=1 SV=2 - [CAD17_HUMAN]	3.00	3.00	0.74	0.34
	▽	P61026	Ras-related protein Rab-10 OS=Homo sapiens GN=RAB10 PE=1 SV=1 - [RAB10_HUMAN]	4.00	4.00	0.52	0.34
	▽	Q16849	Receptor-type tyrosine-protein phosphatase-like N OS=Homo sapiens GN=PTPRN PE=1 SV=1 - [PTPRN_HUM	5.00	5.00	0.59	0.34
	▽	P29508	Serpin B3 OS=Homo sapiens GN=SERPINB3 PE=1 SV=2 - [SPB3_HUMAN]	4.00	8.00	1.20	0.34
	▽	Q9UBX1	Cathepsin F OS=Homo sapiens GN=CTSF PE=1 SV=1 - [CATF_HUMAN]	2.00	2.00	0.74	0.34
	▽	Q9BYE9	Cadherin-related family member 2 OS=Homo sapiens GN=CDHR2 PE=1 SV=2 - [CDHR2_HUMAN]	17.00	17.00	0.64	0.34
	▽	Q9BXN2	C-type lectin domain family 7 member A OS=Homo sapiens GN=CLEC7A PE=1 SV=1 - [CLC7A_HUMAN]	2.00	2.00	0.60	0.34
	▽	Q9NY26	Zinc transporter ZIP1 OS=Homo sapiens GN=SLC39A1 PE=1 SV=1 - [S39A1_HUMAN]	1.00	1.00	0.64	0.34
	▽	P24530	Endothelin B receptor OS=Homo sapiens GN=EDNRB PE=1 SV=1 - [EDNRB_HUMAN]	1.00	1.00	0.75	0.33
	▽	P98164	Low-density lipoprotein receptor-related protein 2 OS=Homo sapiens GN=LRP2 PE=1 SV=3 - [LRP2_HUMAN	75.00	75.00	0.66	0.33
	▽	Q8WVV5	Butyrophilin subfamily 2 member A2 OS=Homo sapiens GN=BTN2A2 PE=2 SV=2 - [BT2A2_HUMAN]	5.00	5.00	0.79	0.33
	▽	P04745	Alpha-amylase 1 OS=Homo sapiens GN=AMY1A PE=1 SV=2 - [AMY1_HUMAN]	1.00	20.00	0.56	0.33
	▽	Q16769	Glutaminyl-peptide cyclotransferase OS=Homo sapiens GN=QPCT PE=1 SV=1 - [QPCT_HUMAN]	11.00	11.00	0.79	0.33
	▽	Q8WUM4	Programmed cell death 6-interacting protein OS=Homo sapiens GN=PDCD6IP PE=1 SV=1 - [PDC6I_HUMAN	15.00	15.00	0.56	0.33

	▽	P13611	Versican core protein OS=Homo sapiens GN=VCAN PE=1 SV=3 - [CSPG2_HUMAN]	8.00	8.00	0.72	0.33
	▽	P01229	Lutropin subunit beta OS=Homo sapiens GN=LHB PE=1 SV=3 - [LSHB_HUMAN]	1.00	1.00	0.55	0.33
	▽	Q8TBP5	Membrane protein FAM174A OS=Homo sapiens GN=FAM174A PE=2 SV=1 - [F174A_HUMAN]	1.00	1.00	1.05	0.32
	▽	Q06828	Fibromodulin OS=Homo sapiens GN=FMOD PE=1 SV=2 - [FMOD_HUMAN]	1.00	1.00	0.57	0.32
	▽	Q5VW32	BRO1 domain-containing protein BROX OS=Homo sapiens GN=BROX PE=1 SV=1 - [BROX_HUMAN]	6.00	6.00	0.65	0.32
	▽	Q12929	Epidermal growth factor receptor kinase substrate 8 OS=Homo sapiens GN=EPS8 PE=1 SV=1 - [EPS8_HUMA	6.00	6.00	0.66	0.32
	▽	Q2UY09	Collagen alpha-1(XXVIII) chain OS=Homo sapiens GN=COL28A1 PE=2 SV=2 - [COSA1_HUMAN]	2.00	2.00	0.66	0.32
	▽	P10912	Growth hormone receptor OS=Homo sapiens GN=GHR PE=1 SV=1 - [GHR_HUMAN]	2.00	2.00	0.81	0.32
	▽	P29992	Guanine nucleotide-binding protein subunit alpha-11 OS=Homo sapiens GN=GNA11 PE=1 SV=2 - [GNA11_H	2.00	5.00	0.57	0.32
	▽	Q9H4M9	EH domain-containing protein 1 OS=Homo sapiens GN=EHD1 PE=1 SV=2 - [EHD1_HUMAN]	8.00	10.00	0.56	0.32
	▽	Q86TY3	Uncharacterized protein C14orf37 OS=Homo sapiens GN=C14orf37 PE=2 SV=1 - [CN037_HUMAN]	1.00	1.00	0.85	0.32
	▽	Q86UX2	Inter-alpha-trypsin inhibitor heavy chain H5 OS=Homo sapiens GN=ITIH5 PE=2 SV=2 - [ITIH5_HUMAN]	2.00	2.00	0.59	0.32
	▽	P20073	Annexin A7 OS=Homo sapiens GN=ANXA7 PE=1 SV=3 - [ANXA7_HUMAN]	7.00	7.00	0.54	0.32
	▽	P41181	Aquaporin-2 OS=Homo sapiens GN=AQP2 PE=1 SV=1 - [AQP2_HUMAN]	2.00	2.00	0.66	0.32
	▽	Q6GTX8	Leukocyte-associated immunoglobulin-like receptor 1 OS=Homo sapiens GN=LAIR1 PE=1 SV=1 - [LAIR1_HU	3.00	4.00	0.79	0.32
	▽	P23471	Receptor-type tyrosine-protein phosphatase zeta OS=Homo sapiens GN=PTPRZ1 PE=1 SV=4 - [PTPRZ_HUM	2.00	2.00	0.60	0.32
	▽	Q9HAR2	Latrophilin-3 OS=Homo sapiens GN=LPHN3 PE=1 SV=2 - [LPHN3_HUMAN]	1.00	1.00	0.81	0.32
	▽	O75976	Carboxypeptidase D OS=Homo sapiens GN=CPD PE=1 SV=2 - [CBPD_HUMAN]	1.00	1.00	0.75	0.31
	▽	Q9HCU0	Endosialin OS=Homo sapiens GN=CD248 PE=1 SV=1 - [CD248_HUMAN]	12.00	12.00	0.71	0.31
	▽	P40189	Interleukin-6 receptor subunit beta OS=Homo sapiens GN=IL6ST PE=1 SV=2 - [IL6RB_HUMAN]	9.00	9.00	0.76	0.31
	▽	P29323	Ephrin type-B receptor 2 OS=Homo sapiens GN=EPHB2 PE=1 SV=5 - [EPHB2_HUMAN]	6.00	6.00	0.75	0.31
	▽	Q9P121	Neurotrimin OS=Homo sapiens GN=NTM PE=1 SV=1 - [NTRI_HUMAN]	4.00	5.00	0.65	0.31
	▽	P51148	Ras-related protein Rab-5C OS=Homo sapiens GN=RAB5C PE=1 SV=2 - [RAB5C_HUMAN]	3.00	5.00	0.64	0.31
	▽	P35052	Glypican-1 OS=Homo sapiens GN=GPC1 PE=1 SV=2 - [GPC1_HUMAN]	14.00	14.00	0.52	0.31
	▽	Q9UBI6	Guanine nucleotide-binding protein G(I)/G(S)/G(O) subunit gamma-12 OS=Homo sapiens GN=GNG12 PE=1 S	3.00	3.00	0.60	0.31
	▽	Q13873	Bone morphogenetic protein receptor type-2 OS=Homo sapiens GN=BMPR2 PE=1 SV=2 - [BMPR2_HUMAN]	4.00	4.00	0.89	0.31
	▽	Q9HBH0	Rho-related GTP-binding protein RhoF OS=Homo sapiens GN=RHOF PE=2 SV=1 - [RHOF_HUMAN]	3.00	3.00	0.63	0.31
	▽	P00790	Pepsin A OS=Homo sapiens GN=PGA3 PE=1 SV=1 - [PEPA_HUMAN]	4.00	4.00	0.72	0.31
	▽	Q9UBD6	Ammonium transporter Rh type C OS=Homo sapiens GN=RHCG PE=1 SV=1 - [RHCG_HUMAN]	2.00	2.00	0.82	0.31
	▽	Q9UPP1	Histone lysine demethylase PHF8 OS=Homo sapiens GN=PHF8 PE=1 SV=3 - [PHF8_HUMAN]	1.00	1.00	0.70	0.31
	▽	P16070	CD44 antigen OS=Homo sapiens GN=CD44 PE=1 SV=2 - [CD44_HUMAN]	5.00	5.00	1.12	0.31
	▽	O00308	NEDD4-like E3 ubiquitin-protein ligase WWP2 OS=Homo sapiens GN=WWP2 PE=1 SV=2 - [WWP2_HUMA	1.00	1.00	0.52	0.31
	▽	Q9NPF0	CD320 antigen OS=Homo sapiens GN=CD320 PE=1 SV=1 - [CD320_HUMAN]	3.00	3.00	0.79	0.31
	▽	Q8IX04	Ubiquitin-conjugating enzyme E2 variant 3 OS=Homo sapiens GN=UEVLD PE=1 SV=2 - [UEVLD_HUMAN]	4.00	4.00	0.51	0.31
	▽	Q9UKY0	Prion-like protein doppel OS=Homo sapiens GN=PRND PE=1 SV=2 - [PRND_HUMAN]	1.00	1.00	0.64	0.31
	▽	Q16363	Laminin subunit alpha-4 OS=Homo sapiens GN=LAMA4 PE=1 SV=4 - [LAMA4_HUMAN]	3.00	3.00	0.65	0.30

	▽	A8K2U0	Alpha-2-macroglobulin-like protein 1 OS=Homo sapiens GN=A2ML1 PE=1 SV=2 - [A2ML1_HUMAN]	8.00	8.00	0.90	0.30
	▽	P12109	Collagen alpha-1(VI) chain OS=Homo sapiens GN=COL6A1 PE=1 SV=3 - [CO6A1_HUMAN]	31.00	31.00	0.68	0.30
	▽	Q7Z5N4	Protein sidekick-1 OS=Homo sapiens GN=SDK1 PE=1 SV=3 - [SDK1_HUMAN]	1.00	1.00	1.64	0.30
	▽	Q86UN3	Reticulon-4 receptor-like 2 OS=Homo sapiens GN=RTN4RL2 PE=1 SV=1 - [R4RL2_HUMAN]	7.00	7.00	0.70	0.30
	▽	Q4LDE5	Sushi, von Willebrand factor type A, EGF and pentraxin domain-containing protein 1 OS=Homo sapiens GN=S	2.00	2.00	0.60	0.30
	▽	P20336	Ras-related protein Rab-3A OS=Homo sapiens GN=RAB3A PE=1 SV=1 - [RAB3A_HUMAN]	1.00	3.00	0.62	0.30
	▽	Q14332	Frizzled-2 OS=Homo sapiens GN=FZD2 PE=2 SV=1 - [FZD2_HUMAN]	2.00	3.00	0.89	0.30
	▽	P08962	CD63 antigen OS=Homo sapiens GN=CD63 PE=1 SV=2 - [CD63_HUMAN]	2.00	2.00	0.67	0.30
	▽	Q9H9P2	Chondrolectin OS=Homo sapiens GN=CHODL PE=2 SV=2 - [CHODL_HUMAN]	1.00	1.00	0.70	0.29
	▽	Q8WUF5	RelA-associated inhibitor OS=Homo sapiens GN=PPP1R13L PE=1 SV=4 - [IASPP_HUMAN]	1.00	1.00	1.26	0.29
	▽	Q9NU53	Uncharacterized protein C6orf72 OS=Homo sapiens GN=C6orf72 PE=2 SV=1 - [CF072_HUMAN]	5.00	5.00	0.68	0.29
	▽	P20151	Kallikrein-2 OS=Homo sapiens GN=KLK2 PE=2 SV=1 - [KLK2_HUMAN]	2.00	3.00	0.59	0.29
	▽	Q9Y3E7	Charged multivesicular body protein 3 OS=Homo sapiens GN=VPS24 PE=1 SV=3 - [CHMP3_HUMAN]	2.00	2.00	0.56	0.29
	▽	Q92902	Hermansky-Pudlak syndrome 1 protein OS=Homo sapiens GN=HPS1 PE=1 SV=2 - [HPS1_HUMAN]	1.00	1.00	1.40	0.29
	▽	O14798	Tumor necrosis factor receptor superfamily member 10C OS=Homo sapiens GN=TNFRSF10C PE=1 SV=3 - [T	3.00	3.00	0.91	0.29
	▽	Q9H7M9	Platelet receptor Gi24 OS=Homo sapiens GN=C10orf54 PE=1 SV=2 - [GI24_HUMAN]	1.00	1.00	0.64	0.29
	▽	Q86YL7	Podoplanin OS=Homo sapiens GN=PDPN PE=1 SV=3 - [PDPN_HUMAN]	1.00	1.00	0.65	0.28
	▽	P10586	Receptor-type tyrosine-protein phosphatase F OS=Homo sapiens GN=PTPRF PE=1 SV=2 - [PTPRF_HUMAN]	2.00	2.00	0.73	0.28
	▽	P22748	Carbonic anhydrase 4 OS=Homo sapiens GN=CA4 PE=1 SV=2 - [CAH4_HUMAN]	1.00	1.00	0.62	0.28
	▽	Q9H665	Transmembrane protein 149 OS=Homo sapiens GN=TMEM149 PE=2 SV=1 - [TM149_HUMAN]	4.00	4.00	0.71	0.28
	▽	P07947	Proto-oncogene tyrosine-protein kinase Yes OS=Homo sapiens GN=YES1 PE=1 SV=3 - [YES_HUMAN]	3.00	3.00	0.52	0.28
	▽	Q9UQB8	Brain-specific angiogenesis inhibitor 1-associated protein 2 OS=Homo sapiens GN=BAIAP2 PE=1 SV=1 - [BA	2.00	2.00	0.54	0.28
	▽	P30530	Tyrosine-protein kinase receptor UFO OS=Homo sapiens GN=AXL PE=1 SV=3 - [UFO_HUMAN]	7.00	7.00	0.72	0.28
	▽	O00161	Synaptosomal-associated protein 23 OS=Homo sapiens GN=SNAP23 PE=1 SV=1 - [SNP23_HUMAN]	3.00	3.00	0.60	0.28
	▽	Q9H223	EH domain-containing protein 4 OS=Homo sapiens GN=EHD4 PE=1 SV=1 - [EHD4_HUMAN]	7.00	9.00	0.70	0.28
	▽	P15328	Folate receptor alpha OS=Homo sapiens GN=FOLR1 PE=1 SV=3 - [FOLR1_HUMAN]	5.00	6.00	0.63	0.27
	▽	Q99835	Smoothened homolog OS=Homo sapiens GN=SMO PE=1 SV=1 - [SMO_HUMAN]	4.00	4.00	0.66	0.27
	▽	Q8NFT8	Delta and Notch-like epidermal growth factor-related receptor OS=Homo sapiens GN=DNER PE=1 SV=1 - [DN	2.00	2.00	0.70	0.27
	▽	P22891	Vitamin K-dependent protein Z OS=Homo sapiens GN=PROZ PE=1 SV=2 - [PROZ_HUMAN]	10.00	10.00	0.68	0.27
	▽	O75487	Glypican-4 OS=Homo sapiens GN=GPC4 PE=1 SV=4 - [GPC4_HUMAN]	5.00	6.00	0.57	0.27
	▽	Q9P2B2	Prostaglandin F2 receptor negative regulator OS=Homo sapiens GN=PTGFRN PE=1 SV=2 - [FPRP_HUMAN]	4.00	4.00	0.64	0.27
	▽	Q9NP79	Vacuolar protein sorting-associated protein VTA1 homolog OS=Homo sapiens GN=VTA1 PE=1 SV=1 - [VTA1	4.00	4.00	0.50	0.27
	▽	Q969X1	Transmembrane BAX inhibitor motif-containing protein 1 OS=Homo sapiens GN=TMBIM1 PE=1 SV=2 - [TM	2.00	2.00	0.56	0.27
	▽	Q99574	Neuroserpin OS=Homo sapiens GN=SERPINI1 PE=1 SV=1 - [NEUS_HUMAN]	6.00	6.00	0.60	0.26
	▽	O75015	Low affinity immunoglobulin gamma Fc region receptor III-B OS=Homo sapiens GN=FCGR3B PE=1 SV=2 - [	1.00	2.00	0.87	0.26
	▽	P28908	Tumor necrosis factor receptor superfamily member 8 OS=Homo sapiens GN=TNFRSF8 PE=1 SV=1 - [TNR8_	1.00	1.00	1.17	0.26

	▽	Q96M61	Melanoma-associated antigen B18 OS=Homo sapiens GN=MAGEB18 PE=1 SV=2 - [MAGBI_HUMAN]	1.00	1.00	0.97	0.26
	▽	Q03403	Trefoil factor 2 OS=Homo sapiens GN=TFF2 PE=1 SV=2 - [TFF2_HUMAN]	4.00	4.00	0.80	0.26
	▽	P05091	Aldehyde dehydrogenase, mitochondrial OS=Homo sapiens GN=ALDH2 PE=1 SV=2 - [ALDH2_HUMAN]	1.00	2.00	1.22	0.26
	▽	P60033	CD81 antigen OS=Homo sapiens GN=CD81 PE=1 SV=1 - [CD81_HUMAN]	1.00	1.00	0.69	0.26
	▽	Q6UY11	Protein delta homolog 2 OS=Homo sapiens GN=DLK2 PE=2 SV=1 - [DLK2_HUMAN]	4.00	4.00	0.84	0.25
	▽	Q9BZZ2	Sialoadhesin OS=Homo sapiens GN=SIGLEC1 PE=1 SV=2 - [SN_HUMAN]	3.00	3.00	0.77	0.25
	▽	Q6DKI7	Transmembrane protein PVRIG OS=Homo sapiens GN=PVRIG PE=2 SV=1 - [PVRIG_HUMAN]	1.00	1.00	0.78	0.25
	▽	P12319	High affinity immunoglobulin epsilon receptor subunit alpha OS=Homo sapiens GN=FCER1A PE=1 SV=1 - [F	1.00	1.00	0.57	0.25
	▽	P15941	Mucin-1 OS=Homo sapiens GN=MUC1 PE=1 SV=3 - [MUC1_HUMAN]	8.00	8.00	0.84	0.25
	▽	Q8IYS5	Osteoclast-associated immunoglobulin-like receptor OS=Homo sapiens GN=OSCAR PE=2 SV=2 - [OSCAR_H	4.00	4.00	0.75	0.25
	▽	Q07092	Collagen alpha-1(XVI) chain OS=Homo sapiens GN=COL16A1 PE=1 SV=2 - [COGA1_HUMAN]	1.00	1.00	0.60	0.25
	▽	P48594	Serpin B4 OS=Homo sapiens GN=SERPINB4 PE=1 SV=2 - [SPB4_HUMAN]	2.00	6.00	0.75	0.24
	▽	Q9HBB8	Cadherin-related family member 5 OS=Homo sapiens GN=CDHR5 PE=1 SV=3 - [CDHR5_HUMAN]	4.00	4.00	0.89	0.24
	▽	P53990	IST1 homolog OS=Homo sapiens GN=KIAA0174 PE=1 SV=1 - [IST1_HUMAN]	11.00	11.00	0.59	0.24
	▽	Q9NY72	Sodium channel subunit beta-3 OS=Homo sapiens GN=SCN3B PE=1 SV=1 - [SCN3B_HUMAN]	1.00	1.00	0.61	0.24
	▽	Q8N2G4	Ly6/PLAUR domain-containing protein 1 OS=Homo sapiens GN=LYPD1 PE=1 SV=2 - [LYPD1_HUMAN]	1.00	1.00	0.55	0.24
	▽	Q99075	Proheparin-binding EGF-like growth factor OS=Homo sapiens GN=HBEGF PE=1 SV=1 - [HBEGF_HUMAN]	1.00	1.00	0.70	0.24
	▽	P08138	Tumor necrosis factor receptor superfamily member 16 OS=Homo sapiens GN=NGFR PE=1 SV=1 - [TNR16_H	4.00	4.00	0.62	0.24
	▽	P36639	7,8-dihydro-8-oxoguanine triphosphatase OS=Homo sapiens GN=NUDT1 PE=1 SV=3 - [8ODP_HUMAN]	4.00	4.00	0.66	0.23
	▽	P62070	Ras-related protein R-Ras2 OS=Homo sapiens GN=RRAS2 PE=1 SV=1 - [RRAS2_HUMAN]	1.00	1.00	0.56	0.23
	▽	Q53GD3	Choline transporter-like protein 4 OS=Homo sapiens GN=SLC44A4 PE=2 SV=1 - [CTL4_HUMAN]	4.00	4.00	0.57	0.23
	▽	P43115	Prostaglandin E2 receptor EP3 subtype OS=Homo sapiens GN=PTGER3 PE=2 SV=1 - [PE2R3_HUMAN]	1.00	1.00	0.70	0.23
	▽	O95467	Neuroendocrine secretory protein 55 OS=Homo sapiens GN=GNAS PE=2 SV=1 - [GNAS3_HUMAN]	1.00	1.00	0.67	0.22
	▽	Q9UNN8	Endothelial protein C receptor OS=Homo sapiens GN=PROCR PE=1 SV=1 - [EPCR_HUMAN]	4.00	4.00	0.92	0.22
	▽	P09758	Tumor-associated calcium signal transducer 2 OS=Homo sapiens GN=TACSTD2 PE=1 SV=3 - [TACD2_HUM	1.00	1.00	0.63	0.22
	▽	Q9UIV8	Serpin B13 OS=Homo sapiens GN=SERPINB13 PE=2 SV=2 - [SPB13_HUMAN]	2.00	2.00	0.51	0.22
	▽	P50148	Guanine nucleotide-binding protein G(q) subunit alpha OS=Homo sapiens GN=GNAQ PE=1 SV=4 - [GNAQ_H	1.00	4.00	0.53	0.22
	▽	Q969L2	Protein MAL2 OS=Homo sapiens GN=MAL2 PE=1 SV=1 - [MAL2_HUMAN]	1.00	1.00	0.62	0.22
	▽	Q99816	Tumor susceptibility gene 101 protein OS=Homo sapiens GN=TSG101 PE=1 SV=2 - [TS101_HUMAN]	7.00	7.00	0.59	0.22
	▽	Q7LBR1	Charged multivesicular body protein 1b OS=Homo sapiens GN=CHMP1B PE=1 SV=1 - [CHM1B_HUMAN]	2.00	2.00	0.83	0.21
	▽	Q96HA4	Uncharacterized protein C1orf159 OS=Homo sapiens GN=C1orf159 PE=2 SV=2 - [CA159_HUMAN]	1.00	1.00	0.66	0.21
	▽	Q14982	Opioid-binding protein/cell adhesion molecule OS=Homo sapiens GN=OPCML PE=1 SV=1 - [OPCM_HUMAN	5.00	6.00	0.60	0.20
	▽	Q01151	CD83 antigen OS=Homo sapiens GN=CD83 PE=1 SV=1 - [CD83_HUMAN]	1.00	1.00	0.57	0.20
	▽	O75144	ICOS ligand OS=Homo sapiens GN=ICOSLG PE=1 SV=2 - [ICOSL_HUMAN]	4.00	4.00	0.69	0.20
	▽	P80370	Protein delta homolog 1 OS=Homo sapiens GN=DLK1 PE=1 SV=3 - [DLK1_HUMAN]	2.00	2.00	1.04	0.20
	▽	Q9NZZ3	Charged multivesicular body protein 5 OS=Homo sapiens GN=CHMP5 PE=1 SV=1 - [CHMP5_HUMAN]	5.00	5.00	0.54	0.20

	▽	O43633	Charged multivesicular body protein 2a OS=Homo sapiens GN=CHMP2A PE=1 SV=1 - [CHM2A_HUMAN]	3.00	3.00	0.63	0.20
	▽	P04746	Pancreatic alpha-amylase OS=Homo sapiens GN=AMY2A PE=1 SV=2 - [AMYP_HUMAN]	4.00	22.00	0.66	0.20
	▽	P41217	OX-2 membrane glycoprotein OS=Homo sapiens GN=CD200 PE=2 SV=3 - [OX2G_HUMAN]	4.00	4.00	0.73	0.19
	▽	P07204	Thrombomodulin OS=Homo sapiens GN=THBD PE=1 SV=2 - [TRBM_HUMAN]	4.00	4.00	0.62	0.19
	▽	O00337	Sodium/nucleoside cotransporter 1 OS=Homo sapiens GN=SLC28A1 PE=2 SV=2 - [S28A1_HUMAN]	1.00	1.00	0.56	0.18
	▽	P01133	Pro-epidermal growth factor OS=Homo sapiens GN=EGF PE=1 SV=2 - [EGF_HUMAN]	34.00	34.00	0.50	0.18
	▽	Q9HCG8	Pre-mRNA-splicing factor CWC22 homolog OS=Homo sapiens GN=CWC22 PE=1 SV=3 - [CWC22_HUMAN	1.00	1.00	0.78	0.18
	▽	Q9UQN3	Charged multivesicular body protein 2b OS=Homo sapiens GN=CHMP2B PE=1 SV=1 - [CHM2B_HUMAN]	4.00	4.00	0.58	0.18
	▽	Q6UXI9	Nephronectin OS=Homo sapiens GN=NPNT PE=2 SV=2 - [NPNT_HUMAN]	1.00	1.00	0.63	0.18
	▽	Q9HD42	Charged multivesicular body protein 1a OS=Homo sapiens GN=CHMP1A PE=1 SV=1 - [CHM1A_HUMAN]	1.00	1.00	0.55	0.17
	▽	P19961	Alpha-amylase 2B OS=Homo sapiens GN=AMY2B PE=1 SV=1 - [AMY2B_HUMAN]	1.00	20.00	0.61	0.17
	▽	P07911	Uromodulin OS=Homo sapiens GN=UMOD PE=1 SV=1 - [UROM_HUMAN]	28.00	28.00	0.93	0.16
	▽	Q9UKR3	Kallikrein-13 OS=Homo sapiens GN=KLK13 PE=2 SV=1 - [KLK13_HUMAN]	3.00	3.00	0.51	0.16
	▽	P31944	Caspase-14 OS=Homo sapiens GN=CASP14 PE=1 SV=2 - [CASPE_HUMAN]	13.00	13.00	1.07	0.16
	▽	P09564	T-cell antigen CD7 OS=Homo sapiens GN=CD7 PE=1 SV=1 - [CD7_HUMAN]	2.00	2.00	0.64	0.15
	▽	O14594	Neurocan core protein OS=Homo sapiens GN=NCAN PE=2 SV=3 - [NCAN_HUMAN]	1.00	1.00	0.60	0.14
	▽	P31151	Protein S100-A7 OS=Homo sapiens GN=S100A7 PE=1 SV=4 - [S10A7_HUMAN]	5.00	5.00	0.95	0.12
